# Reproductive traits and population dynamics of benthic invertebrates indicate episodic recruitment patterns across an Arctic polar front

**DOI:** 10.1002/ece3.7539

**Published:** 2021-05-02

**Authors:** Adam J. Reed, Jasmin A. Godbold, Martin Solan, Laura J. Grange

**Affiliations:** ^1^ School of Ocean and Earth Science National Oceanography Centre Southampton University of Southampton Southampton UK; ^2^ School of Ocean Sciences Bangor University Bangor UK

**Keywords:** functional biogeography, gametogenesis, interannual variability, life history, reproductive plasticity

## Abstract

Climate‐induced changes in the ocean and sea ice environment of the Arctic are beginning to generate major and rapid changes in Arctic ecosystems, but the effects of directional forcing on the persistence and distribution of species remain poorly understood. Here, we examine the reproductive traits and population dynamics of the bivalve *Astarte crenata* and sea star *Ctenodiscus crispatus* across a north–south transect that intersects the polar front in the Barents Sea. Both species present large oocytes indicative of short pelagic or direct development that do not differ in size–frequency between 74.5 and 81.3º latitude. However, despite gametogenic maturity, we found low frequencies of certain size classes within populations that may indicate periodic recruitment failure. We suggest that recruitment of *A. crenata* could occur periodically when conditions are favorable, while populations of *C. crispatus* are characterized by episodic recruitment failures. Pyloric caeca indices in *C. crispatus* show that food uptake is greatest at, and north of, the polar front, providing credence to the view that interannual variations in the quantity and quality of primary production and its flux to the seafloor, linked to the variable extent and thickness of sea ice, are likely to be strong determinants of physiological fitness. Our findings provide evidence that the distribution and long‐term survival of species is not only a simple function of adaptive capacity to specific environmental changes, but will also be contingent on the frequency and occurrence of years where environmental conditions support reproduction and settlement.

## Graphical abstract

Using functionally important sea star and bivalve species from the high Arctic seafloor, we examine reproduction and population dynamics across a north–south transect intersecting the polar front and show that there is no difference in reproductive traits across the polar front. However, population dynamics suggest recruitment may be episodic. Our analyses reveal that long‐term viability of species is contingent on the occurrence of years where environmental conditions support reproduction and settlement.

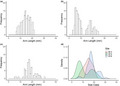

## 1 INTRODUCTION

The Arctic is widely considered to be one of the most rapidly warming regions in the world, resulting from regional amplifications of global climate change (Hoegh‐Guldberg and Bruno, 2010). Increasing sea surface temperatures (Polyakov, Pnyushkov, et al., 2012) and dramatic reduction in summer sea ice extent and thickness (Comiso, 2012; Steele et al., 2008; Stroeve and Notz, 2018) correspond to the northward range expansion of many boreal invertebrate species that demonstrate generalist resource use and ecological plasticity (De Cesare et al., 2017; Frainer et al., 2017; Matishov et al., 2012). There is a broad understanding of which species are most vulnerable to climate change (e.g., Kroeker et al., 2013; Peck, 2016; Wassmann et al., 2011) and how species may respond through migration or plasticity (Frainer et al., 2017; Thyrring et al., 2015). However, the survival of populations is not solely dependent on the tolerance of individuals to change, but also on the ability to reproduce and recruit to the environment (Przeslawski et al., 2015), without significant trade‐offs with growth or fecundity (Reed et al., 2014).

Understanding these knowledge gaps is vital in polar environments, where species may be long‐lived (Moss et al., 2016; Olivier et al., 2020), have episodic reproductive events (Dayton et al., 2016), or only recruit after extended gametogenic cycles or larval development (Lau et al., 2018; Moran et al., 2019). Indeed, the vast array of development modes and gametogenic responses to the environment make it impossible to project the influence of change on species‐specific life history without direct observation (Marshall et al., 2012). While there have been numerous studies on reproductive trait variability across wide latitudinal ranges, local variability is often ignored (Lester et al., 2007; Reed et al., 2014). However, evidence of species resilience through plasticity to regional and subtle environmental variations could still provide essential information to understanding the future distribution of benthic macrofauna (Byrne, 2011) and the maintenance of ecosystem functioning (Gogina et al., 2020; McLean et al., 2018).

Arctic benthic fauna originates from both boreal and arctic distributions (Jørgensen et al., 2015; Piepenburg, 2005), and the contrast in the timings and type of available food are reflected by the biogeographic spread of boreal generalists with feeding plasticity (De Cesare et al., 2017; Fossheim et al., 2015). Alterations in food supply associated with thinner sea ice (sea ice algal input, Boetius et al., 2013; Lange et al., 2019; timing of phytoplankton bloom, Arrigo et al., 2008, Kohlbach et al., 2016; and pelagic–benthic coupling, Tamelander et al., 2006; Kędra et al., 2015) impact both the physiology of benthic species (Ambrose et al., 2006; Carroll et al., 2014) and the remineralization of organic matter at the seafloor (Macdonald et al., 2015). This is important because whole animal physiology, including reproduction, is often tightly coupled with food availability and quality (Campanyà‐Llovet et al., 2017; Mayor et al., 2009), and metabolic rate is determined by food rather than temperature at low temperatures (Blicher et al., 2010; Brockington and Clarke, 2001). Hence, for some species, this raises the possibility that the indirect effects of climate change on food quality could lead to amplified species declines under future ice retreat scenarios (Murdoch et al., 2020).

The polar front in the Barents Sea acts as an oceanographic barrier, creating a boundary between the relatively warm (>0°C) Atlantic water and cold (<0°C) Arctic water (Loeng, 1991). The interface between these water masses is characterized by enhanced primary production (Wassmann et al., 2006), and therefore, species distributions across this region may be influenced spatially by both temperature and primary production. While changes to invertebrate growth across the polar front (Carroll et al., 2011) and wide thermal tolerances of Arctic‐boreal species (Richard et al., 2012) have been previously identified, there is very little information about reproductive trait variation across the region. Periodic invasions of reproductively inactive bivalves to Arctic coastlines (Thyrring et al., 2015) and reproductive regression in temperate species of krill in the Arctic (Huenerlage et al., 2015) suggest that there may be part of a species range where adults are not reproductive or only periodically recruited (Przeslawski et al., 2015). With an increasing Atlantic influence in the Barents Sea and projected instability of the polar front (Barton et al., 2018), understanding species reproduction and life history is essential for understanding how local environmental changes will affect future generations of benthic populations.

Here, we describe the reproductive and population traits of two key Arctic‐boreal benthic species, the bivalve *Astarte crenata* and sea star *Ctenodiscus crispatus*, across the Barents Sea Polar Front. As highly abundant Arctic‐boreal species (Jørgensen et al., 2015; Solan et al., 2020), the lecithotropic and direct developing reproductive traits of these infaunal species are also representative of the bivalve and echinoderm‐rich fauna of the Barents Sea (Marshall et al., 2012). To assess for reproductive trait variability, we investigate spatial patterns in gonadal investment and gametogenic development across the polar front, and infer recruitment from population dynamics. We anticipate reproductive traits and recruitment to be driven by the spatial distribution of food quantity and quality across the polar front, as inferred from interannual variations in sea ice extent and related primary production flux to the seafloor.

## 2 METHODS

### 2.1 Sample collection

Specimens of *A. crenata* and *C. crispatus* were collected in July 2017 (JR16006, RRS James Clark Ross; Hopkins, 2018) using a 1.25 m Agassiz trawl (AGT) towed for 15 min at a ship speed of 1 knot, at three stations along the 30E meridian across the approximate location of the Polar Front (B13, 74°49 *N* (South of the polar front); B14, 76°50 *N* (approximate location of the polar front); B16, 80°06 *N* (North of the polar front); see Table S1 and Figure S1 in Solan et al., 2020). The Barents Sea in this region is experiencing rapid warming and reductions in sea ice, which affect annual sea ice extent (Lind et al., 2018). Four trawls were conducted at each station to ensure sufficient spatial replication (Table S1). *Ctenodiscus crispatus* were found at all stations sampled, whereas *A. crenata* were only found in sufficient numbers for analysis at stations B13 and B16. Fauna was sieved over a 1‐cm mesh and retained and fixed in 10% phosphate‐buffered formalin (4% formaldehyde) prior to morphological and histological examination.

### 2.2 Study species

The seastar, *Ctenodiscus crispatus* (Bruzelius, 1805), is one of the eight benthic species reported as biomass‐dominant (>50% of the total benthic biomass) in the Barents Sea (Solan et al., 2020; Jørgensen et al., 2015; Wassmann et al., 2006). This conspicuous species is widespread across the northern high latitudes in both North Atlantic and Arctic waters and throughout soft muddy sediments (Johannesen et al., 2017; Jørgensen et al., 2015; Figure S2a), where it constructs semi‐permanent burrows and feeds nonselectively by subsurface deposit feeding (Shick et al., 1981). Reproduction in *C. crispatus* from other populations has been described as continuous, with a superimposed increase in reproductive intensity associated directly with phytodetrital input from the surface (Falk‐Petersen, 1982; Shick et al., 1981), while its South Atlantic deep‐sea congener *C. australis* is a continuous brooding species (Rivadeneira et al., 2017).

The infaunal bivalve *Astarte crenata* is also dominant in the Barents Sea and found throughout the Atlantic/Arctic boundary, with regionally high abundances (19 ind. 0.5 m^‐2^, Cochrane et al., 2009; Figure S2b) and a life span up to ~48 years (Moss et al., 2018). Details of reproduction are poorly understood, but broadly align with *A. borealis* and *A. elliptica*, which show mature oocytes up to 200 µm diameter throughout the year, with an underlying seasonal intensity in reproduction, and short pelagic larval stages or direct development (Reed et al., 2021; Von Oertzen, 1972).

#### 2.2.1 Morphology and dissection

##### Astarte crenata

To assess population dynamics, each individual (*n* = 159) was measured using a digital calliper (± 0.01mm), to record maximum shell length, height, and width. Soft tissue was removed from the shell with a scalpel and weighed (± 0.01 g). Observation of the dissected bivalves and a preliminary histological analysis revealed that this population of *A. crenata* do not have discrete gonads, but have germinal tissues infiltrating the visceral mass, particularly within the digestive diverticulum. This means that gonad index cannot be reliably calculated. Hence, to ensure reproductive maturity had been reached, only specimens >20 mm shell length were used for reproductive analysis (Von Oertzen, 1972). Whole animal histology was necessary, and it was not possible to calculate a gonad index or measure of energy storage in the digestive diverticulum.

##### Ctenodiscus crispatus

To assess population dynamics, reproductive, and digestive condition of each animal, we measured each specimen and calculated gonad and pyloric caeca indices. We evacuated sediment within the body cavity through the mouth by applying pressure to the dorsal surface while rinsing with seawater. In total, 324 individuals were measured (±0.01 mm) from the center of the mouth to the tip of the longest arm, and from the center of the mouth to the madreporite inter‐radius (Shick et al., 1981), and blot‐weighed (±0.01 g). Dissection of the dorsal epithelium of 151 individual *C. crispatus* above 15 mm arm length revealed the pyloric caeca and gonads as discrete paired organs, which were subsequently removed from two inter‐radial sections by dissection, and used to determine the total gonad and pyloric caeca indices, that is, the ratio of gonad or pyloric caeca mass to whole body wet weight, expressed as percentage. The relationship between gonad and pyloric caeca indices, unique to echinoderms, is a simple and effective means of quantifying reproductive effort, where resources stored in, and mobilized from, the pyloric caeca play an integral role in the provision of energy for gametogenesis (McClintock, 1989).

### 2.3 Histology

Whole animal and reproductive tissues of *A. crenata* (*n* = 52, stations B13 and B16) and *C. crispatus* (*n* = 52, stations B13, B14 and B16) were processed for histology following standard protocols (Lau et al., 2018). Briefly, tissue was dehydrated in graded isopropanol, cleared in xylene, and, depending on tissue size, embedded into 25 × 50 mm or 5 × 5 mm wax blocks. Embedded tissue was cut at 6 µm, mounted onto slides and stained using hematoxylin Z (CellPath), counterstained with eosin Y (CellPath), and immediately cover‐slipped using a DPX mounting medium (Sigma‐Aldrich). Reproductive features were captured using a Nikon D5000 camera mounted on an Olympus (BH‐2) stereomicroscope.

Sections of *A. crenata* demonstrated dense areas of gametogenesis. To ensure near‐maximum cross‐sectional diameter was quantified, unique oocytes were measured only when a nucleus was visible. For *C. crispatus*, as the nucleus remained visible across multiple 6‐µm sections, oocytes were only measured when the nucleolus was visible. For comparison of oocyte sizes between each female and station (*A. crenata*, *n* = 24 (B13, *n* = 12; B16, *n* = 12); *C. crispatus*, *n* = 24 (B13, *n* = 8; B14, *n* = 8; B16, *n* = 8), we calculated the equivalent circular diameter (ECD) (Lau et al., 2018) by measuring the area of 100 oocytes of each female (i.e., 1,200 and 800 oocytes per station) using ImageJ v 1.48 (Schneider et al., 2012):

$$\text{ECD}=2\surd \frac{A}{\pi }$$

where *A* is the area of a measured oocyte (*µ*m^2^). This method assumes the spherical diameter of any shape and is equivalent to the oocyte Feret diameter used in previous studies (Higgs et al., 2009; Reed et al., 2013).

### 2.4 Data analysis

To determine whether there was a difference in pyloric caeca and gonad index along the transect, we conducted a one‐way ANOVA with station (3 levels, B13, B14, and B16) as a nominal explanatory variable with a post hoc Tukey comparison test. Model assumptions were assessed visually for normality (Q–Q plot), homogeneity of variance (plotted residuals versus fitted values), and the presence of outliers or overly influential data points (Cook's distance) (Zuur et al., 2009). Individual Kolmogorov–Smirnov (K‐S) tests were then used for both species, at all stations, to determine whether the size–frequency distribution of oocytes (ECD) differed between pairs of stations (Neat and Burns, 2010). Individual oocyte size–frequency distributions are shown in Figures S3 and S4 (*A. crenata*) and Figures S5–S7 (*C. crispatus*). Gaussian kernal density estimates were plotted for each length adult size–frequency distribution to visually distinguish differences in frequency distributions between stations.

All statistical analyses were performed in R (R Develpment Core Team, 2018). The *fishmethods* package (Nelson, 2019) was used for analysis of the length–frequency distribution and the Kolmogorov–Smirnov test.

## 3 RESULTS

### 3.1 Reproduction

#### 3.1.1 *Astarte* *crenata*

**FIGURE 1:**
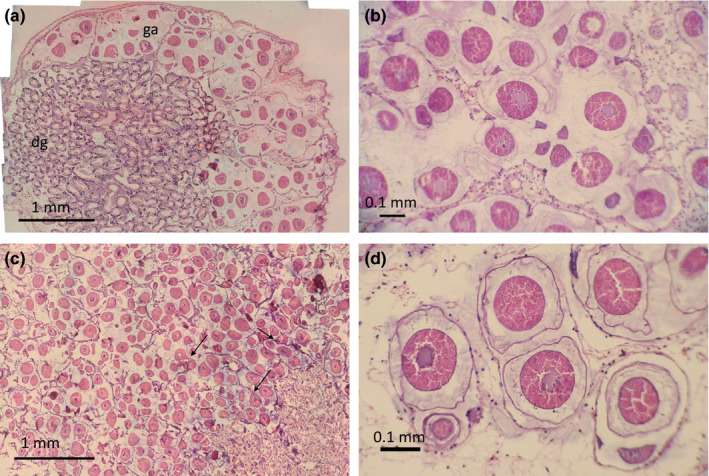
Transverse histology sections of *Astarte crenata* from the Barents Sea. (a) Composite image from a 25.36‐mm shell length individual from station B16 showing gonadal alveoli and digestive diverticula; (b) oocyte development in a 21.90‐mm shell length individual from station B16; (c) high density of oocytes in a 30.1‐mm shell length individual; (d) gelatinous mucous layer surrounding mature previtellogenic oocytes. dg, digestive diverticula; ga, gonadal alveoli; arrows indicate pedunculated oocytes

Examination of the reproductive organs identifies *A. crenata* as a gonochoristic species with reproductive organs found in all but one of the 52 dissected specimens. Size of sexual maturity was not explicitly explored, but male reproductive organs were present in two specimens of 14.23 and 15.84 mm. In total, 27 females (B13, 21.65–27.94 mm; B16, 20.81–30.75 mm) and 24 males (B13, 14.23–28.62 mm; B16, 24.15–31.13 mm) were identified after histological examination. The reproductive tissue in both sexes was found to infiltrate the digestive diverticula, and female reproductive organs consisted of interconnected gonadal alveoli (Figure 1a). All developing stages of oocyte maturity were observed with basophilic previtellogenic oocytes developing alongside acidophilic vitellogenic oocytes in all specimens (Figure 1b).

**FIGURE 2:**
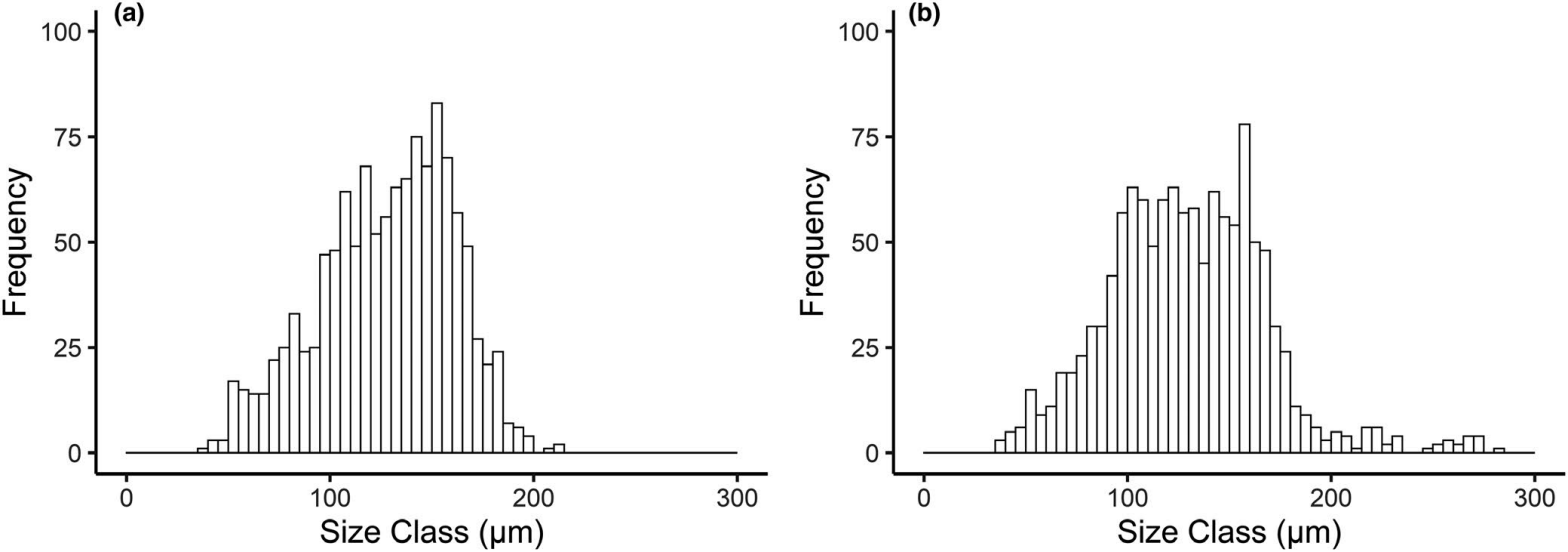
*Astarte crenata* oocyte size–frequency histograms. (a) oocyte size–frequency from station B13, south of the polar front; (b)oocyte size–frequency from station B16, north of the polar front

Previtellogenic oocytes were usually attached to the gonadal epithelium, while vitellogenic oocytes were often pedunculated and attached to the gonadal epithelium by a chord‐like structure (Figure 1c). The largest oocytes were enclosed by a distinctive gelatinous layer (Figure 1d) and observed with empty space between oocytes. Measured oocyte diameters were between 38.65 and 214.25 µm at B13 (mean 127.99 µm ± 32.83 *SD*) and between 36.23 and 281.21 µm at B16 (mean 129.33 µm ± 38.71 *SD*). Oocytes >200 µm were observed at both stations; however, these represented only 0.25% (3 of 1,200) of oocytes at station B13 compared with 3.75% (45 of 1,200) at station B16 (Figure 2). We observed four peaks of oocytes at station B13 (centered ~40 µm, ~70 µm, ~110 µm, and ~175 µm oocyte diameter), but were unable to define any distinct peaks at station B16. Notably, the oocyte size–distributions were not station‐specific (2‐tailed K‐S test, *D*_(195)_ = 0.058, *p* = 0.99). However, the oocyte frequency plots show the highest frequency peak centered at ~175 µm B13 (Figure 2a) compared with a broad peak between 95 and 175 µm (Figure 2b).

#### 3.1.2 Ctenodiscus crispatus

**FIGURE 3:**
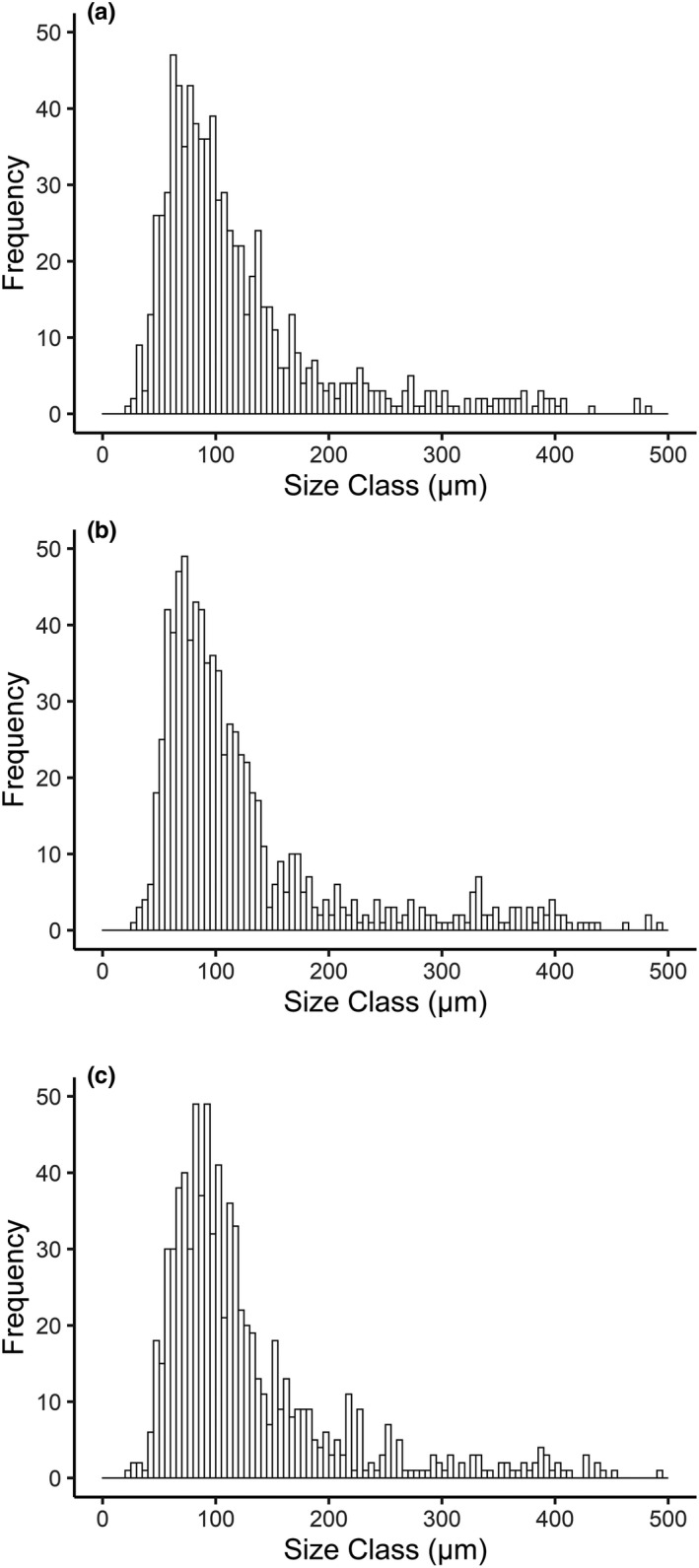
*Ctenodiscus crispatus* oocyte size–frequency histograms. (a) oocyte size–frequency from station B13, south of the polar front; (b) oocyte size–frequency from station B14, approximate location of the polar front; (c) oocyte size–frequency from station B16, north of the polar front

Dissection revealed gonadal tissue in 150 *C. crispatus* with arm lengths of 13.72 – 30.86 mm. Histology identified 26 females and 26 males at sexual maturity from all processed specimens. There was no evidence of hermaphroditism or protandry with both sexes present for specimens with arm lengths between 16.8 and 21.7 mm at B13, 19.8–24.2 mm at B14, or 18.9–27.1 mm at B16. Gonads were paired and discrete, extending from the inter‐radial space between the arms and orientated toward the mouth in finger‐like protrusions. Measured oocyte diameters were between 24.82 and 483.03 µm at B13 (mean 120.33 µm ± 77.79 *SD*; Figure 3a), 27.02 and 491.03 µm at B14 (mean 124.60 µm ± 85.68 *SD*; Figure 3b), and 24.41 and 500.61 µm at B16 (mean 127.67 µm ± 80.41 *SD*; Figure 3c). The size distribution of oocytes was independent of station (K‐S test, B13 versus B14 *D*_(50)_ = 0.036, *p* = 1.000; B13 versus B16 *D*_(50)_ = 0.073, *p* = 1.000; and B14 versus B16 *D*_(50)_ = 0.069, *p* = 1.000), and we were unable to unambiguously detect cohorts in the oocyte size–frequency plots (Figure 3).

**FIGURE 4:**
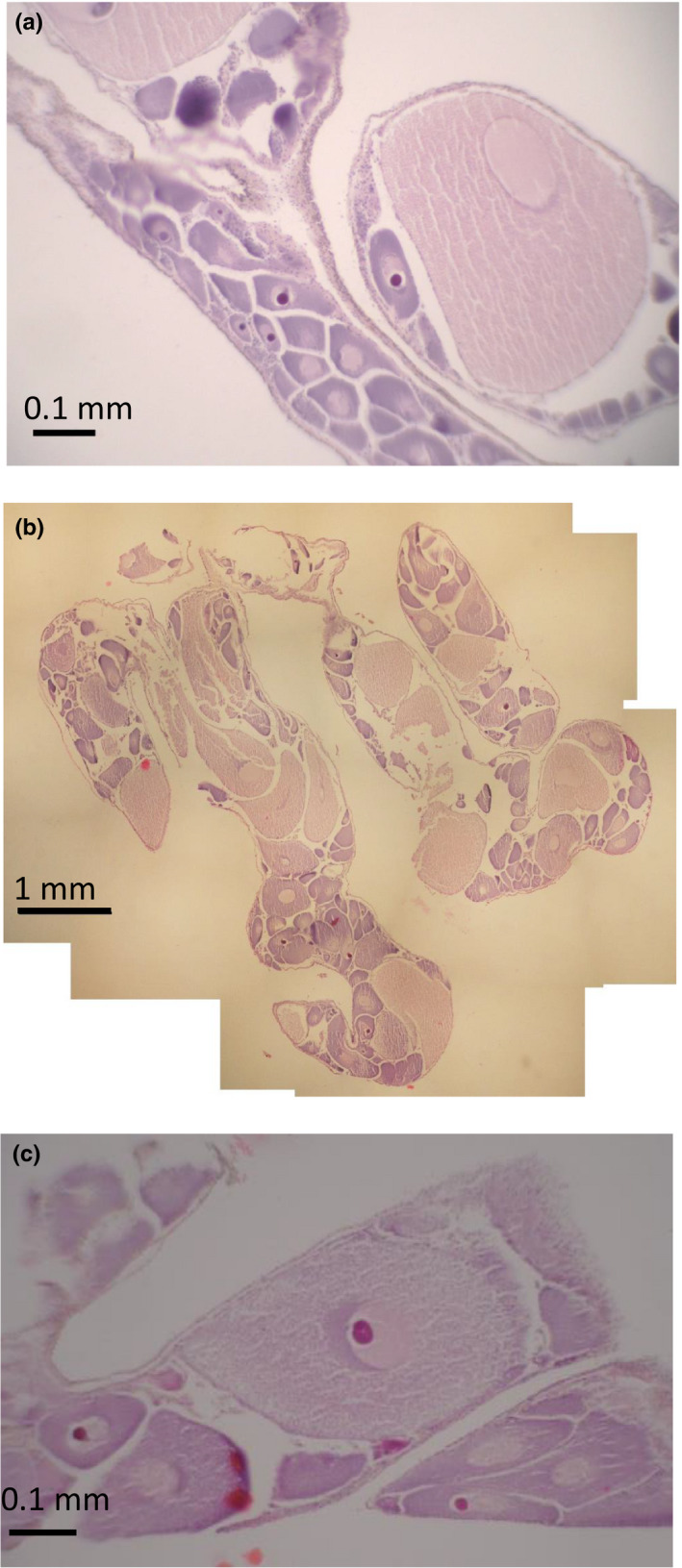
Histology sections of the dissected gonad from *Ctenodiscus crispatus* from the Barents Sea. (a) Small oocytes developing around large vitellogenic oocytes; (b) composite image showing an overview of a complete section of gonad showing narrow finger‐like structure and oocytes of different developmental stage; (c) vitellogenic oocyte showing signs of atresia and cell wall deterioration

Small previtellogenic oocytes were observed developing next to large vitellogenic oocytes (Figure 4a), but the distributional order was not by size, and showed clustering (Figure 4a). Smaller oocytes (<250 µm, staining dark purple) were characteristically basophilic, after which they became acidophilic and had a granular, yolky appearance (Figure 4b). Some of the larger oocytes (~>200 µm) showed signs of atresia (degeneration and reabsorption) with dark stained regions, loss of defined cell membrane, and a general appearance of cell breakdown (Figure 4c). All females presented mature vitellogenic oocytes (>300 µm diameter).

**FIGURE 5:**
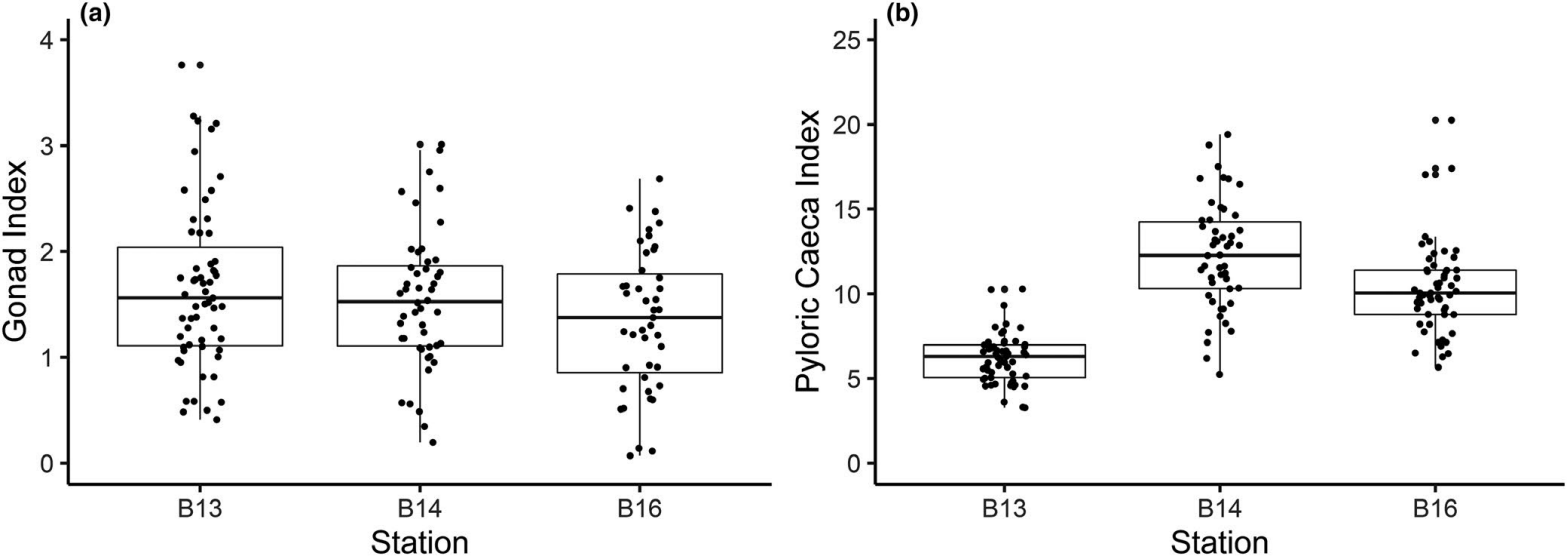
Gonad and pyloric caeca index of *Ctenodiscus crispatus* from the Barents Sea. (a) Gonad index of *C. crispatus* based on wet mass of dissected gonad; (b) pyloric caeca index of *C. crispatus* based on wet mass of dissected pyloric caeca

Mean (± *SD*) gonad index (range, 0.2%–3.76%; Figure 5a) was the lowest at B16 (1.35 ± 0.66%) and highest at B13 (1.66 ± 0.77%) with B14 in‐between (1.54 ± 0.65%) but did not differ between stations (ANOVA, *F*_2, 147_, = 2.409, *p* = 0.0934). Mean gonad index of the females was 1.69 ± 0.70% and 1.51 ± 0.65% for the males. In contrast, we find that mean (± *SD*) pyloric caeca index is dependent on station (ANOVA, *F*_2, 159_ = 81.87, *p* < 0.0001), with particularly low values at station B13 (6.19 ± 1.47%; Tukey, *p* < 0.0001) and particularly high values at B14 (12.27 ± 3.18%; Tukey, *p* = 0.00029) relative to B16 (10.30 ± 2.70%, Figure 5b).

### 3.2 Morphology

**FIGURE 6:**
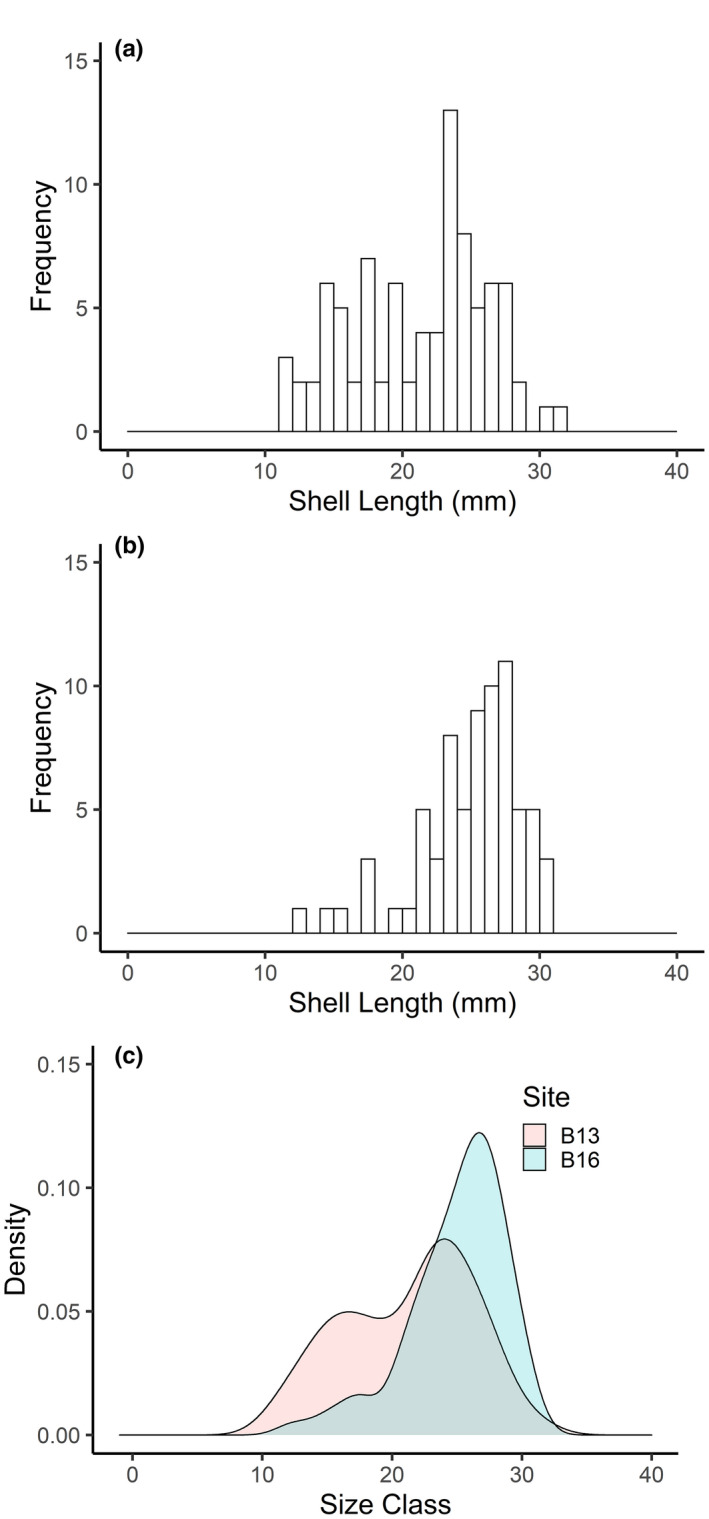
Shell length–size–frequencies of *Astarte crenata* in the Barents Sea. (a) Shell length–size–frequency at station B13, south of the polar front; (b) shell length–size–frequency at station B16, north of the polar front; (c) Gaussian kernal density estimate of the shell length–frequency distributions at stations B13 and B16

**FIGURE 7:**
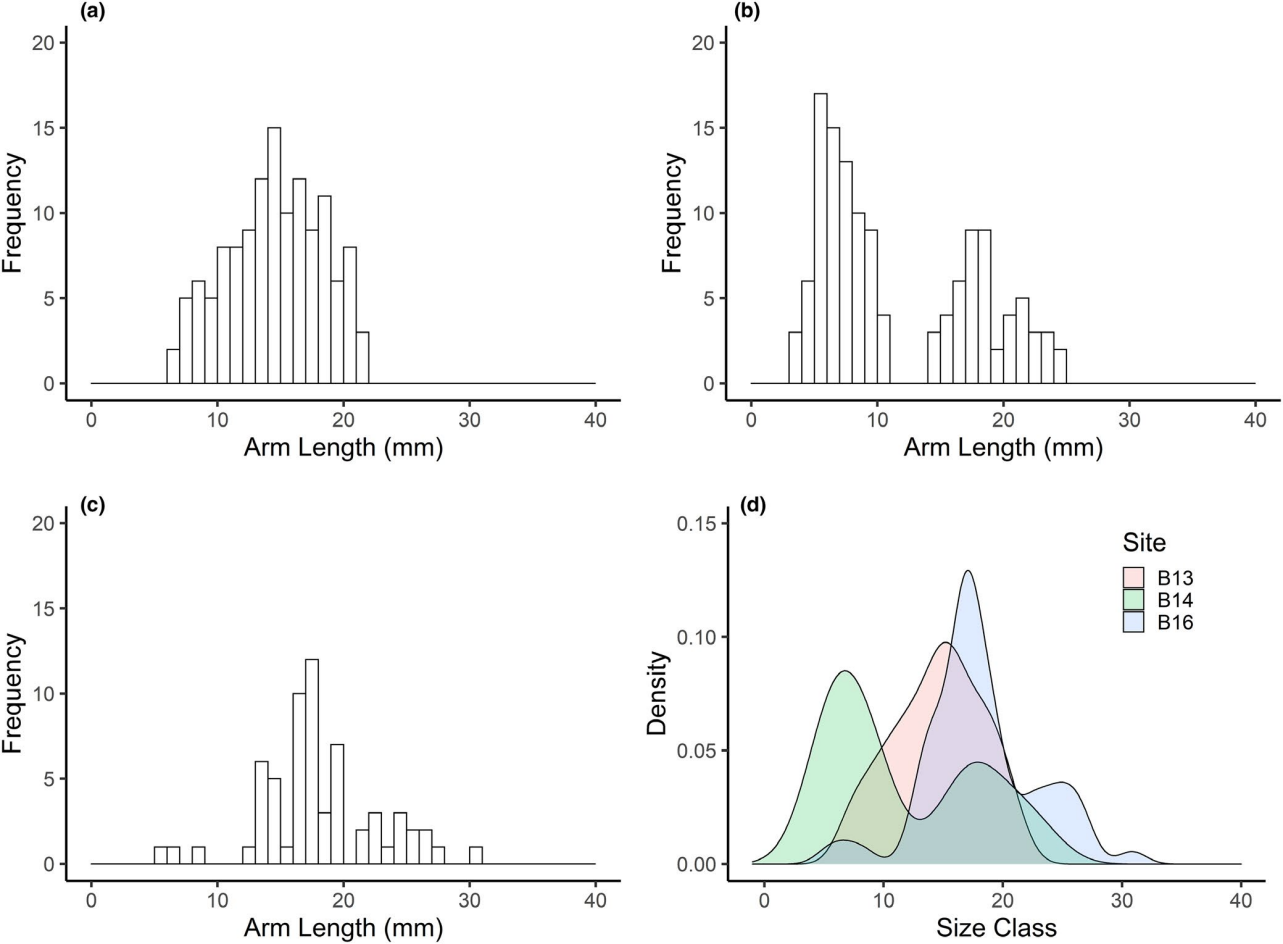
Arm length–size–frequencies of *Ctenodiscus crispatus* from stations across a south–north transect in the Barents Sea. (a) Arm length–frequency from station B13, south of the polar front; (b) arm length–frequency from station B14, approximate location of the polar front; (c) arm length–frequency from station B16, north of the polar front; (d) Gaussian kernal density estimate of the shell length–frequency distributions at stations B13 and B16

The length/height shell morphology of *A. crenata* showed identical patterns at both B13 and B16 (Figure S8); however, there was a higher proportion of specimens >20 mm shell length at B16 compared with B13 (Figure 6), and only four specimens (not included in our analysis) found at B14. In contrast, morphology of sexually mature *C. crispatus* showed heterogeneity in the relationship between arm length and madreporite inter‐radius (ANOVA, *F*_2,151_, = 27.47, *p* < 0.0001), with a post hoc Tukey test identifying individuals at B16 with longer arms to inter‐radius of central disk than those at B14 and B13 (Figure S9, Tukey, *p* < 0.0001). Specimens ranged in size from 6.81 to 21.79 mm at B13, 3.49 to 24.46 mm at B14, and 5.41 to 30.86 mm at B16 (Figure 7). However, there was a notable absence of size classes across all stations, with no specimens with >22 mm arm length at B13 (Figure 7a,d), no specimens between 10.66 and 15.03 mm arm length at B14 (Figure 7b,d), and only three specimens <12.37 mm at B16 (Figure 7c,d).

## 4 DISCUSSION

We have demonstrated, for two representative and functionally important benthic species, a consistency in reproductive traits across the Barents Sea polar front. Our observations that environmental variability across the polar front has little observable effect on oocyte size–frequency distributions are, however, contrary to expectations as there are many reports that benthic invertebrates respond to variations in food supply (Boetius et al., 2013; Søreide et al., 2010) and thermal stability (Huenerlage et al., 2015; Peck, 2016) related to the proximity of the polar front and ice‐edge margin (Carmack and Wassmann, 2006; Tamelander et al., 2006). Both oceanographic features are associated with enhanced levels of primary production and benthic biomass (Carroll et al., 2014; Kędra et al., 2013), and this availability of food is reflected in the increased pyloric caeca index in *C. crispatus* at the polar front. This suggests that environmental variations across the polar front could still be affecting physiology through energetics, and the composition of oocytes, which directly relates to larval size and survival (Caballes et al., 2016). Moreover, low frequency of small shell length *A. crenata* and missing arm length–size classes of *C. crispatus* within and north of the polar front, suggest that reproduction may be affected at alternative life‐history stages after gametogenesis, and that periodic variations in reproductive success—or even recruitment failure—may occur when the prevailing conditions are unfavorable, as observed under experimental conditions (Reed et al., 2021) and in rapidly changing Antarctic regions (Dayton et al., 2019).

The mean oocyte sizes measured for *A. crenata* are similar to those reported for the congeners *A. borealis* and *A. elliptica* (150 – 200 µm; Saleuddin, 1965; Von Oertzen, 1972), and the yolky appearance of the cytoplasm is usually associated with a short pelagic larval development or direct development (Fetzer and Arntz, 2008; Ockelmann et al., 1965). Additionally, the presence of an enveloping “sticky” mucous layer has been observed in all *Astarte* spp. described to date and may fulfill a protective and/or nutritional function (Von Oertzen, 1972), or may be used to adhere to nearby hard substrata either singly or in clusters (Collin and Giribet, 2010). The oocyte frequency distributions of *A. crenata* observed in our study are unable to provide evidence of seasonal or continuous reproduction, although differential patterns in oocyte frequency may suggest a periodic reproductive cycle south of the polar front. This contrasts to persistence of large oocytes north of the polar front, aligned with a recent spawning event and subsequent reabsorption of retained oocytes (Lango‐Reynoso et al., 2000), or lack of spawning altogether. Empty space between oocytes and elongated large oocytes, a characteristic associated with cell breakdown and reabsorption (Lango‐Reynoso et al., 2000), support this interpretation.

The maximum oocyte diameter and range of oocyte sizes of *C. crispatus* are consistent with findings from populations in the Gulf of Maine, N.W. Atlantic (Shick et al., 1981), Conception Bay, Canada (Jaramillo, 2001), and Ramfjorden, Norway (Falk‐Petersen, 1982). The large oocyte sizes are akin to polar invertebrates, which undergo direct development or brooding (Ockelmann et al., 1965; Reed et al., 2013), and are not unusual at low water temperatures associated with the polar seas. Although seasonality of oocyte production cannot be reliably determined from a single point in time, the continuous investment into oocyte production, evidenced by occurrence of previtellogenic, vitellogenic, and ripe oocytes (30 – 500+ µm), suggests continuous reproduction. In other populations, reproduction of *C. crispatus* has been described as asynchronous and continuous, with superimposed variation in reproductive effort, attributed by the authors as a response to phytodetrital deposition at the benthos (Jaramillo, 2001; Shick et al., 1981). Although there was no difference in gonad index or oocyte frequency distribution between 74.5 and 81.3º N, in the absence of temporal sampling, we cannot rule out gonad proliferation resulting from episodic and heterogeneous pulses of food and periods of reproductive intensity, as has been identified in other populations (Benítez‐Villalobos and Díaz‐Martínez, 2010; Jaramillo, 2001; Shick et al., 1981; Vardaro et al., 2009).

In polar water, it has been consistently shown that food and not temperature has the greatest effect on organism physiology (Blicher et al., 2010; Brockington and Clarke, 2001). Primary production of ice algal origin is of particular importance to Arctic benthic communities as it grows before ice retreat, sinks quickly, and contributes fresh nutrient‐rich organic matter to the benthos (Boetius et al., 2013; Degen et al., 2016), but availability is also dependent on a highly variable sea ice extent (Figure S10). Periodic deposits can impact on benthic biomass (Ambrose et al., 2006; Kędra et al., 2013), reproduction (Boetius et al., 2013), and growth (Blicher et al., 2010; Carroll et al., 2011). Here, evidence from the pyloric caeca index at and north of the polar front suggests that a higher quantity and/or quality of food has been available to the populations recently under the edge of the ice margin, and subsequently stored excess energy (McClintock, 1989). This corresponds to the higher total organic carbon content of the sediment, which is mostly derived from ice algae within the region of study (Stevenson and Abbott, 2019), and evidence of increasing chlorophyll *a* found in sediments north of the polar front (Krajewska et al., 2017; Morata and Renaud, 2008). Previous lipid analysis on pyloric and reproductive organs in *C. crispatus* specifically suggests an importance of fresh diatom material for lipid storage (Parrish et al., 2009), and this energetic store can subsequently be used for metabolic activity during the food‐limited Arctic winter (Agüera and Byrne, 2018; Cossi et al., 2017) and for the maintenance of continuous gamete development (Falk‐Petersen and Sargent, 1982).

The higher proportion of *A. crenata* above 20 mm shell length, and near absence of the species at the polar front (B14), may be indicative of failures to recruit to the local environment despite the maturity of their reproductive organs, as previously identified in the congener *A. borealis* in the White Sea (67°N), which underwent a multidecadal recruitment failure (Skazina et al., 2013). While we accept that trawling is often considered semiquantitative, considerable trawling effort in a given area permits comparisons of species populations (Degen et al., 2016; Fossheim et al., 2015) and provides confidence to the view that sampling bias did not influence the size distributions captured. *Astarte* sp. are known to be slow‐growing but long‐lived, (*A. borealis* 48 and 150 years (Torres et al., 2011), (Moss et al., 2018); *A. moerchi* up to 109 years (Olivier et al., 2020)), while the life span of *C. crispatus* is suggested to be ~20 years (Nilsen et al., 2006). A chronic failure to recruit could therefore result in an aging population but a considerable amount of time before a population collapse (Dayton et al., 2016; Skazina et al., 2013). Assuming a comparable growth rate to *A*. *borealis* in the White Sea, the majority of the *A. crenata* found above the polar front are in excess of 15 years old (Moss et al., 2018) which suggests, in contrast to populations south of the polar front, that recruitment to this region has been limited, and that variable conditions at the polar front can prevent settlement. However, with long life spans, successful recruitment is only required episodically to maintain populations (Dayton et al., 2019), with offspring either recruited from the local population or crossing the polar front during years of greater Atlantic intrusion into the Barents Sea (Årthun et al., 2012; Neukermans et al., 2018).

The dramatic seasonal variations in sea ice with considerable interannual variability (Årthun et al., 2012; Wassmann et al., 2006) (Figure S10) influence the timings of primary productivity and sea ice algal production. A notable characteristic of the Barents Sea is an observed multidecadal oscillation in sea ice variability ranging from 16 to 40 years (Divine and Dick, 2006) and coincides with the anticipated life expectancy of *A. crenata*. A study along a transect off the Kola Peninsula demonstrated a positive correlation of *C. crispatus* biomass with decadal‐scale temperature anomalies, showing a four‐year delayed response to temperature anomalies and associated patterns in sinking organic matter (Frolova et al., 2007). The missing and low frequencies of size classes in our study could be a response to interannual or multidecadal fluctuations in sea surface temperature (Levitus et al., 2009), sea ice conditions (Divine and Dick, 2006), and the corresponding responses of primary productivity in the region (Dalpadado et al., 2014). *A. crenata* typically show a greater dependence on the later summer phytoplankton blooms (Dalpadado et al., 2014; Tamelander et al., 2006), the timing of which may be an essential cue for episodic growth or recruitment (Dayton et al., 2016), but usually occur after ice retreat. These blooms also represent an important food source for seasonal pelagic feeding larvae (Brandner et al., 2017), and while the development of gametes may not be limited by temperature in the Arctic, the change in food availability, and potential for a mismatch between reproductive and resource allocation (Renaud et al., 2008), could shape the future diversity of species above the current position of the polar front.

As the Arctic responds to climate forcing (Grebmeier et al., 2006; Polyakov, Pnyushkov, et al., 2012; Steele et al., 2008) and transitions to sea ice‐free conditions (Leu et al., 2011; Polyakov, Walsh, et al., 2012), the impact on the species diversity, abundance, and composition at all life‐history stages will have dramatic consequences for ecosystem functioning (Frainer et al., 2017; Godbold and Solan, 2009; Kędra et al., 2015). It might therefore be expected that with regional increases in primary production (Arrigo and Dijken, 2015), regular recruitment events of filter‐feeding species from boreal environments may begin to change functional biogeography and increase borealization at the polar front, as already described in Arctic fish communities (Frainer et al., 2017). However, successful recruitment relies on favorable conditions for fertilization, larval development, and settlement, aspects of benthic invertebrate biology that are not understood in this region (Kuklinski et al., 2013).

Uncertainty caused by variations in appropriate food sources for larvae or newly settled offspring is therefore likely to determine the success or failure of local recruitment or growth in polar benthic ecosystems or range expansion (Dayton et al., 2019). An important next step is to understand the relationship between the quality and abundance of different food sources, the somatic and reproductive allocation of Arctic benthic invertebrates, and reproductive physiology and plasticity of gametes and larvae throughout the region. Indeed, the lack of attention devoted to establishing the plasticity of species has been recognized more generally (Solan et al., 2020). Our study also highlights the need to understand the direct and indirect effects of climate change over longer (multigenerational) timescales (Byrne et al., 2020) and to appreciate the complex interactions between the life‐history traits, environmental requirements of organisms and climate change that can, ultimately, determine local extinction risk (Murdoch et al., 2020).

## CONFLICT OF INTEREST

The authors have no conflicts of interest to declare.

## AUTHORS CONTRIBUTION

**Adam Jerold Reed:** Conceptualization (equal); Data curation (lead); Formal analysis (equal); Investigation (lead); Methodology (lead); Visualization (equal); Writing‐original draft (lead); Writing‐review & editing (equal). **Jasmin Annica Godbold:** Formal analysis (equal); Supervision (supporting); Visualization (supporting); Writing‐review & editing (equal). **Martin Solan:** Conceptualization (supporting); Funding acquisition (lead); Supervision (lead); Validation (equal); Writing‐original draft (supporting); Writing‐review & editing (lead). **Laura J Grange:** Conceptualization (equal); Formal analysis (supporting); Supervision (supporting); Writing‐review & editing (equal).

## Supporting information

Supplementary Material

## Data Availability

All data used to generate this manuscript are openly available via an unrestricted repository hosted by the UK Polar Data Centre (https://doi.org/10.5285/8976bd5c‐e91f‐4612‐880c‐7d15aca12809).
