# Supplementary material for: Reproductive traits and population dynamics of benthic invertebrates indicate episodic recruitment patterns across an Arctic polar front
Source: Ecol Evol. 2021 May 2;11(11):6900–12. doi: 10.1002/ece3.7539 (PMC8207403; doi:10.1002/ece3.7539)
Supplement: Supplementary file 1 — Supplementary Material [file ECE3-11-6900-s001.docx]

Reproductive traits and population dynamics of benthic invertebrates indicate episodic recruitment patterns across an Arctic polar front

**Supplemental Information**

Adam J. Reed^1^, Jasmin A. Godbold^1^, Martin Solan^1^, Laura J. Grange^2^

^1.^ School of Ocean and Earth Science, National Oceanography Centre Southampton, University of Southampton, European Way, Southampton, SO14 3ZH

^2.^ School of Ocean Sciences, Bangor University, Bangor, Gwynedd, LL57 2DG

**ORCID ID**

AJR, 0000-0003-2200-5067; JAG, 0000-0001-5558-8188; MS, 0000-0001-9924-5574; LG, 0000-0001-9222-6848

Table S1

Table of station data from Agassiz trawls (AGT) on JR16006 research cruise to the Barents Sea in July 2017. Event number relates to the corresponding number within the cruise report (Hopkins 2018). Trawl tows were undertaken for 15 minutes each at a ship speed of 1 knot.

| **Station** | **Event Number** | **Date** | **Lat. °N on bottom** | **Long. °E off bottom** | **Depth (m)** | **No. *Astarte crenata*** | **No. *Ctenodiscus crispatus*** |
| --- | --- | --- | --- | --- | --- | --- | --- |
| B13 | 133 | 17/07/17 | 74.498 | 30.004 | 360 | 9 | 44 |
|  | 135 | 17/07/17 | 74.500 | 30.003 | 359 | 24 | 38 |
|  | 136 | 17/07/17 | 74.492 | 30.003 | 360 | 10 | 20 |
|  | 137 | 17/07/17 | 74.499 | 29.997 | 363 | 18 | 29 |
| B14 | 304 | 30/07/17 | 76.502 | 30.504 | 293 | / | 78 |
|  | 305 | 30/07/17 | 76.502 | 30.499 | 296 | / | 40 |
|  | 306 | 30/07/17 | 76.500 | 30.498 | 296 | / | 9 |
| B16 | 216 | 23/07/17 | 80.058 | 30.037 | 285 | 33 | 34 |
|  | 217 | 23/07/17 | 80.063 | 30.032 | 286 | 43 | 21 |
|  | 218 | 23/07/17 | 80.066 | 30.061 | 280 | 22 | 11 |

Figure S1.

Study area indicating the position of stations B13 - B17 in the Barents Sea and the bathymetry relative to chart datum.


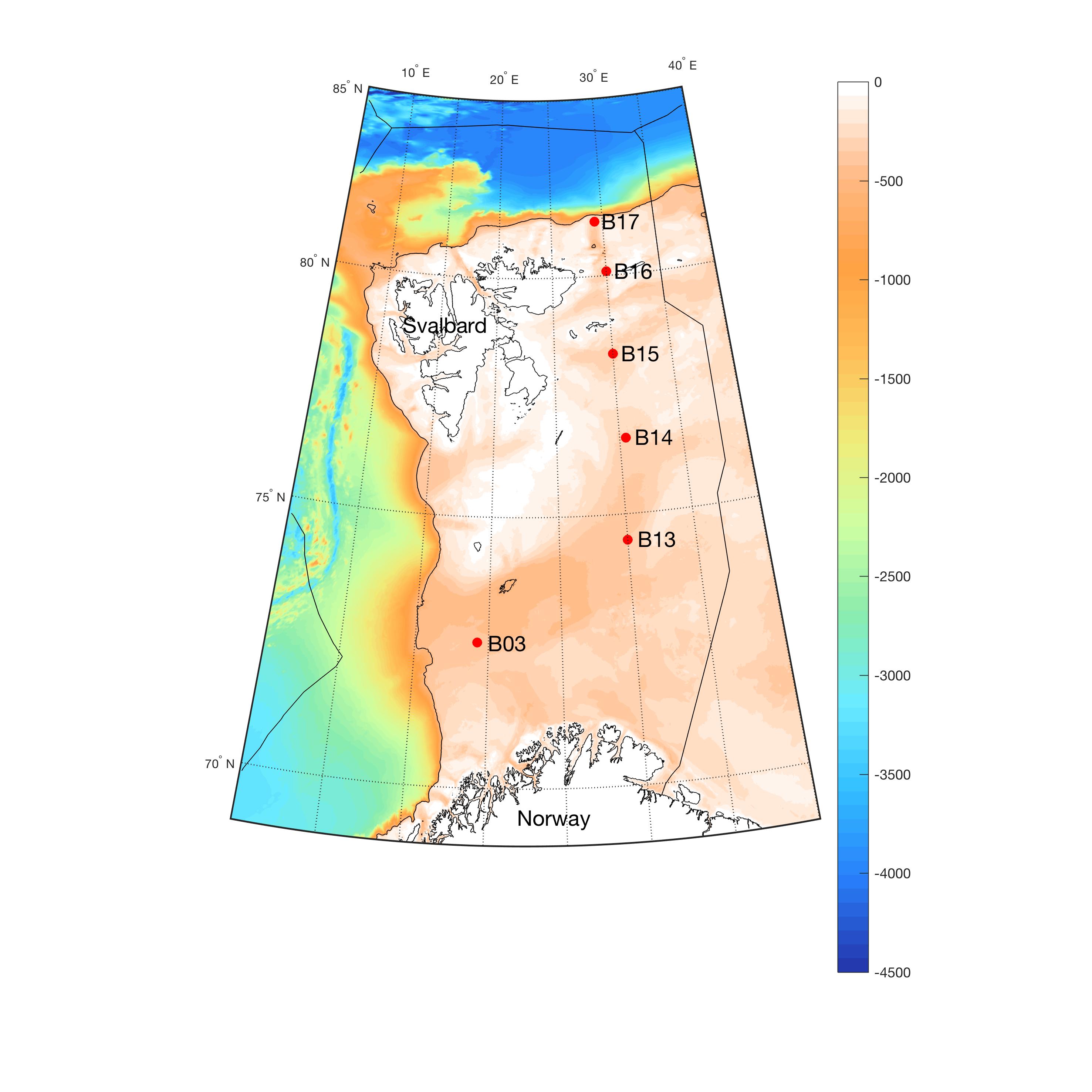


Figure S2.

The global distribution of (a) *Astarte crenata* (778 records) and (b) *Ctenodiscus crispatus* (2107 records) based on records held in OBIS, the Ocean Biogeographic Information System (<https://obis.org/>, accessed 30/01/2020; Grassle, 2000).

(b)

| 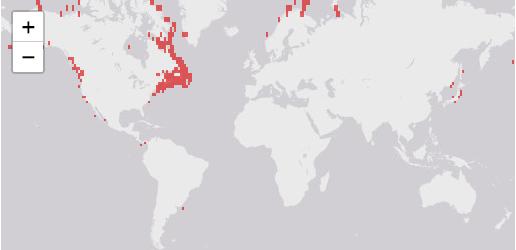 |  | 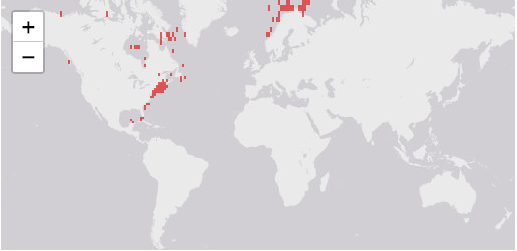 |
| --- | --- | --- |
|  |  |  |


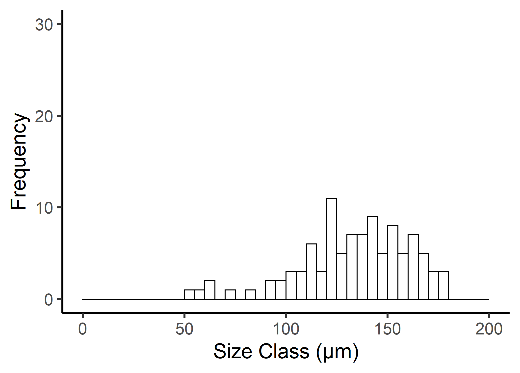


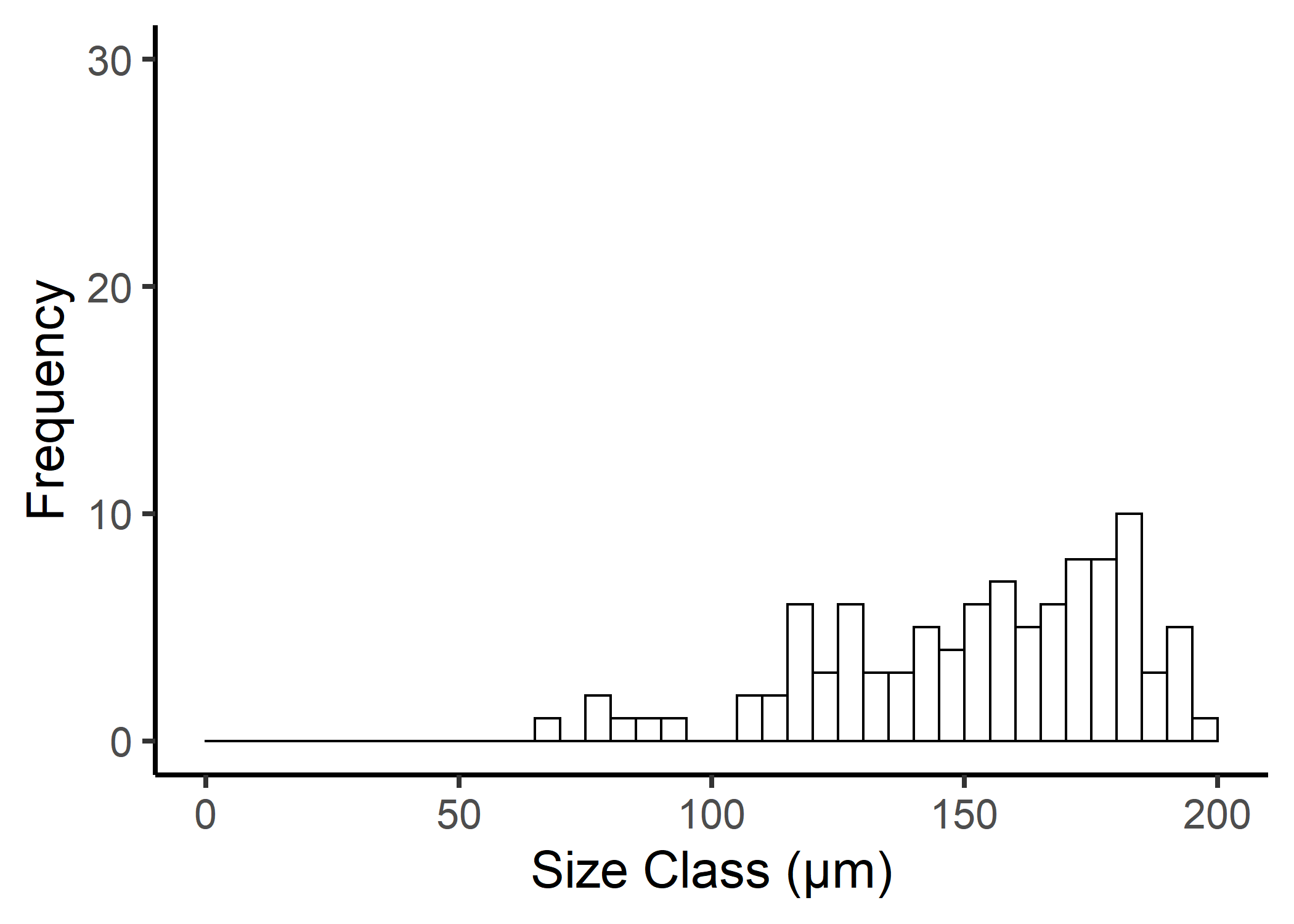

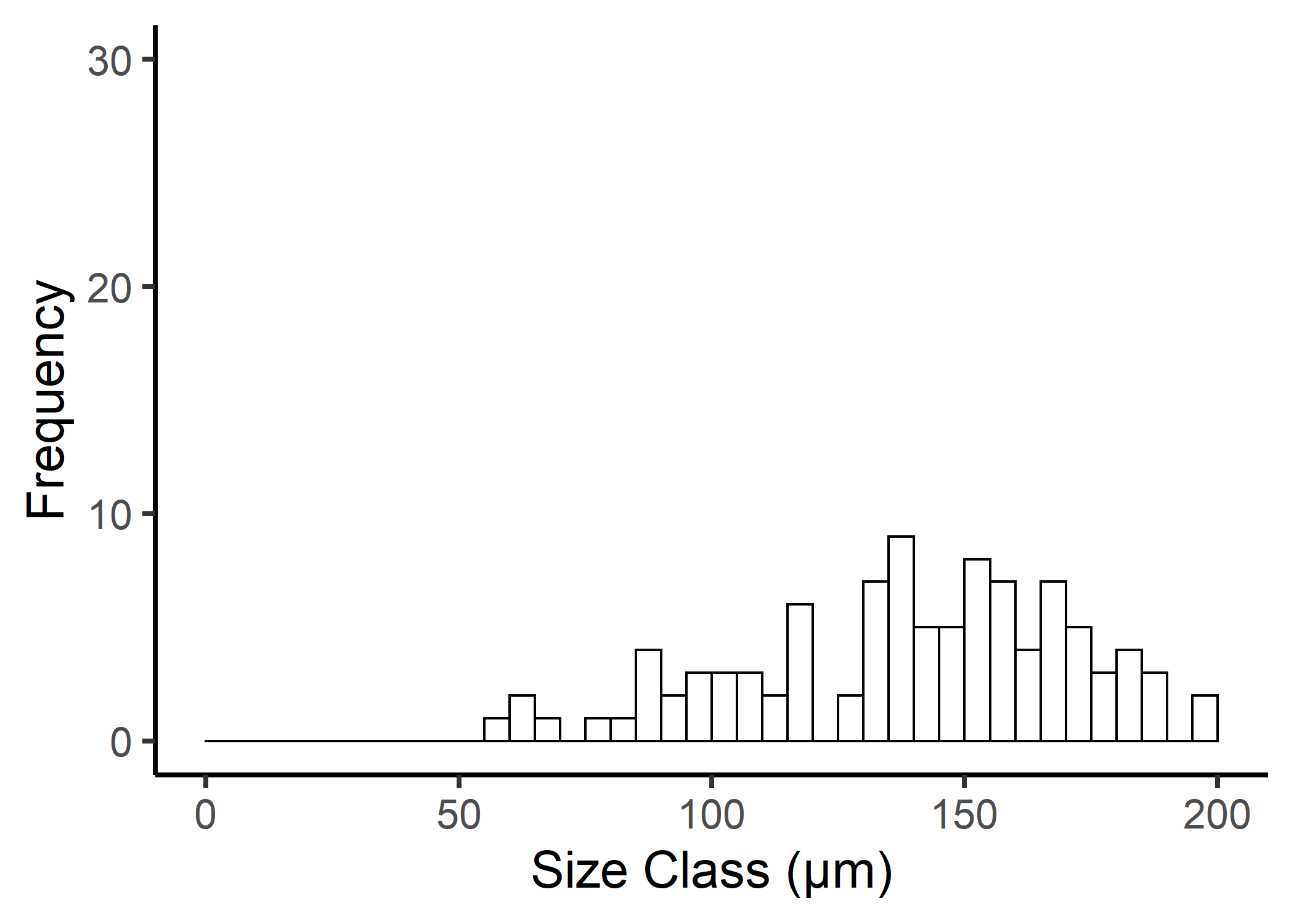

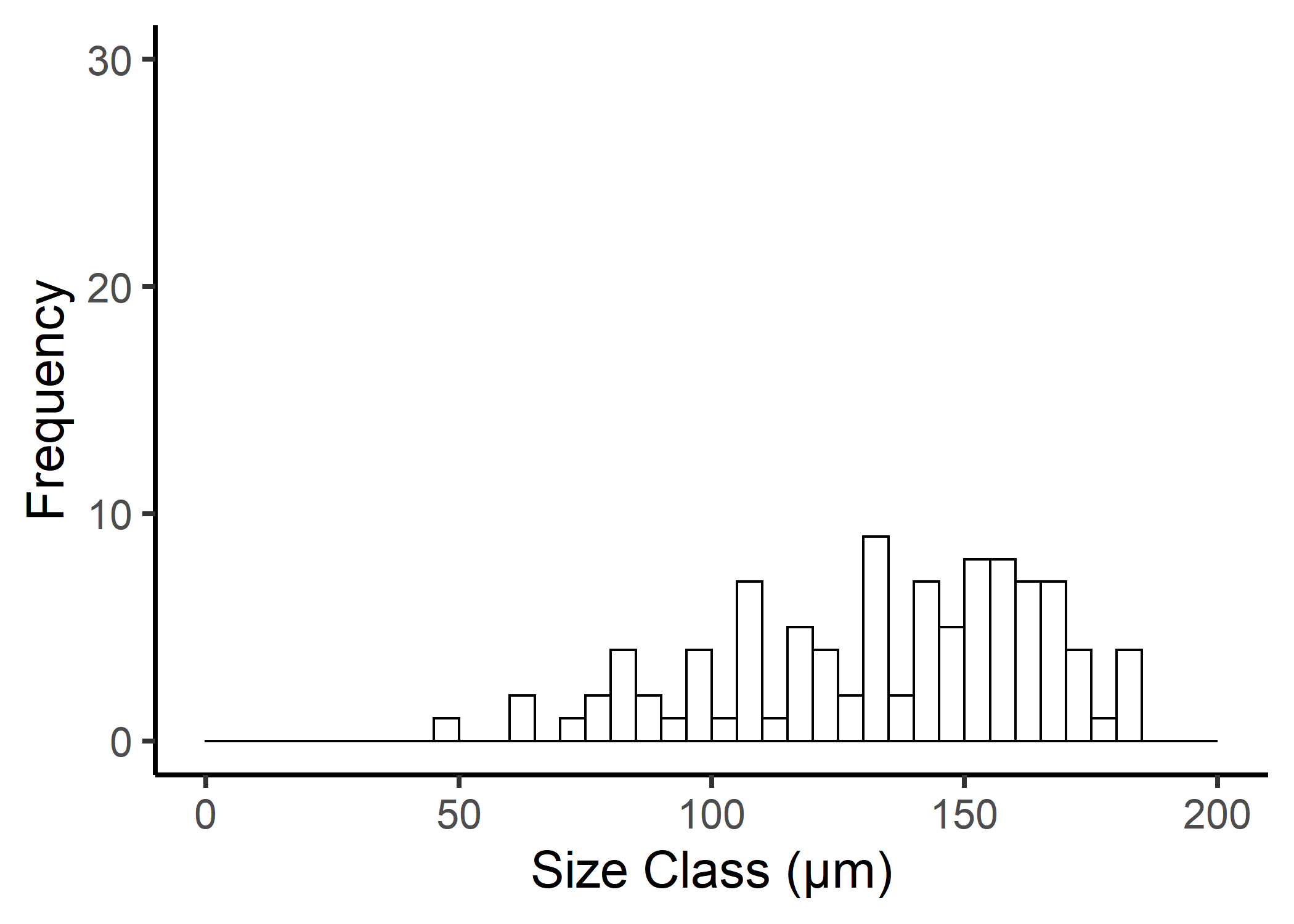

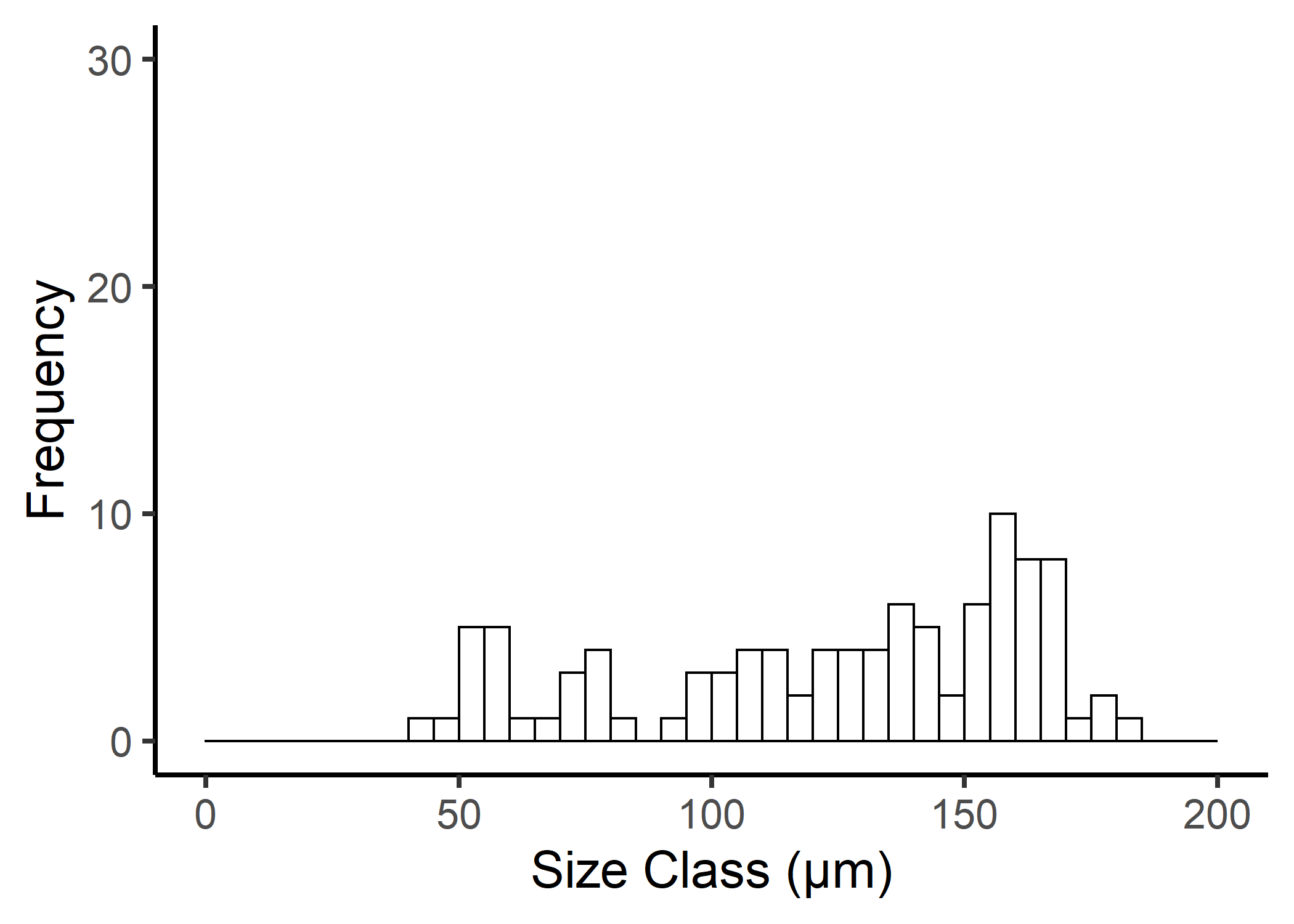

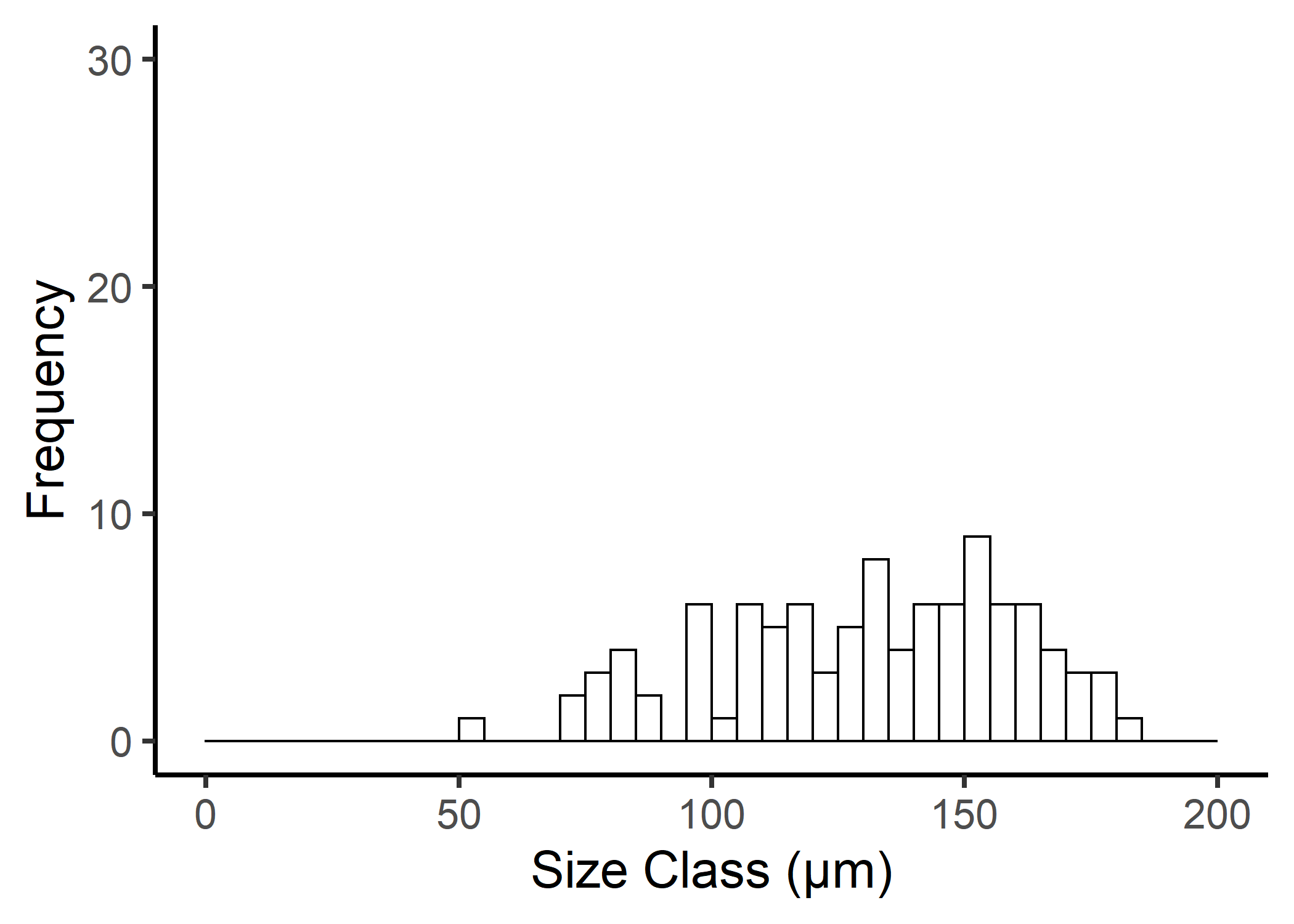

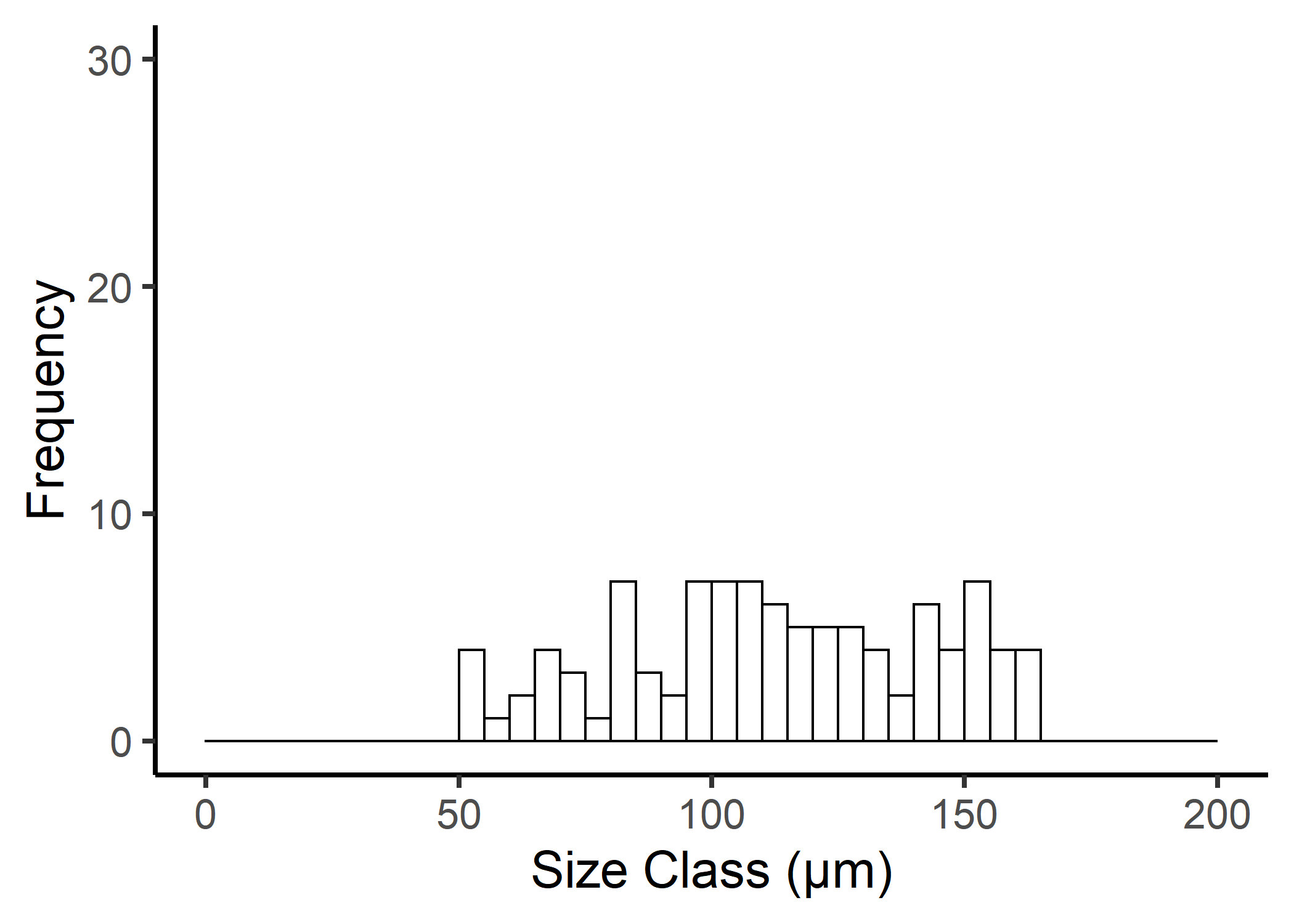

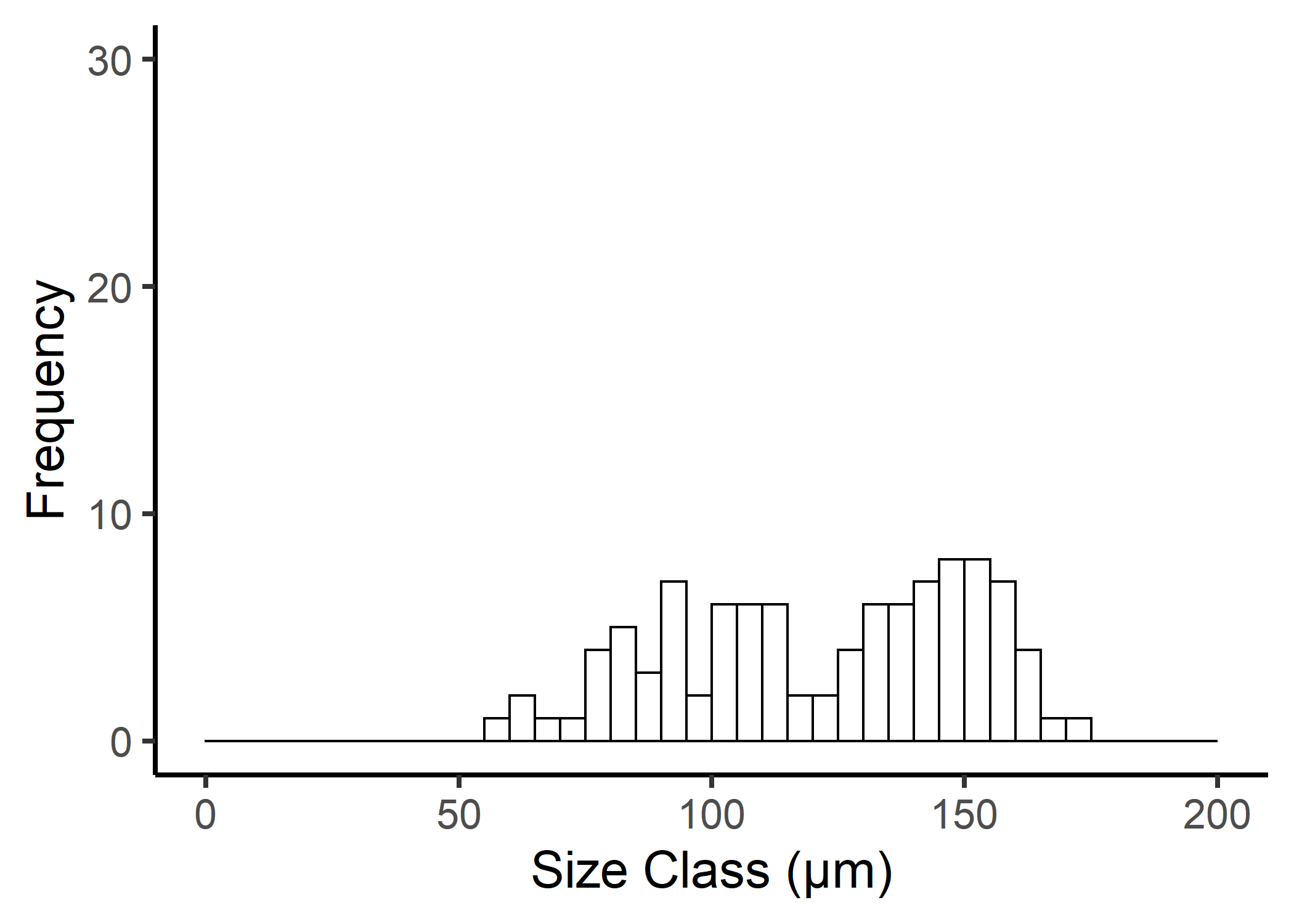

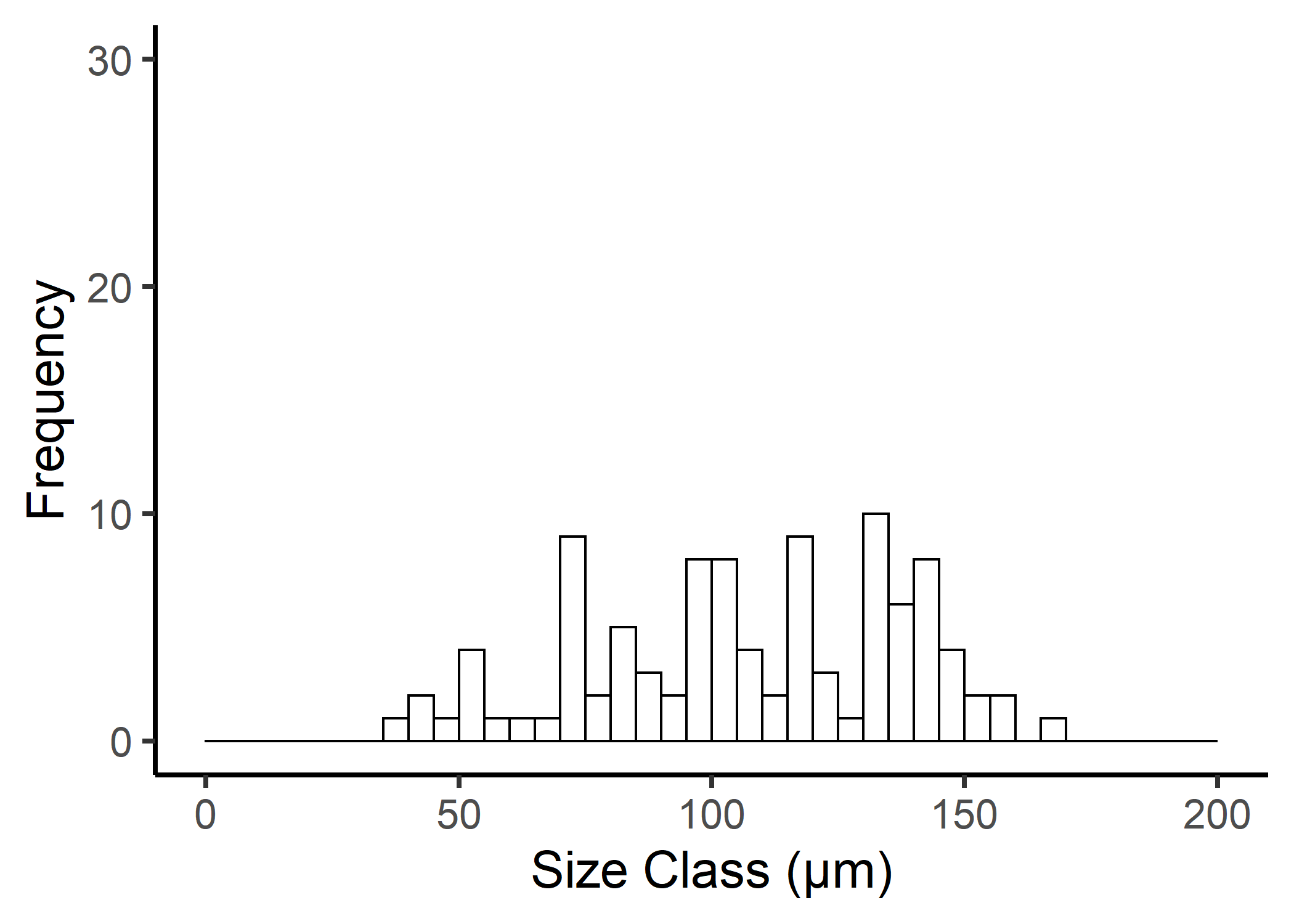

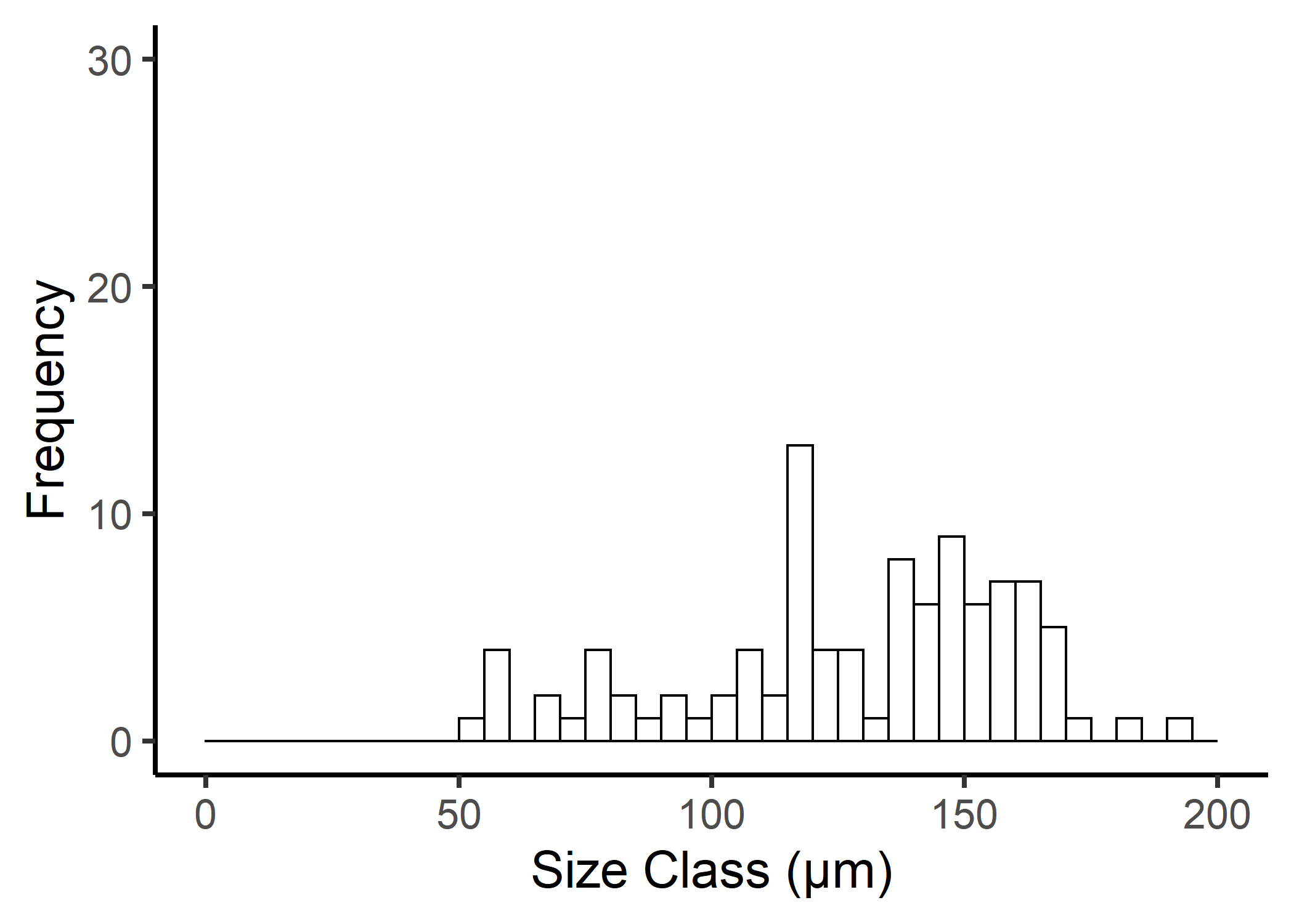

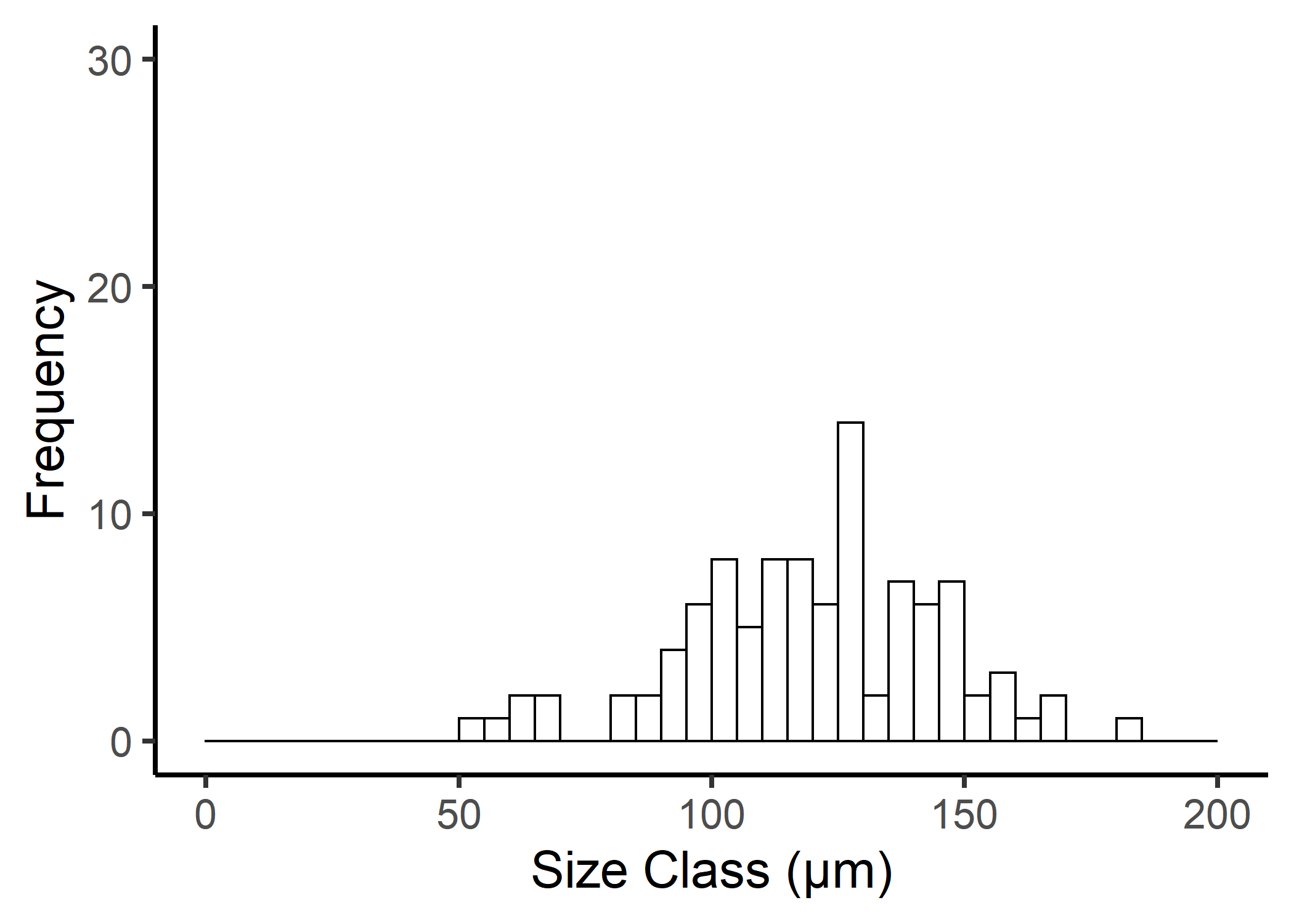

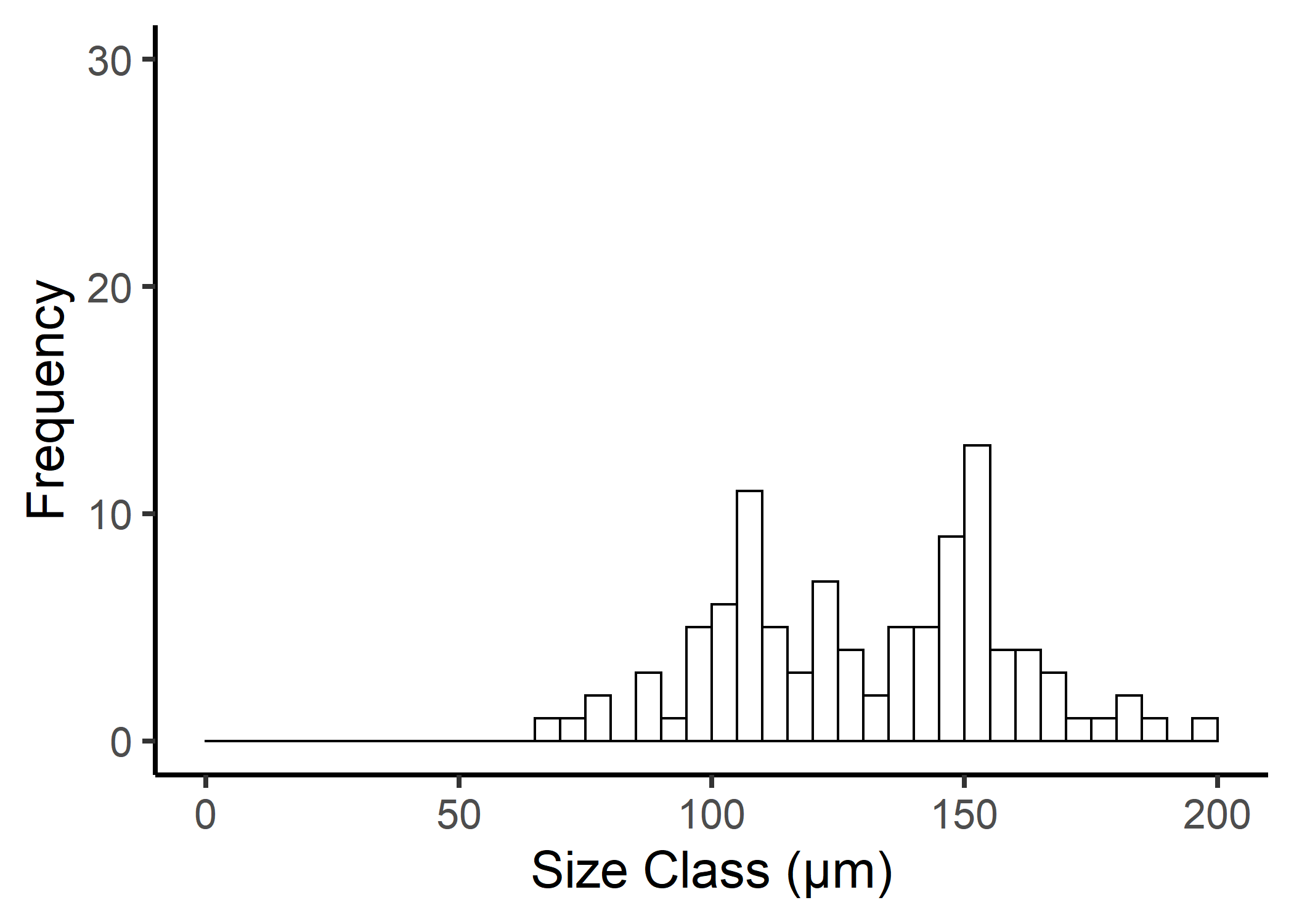


Figure S3. *Astarte crenata* individual oocyte size frequencies at station B13


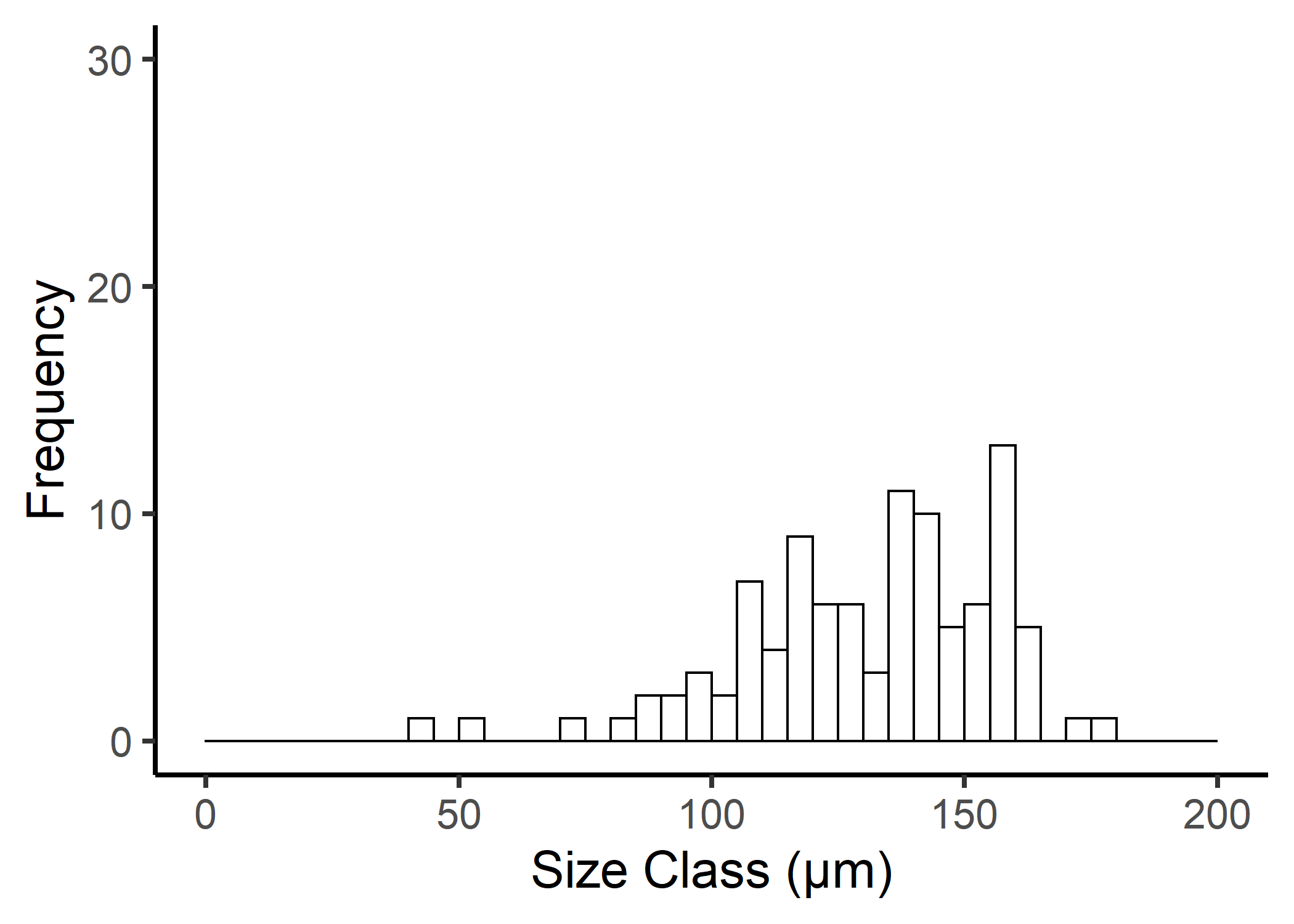

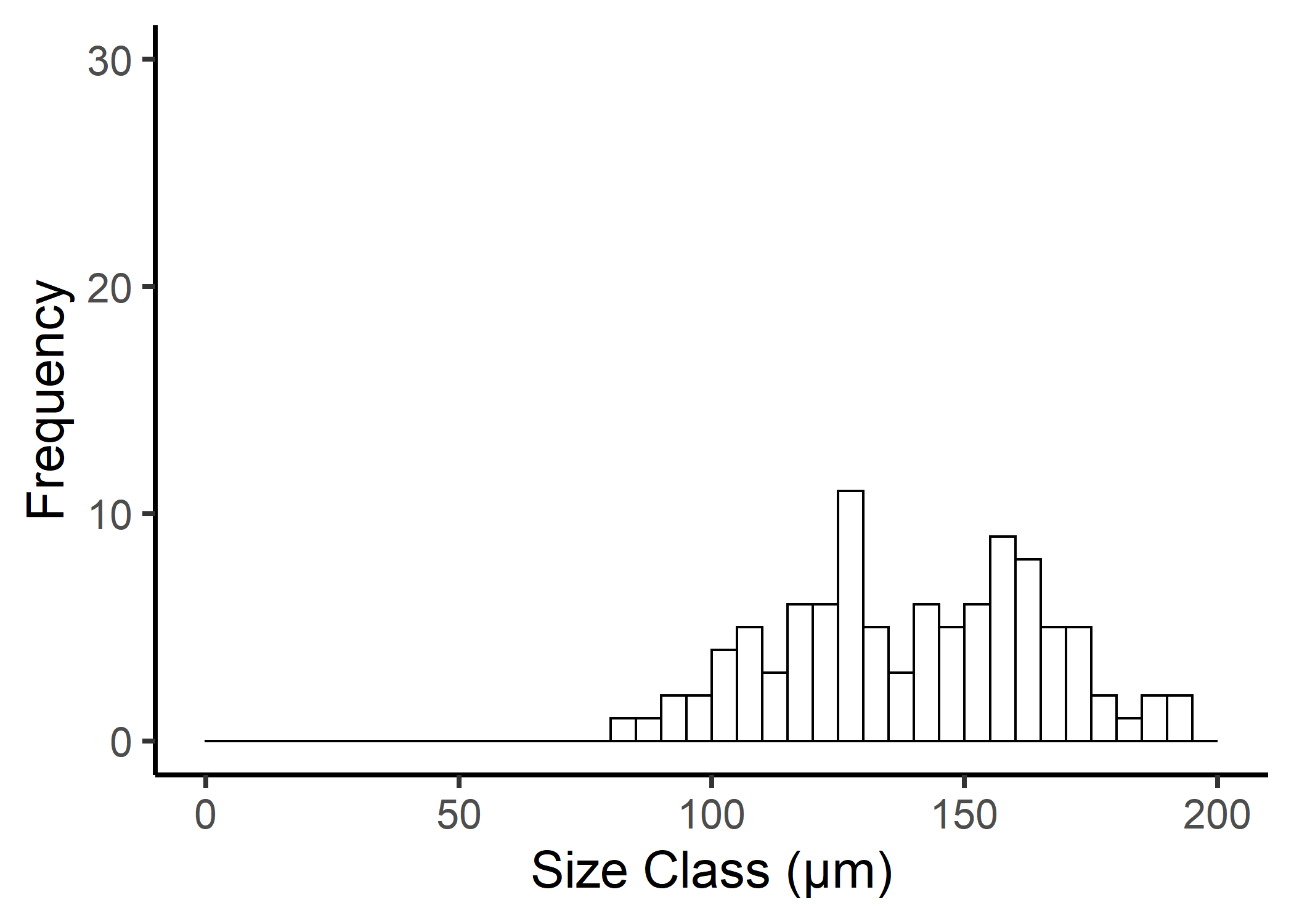

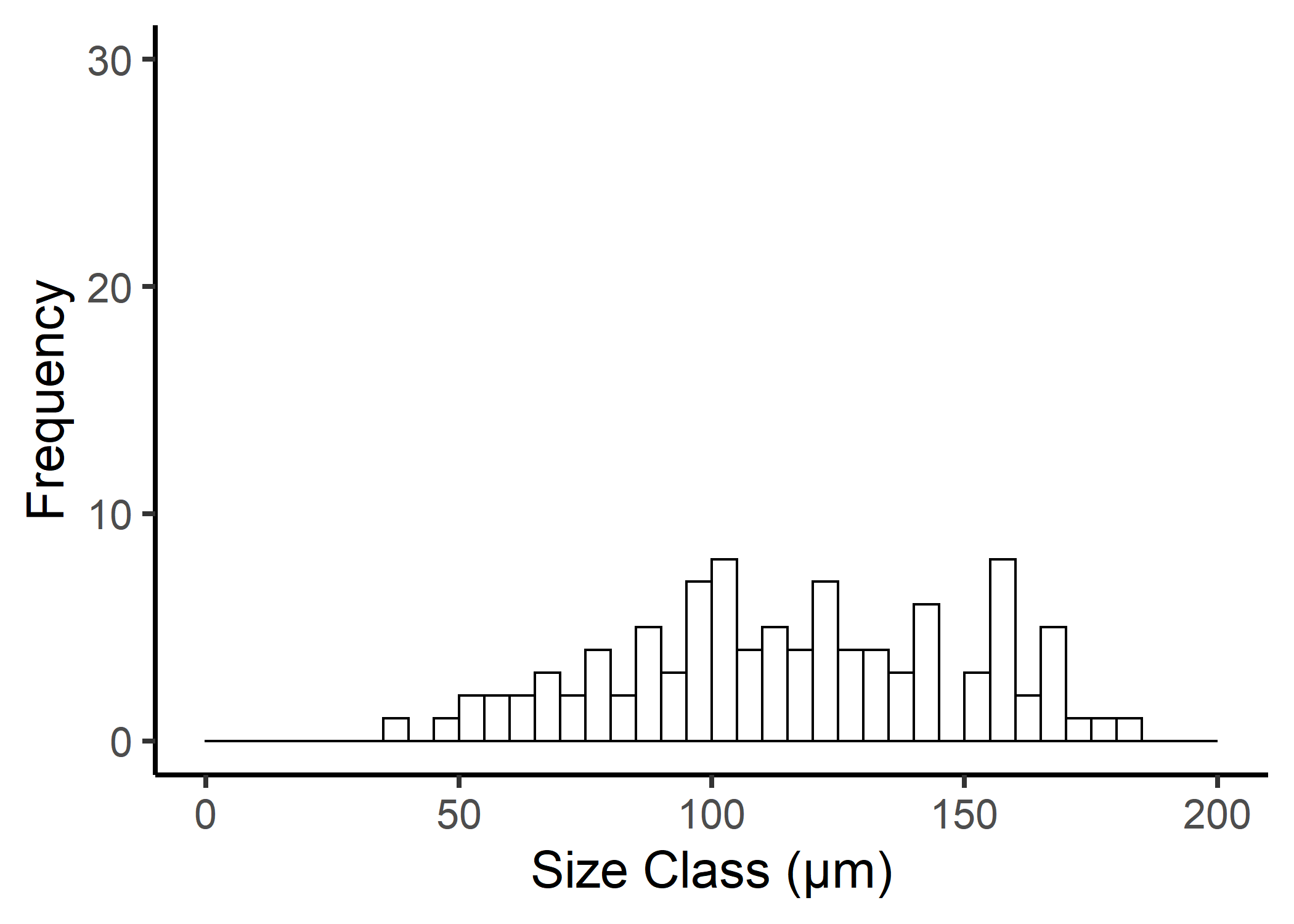

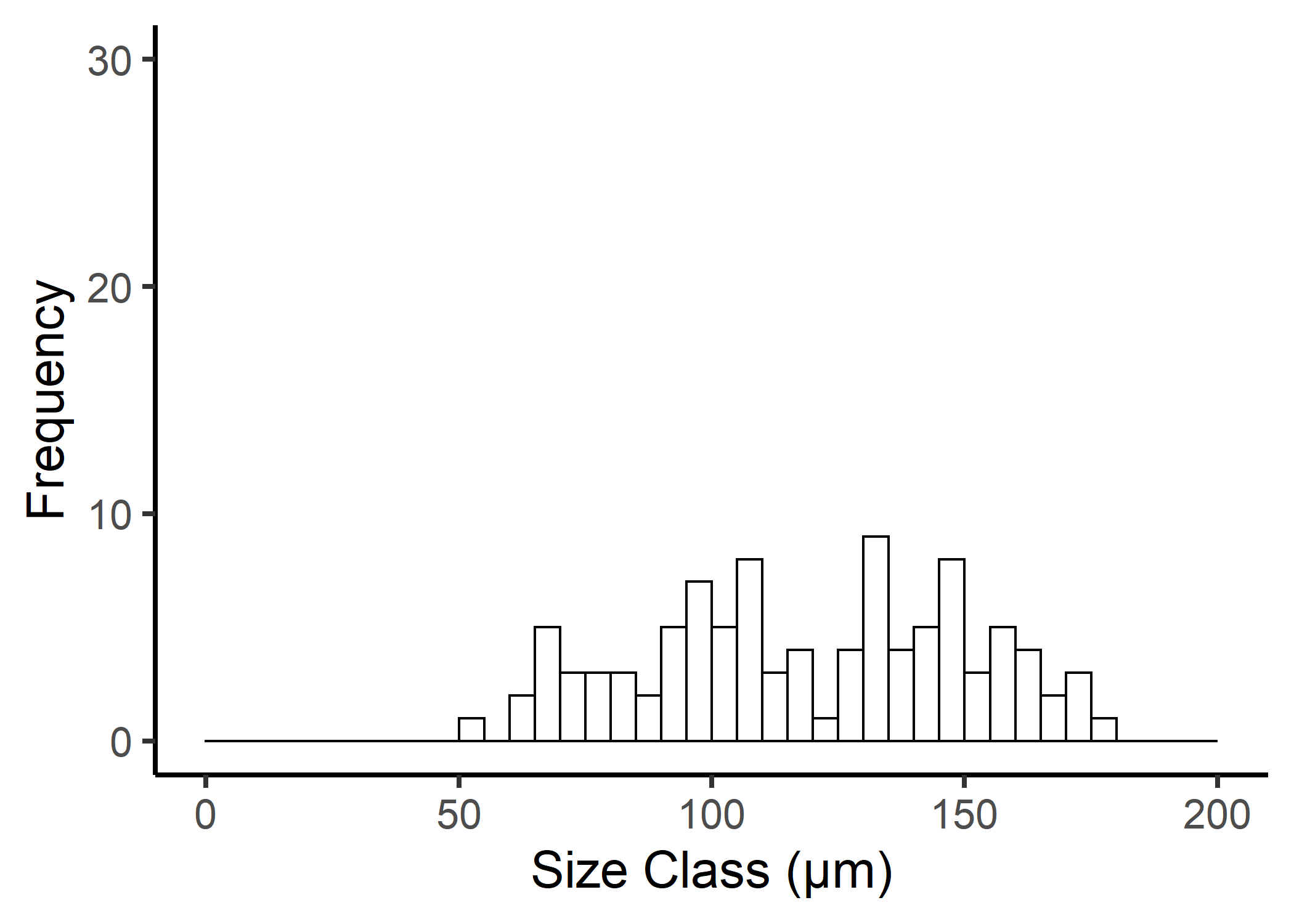

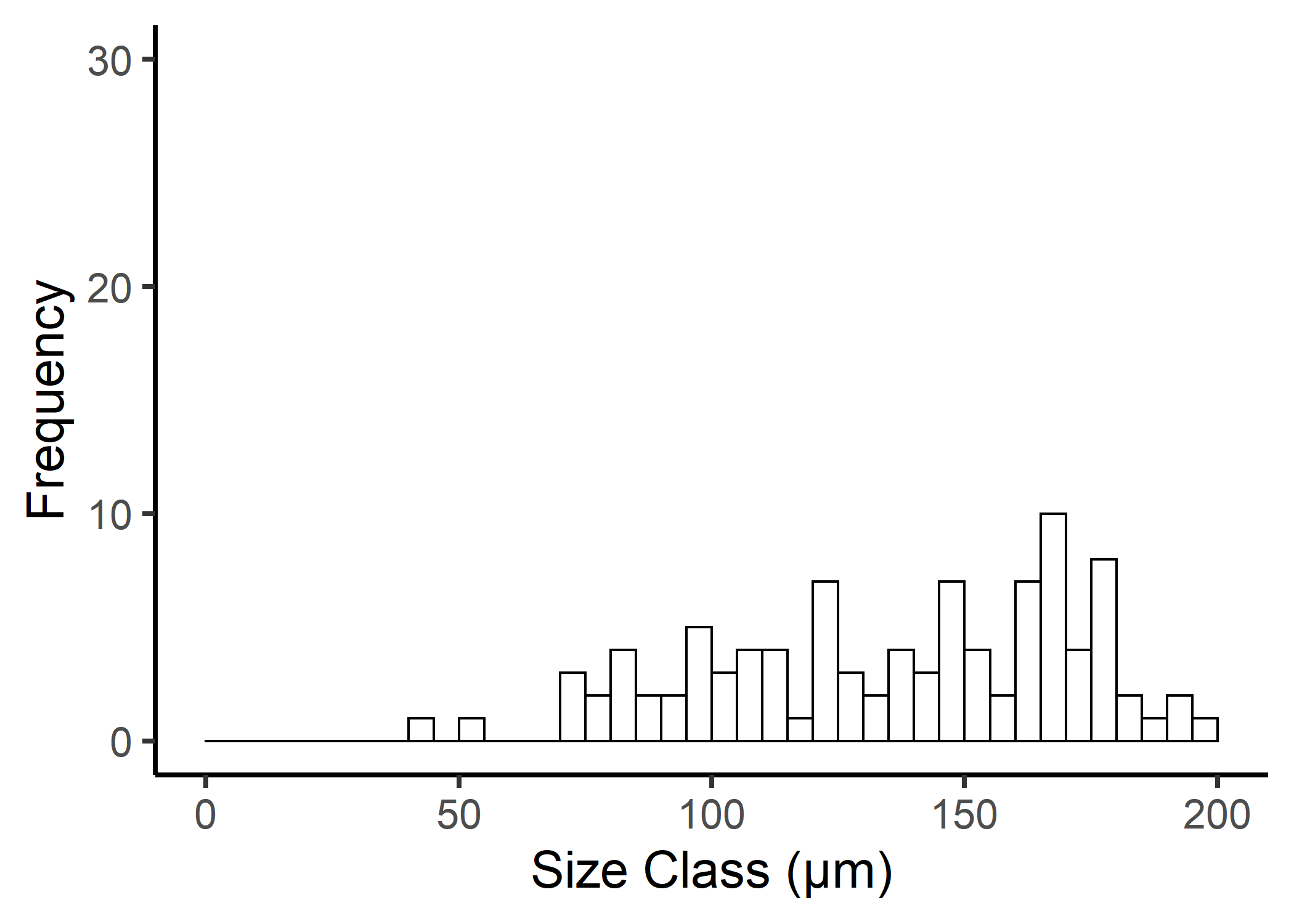

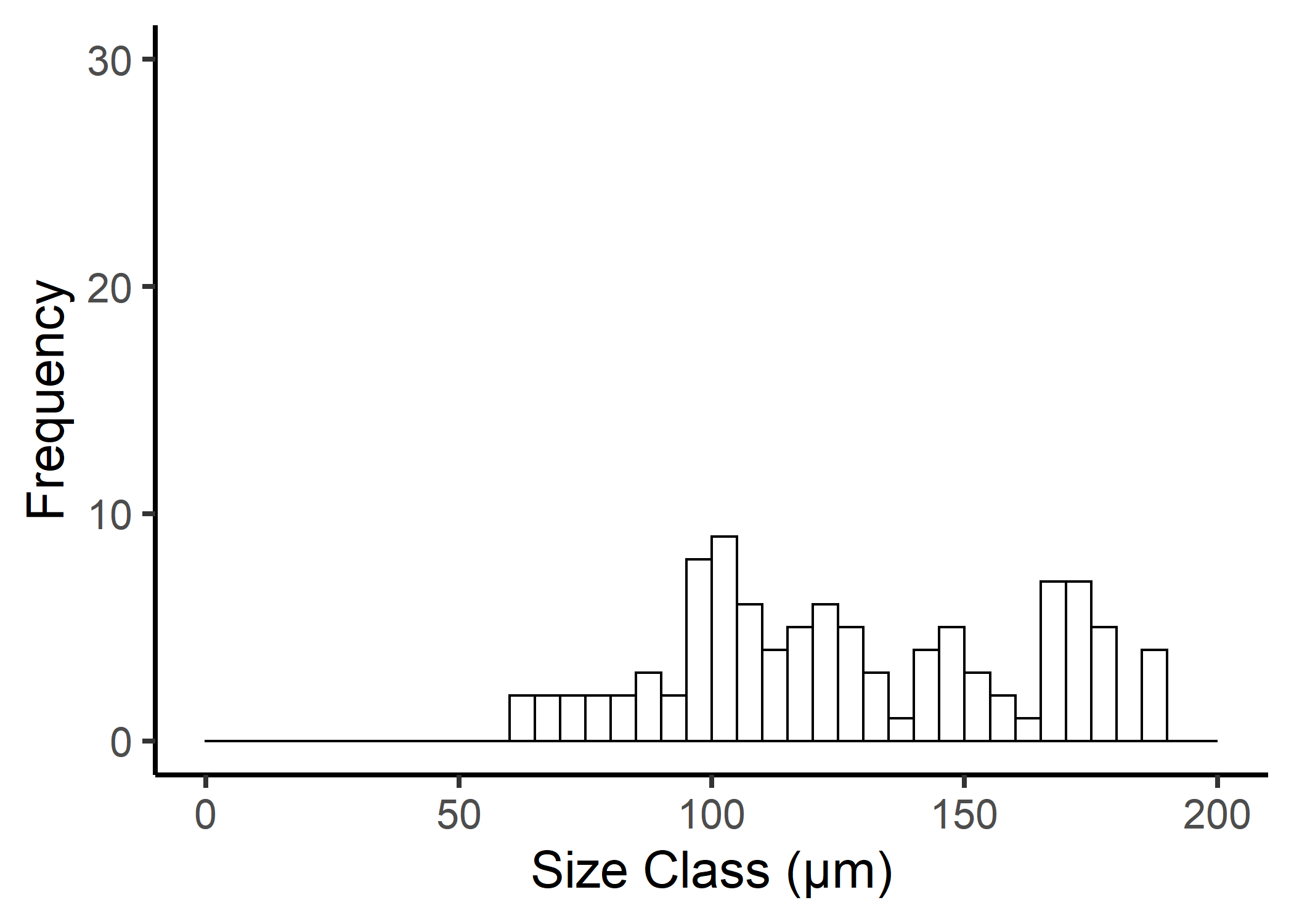

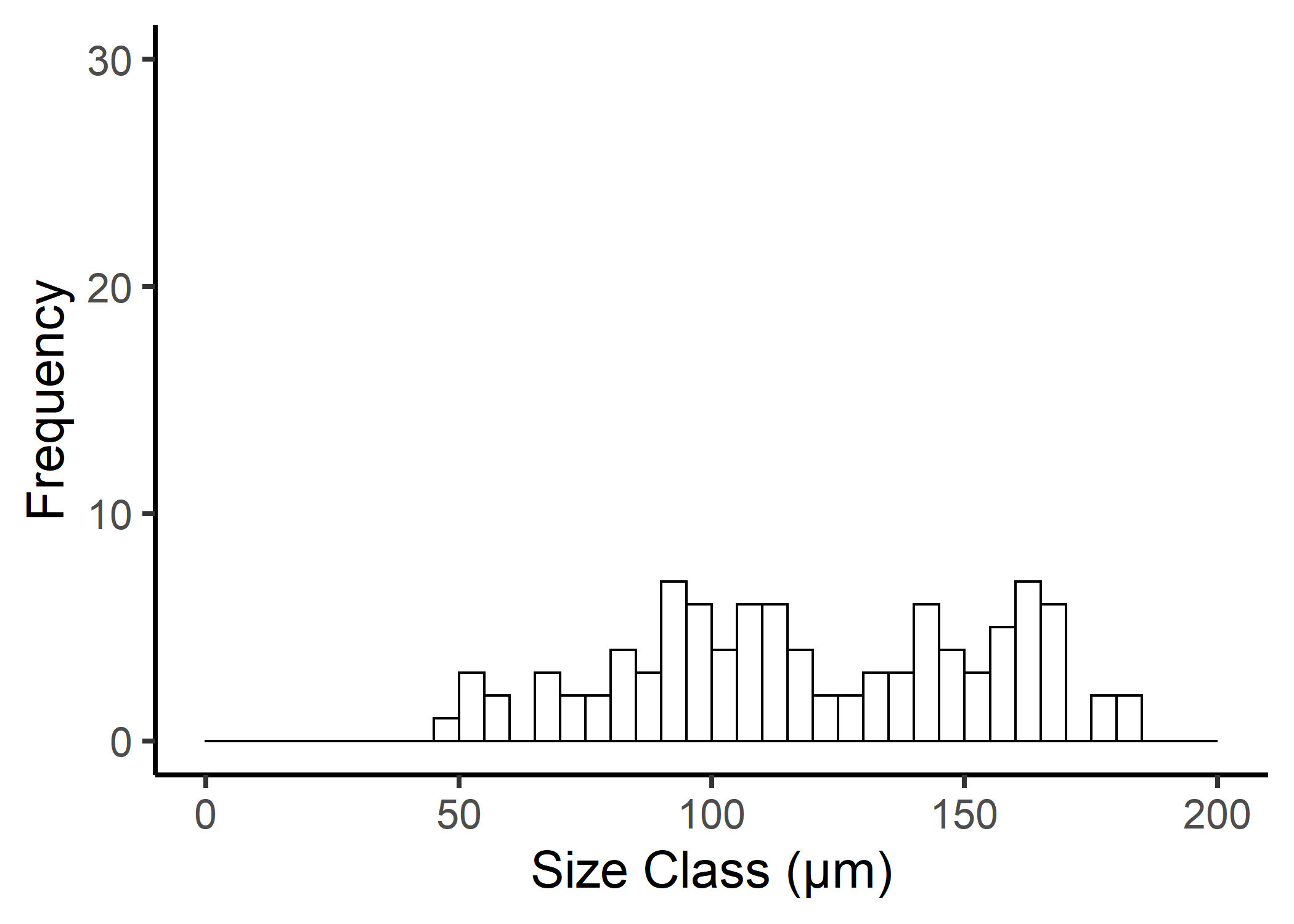

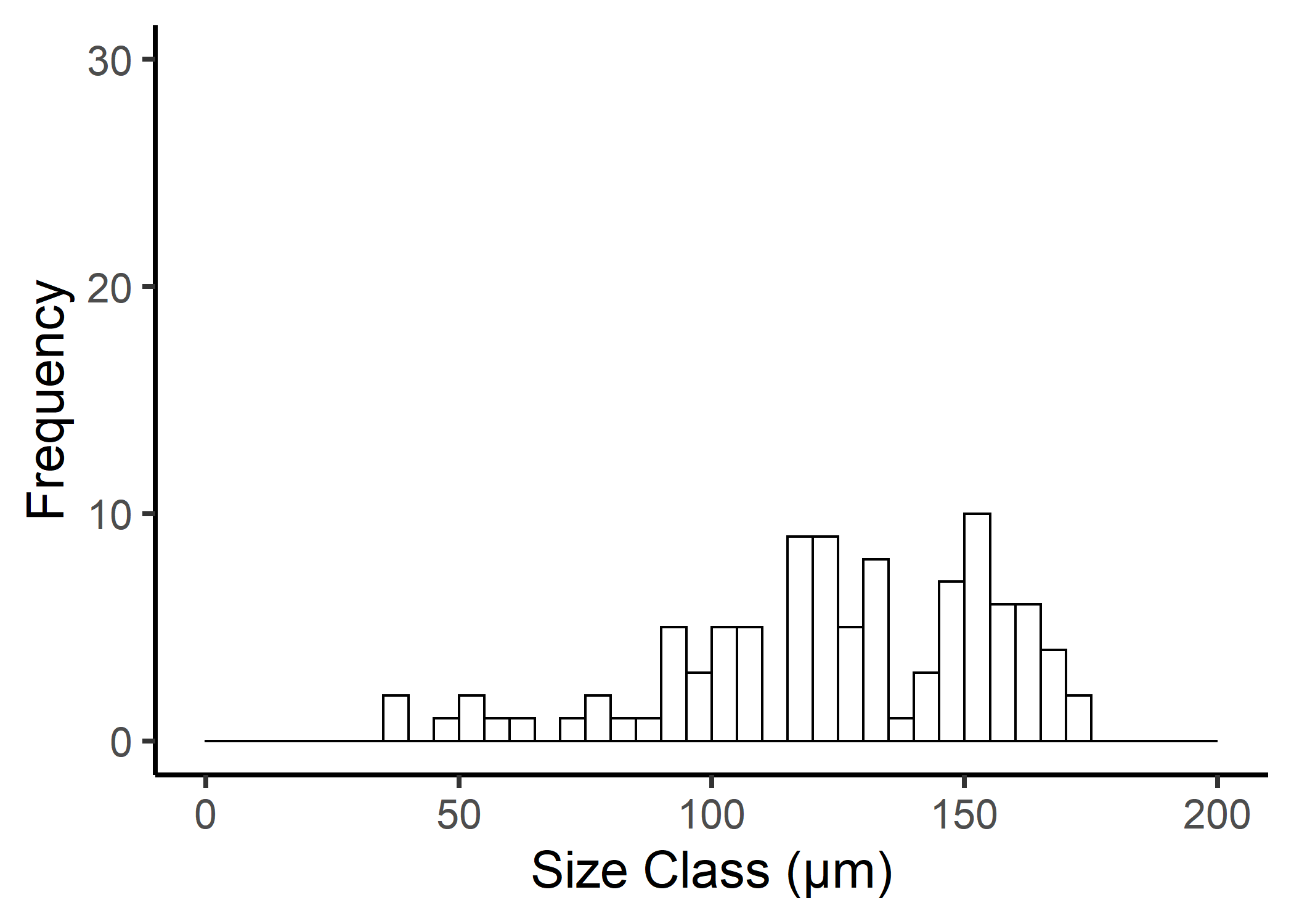

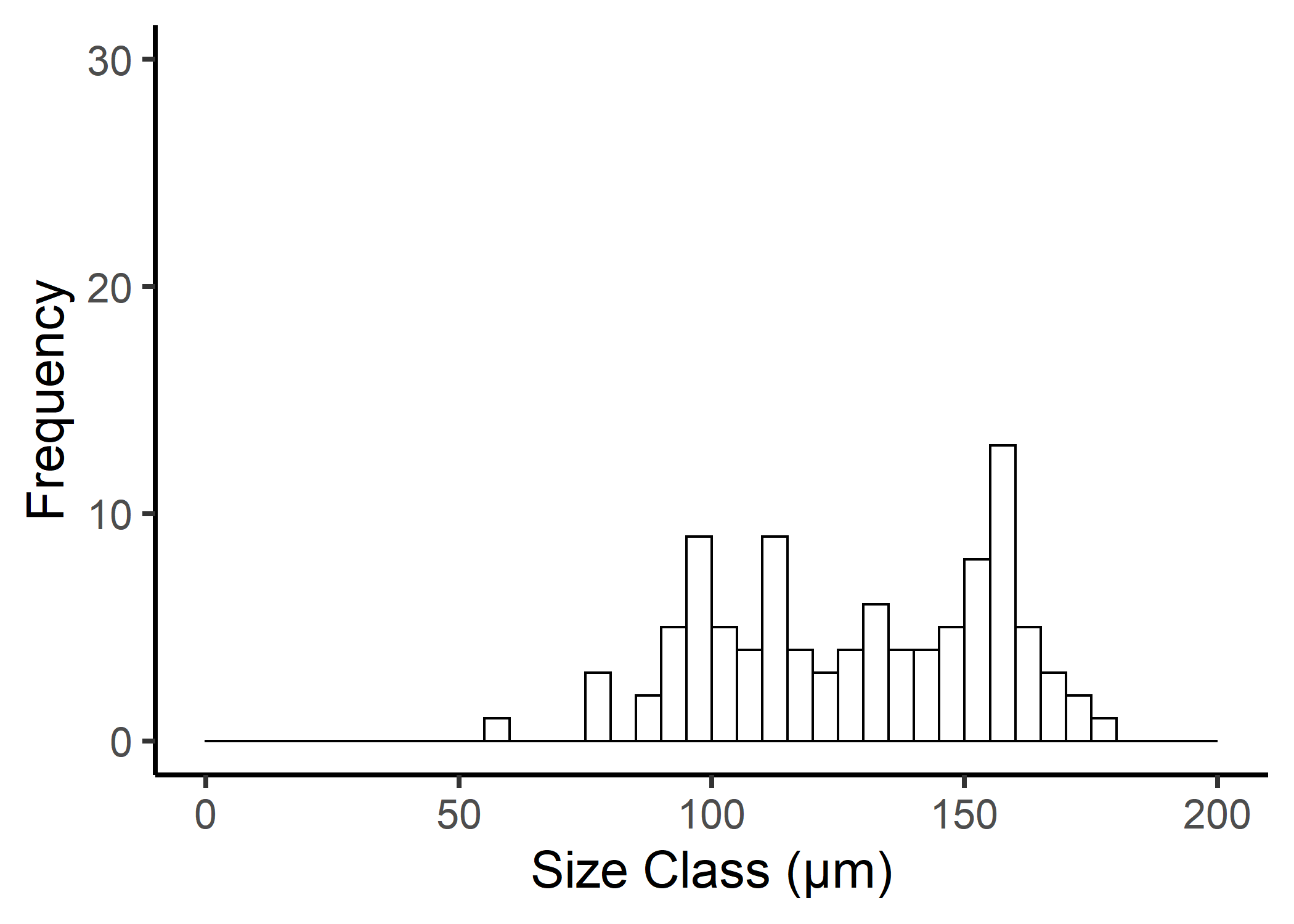

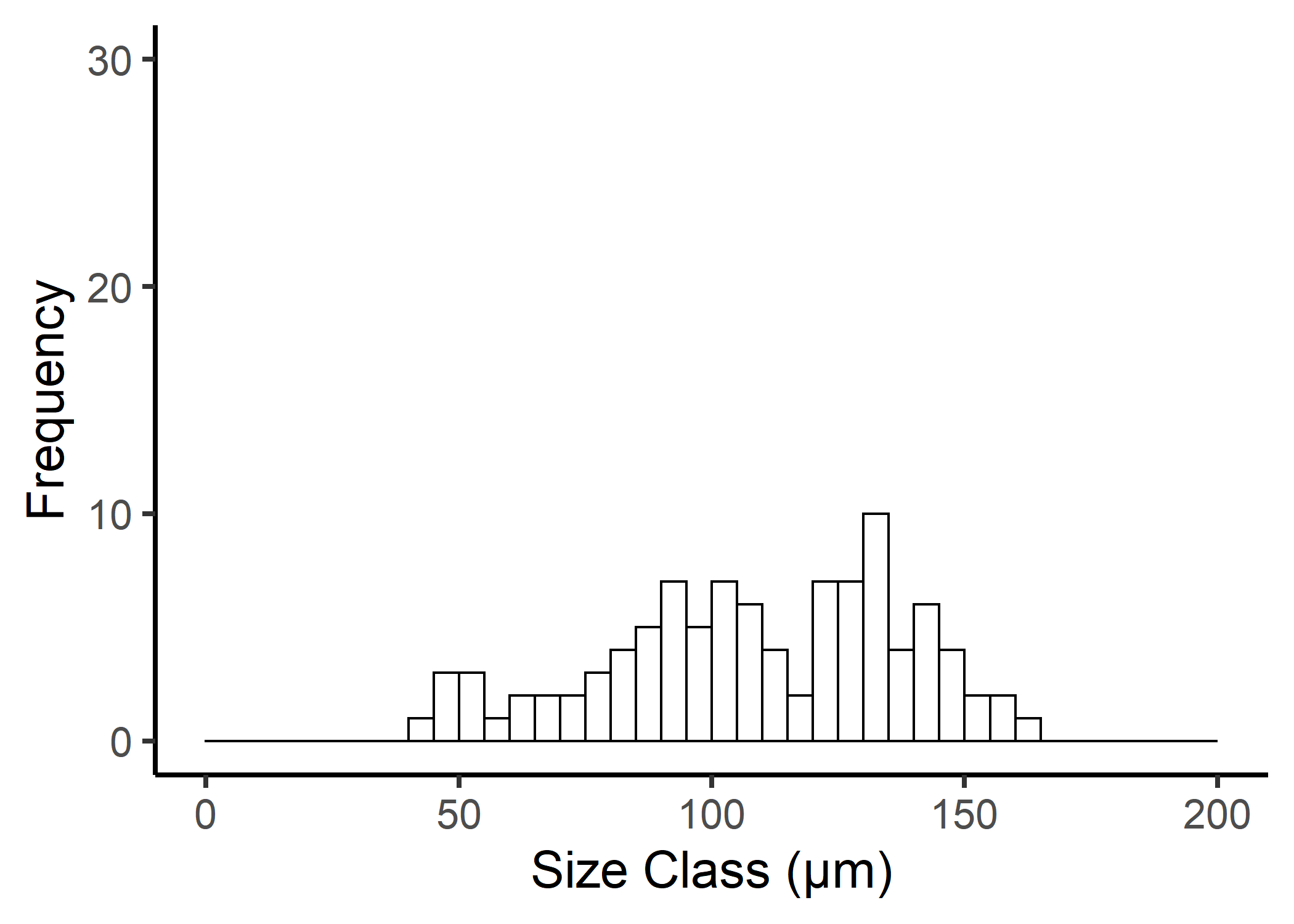

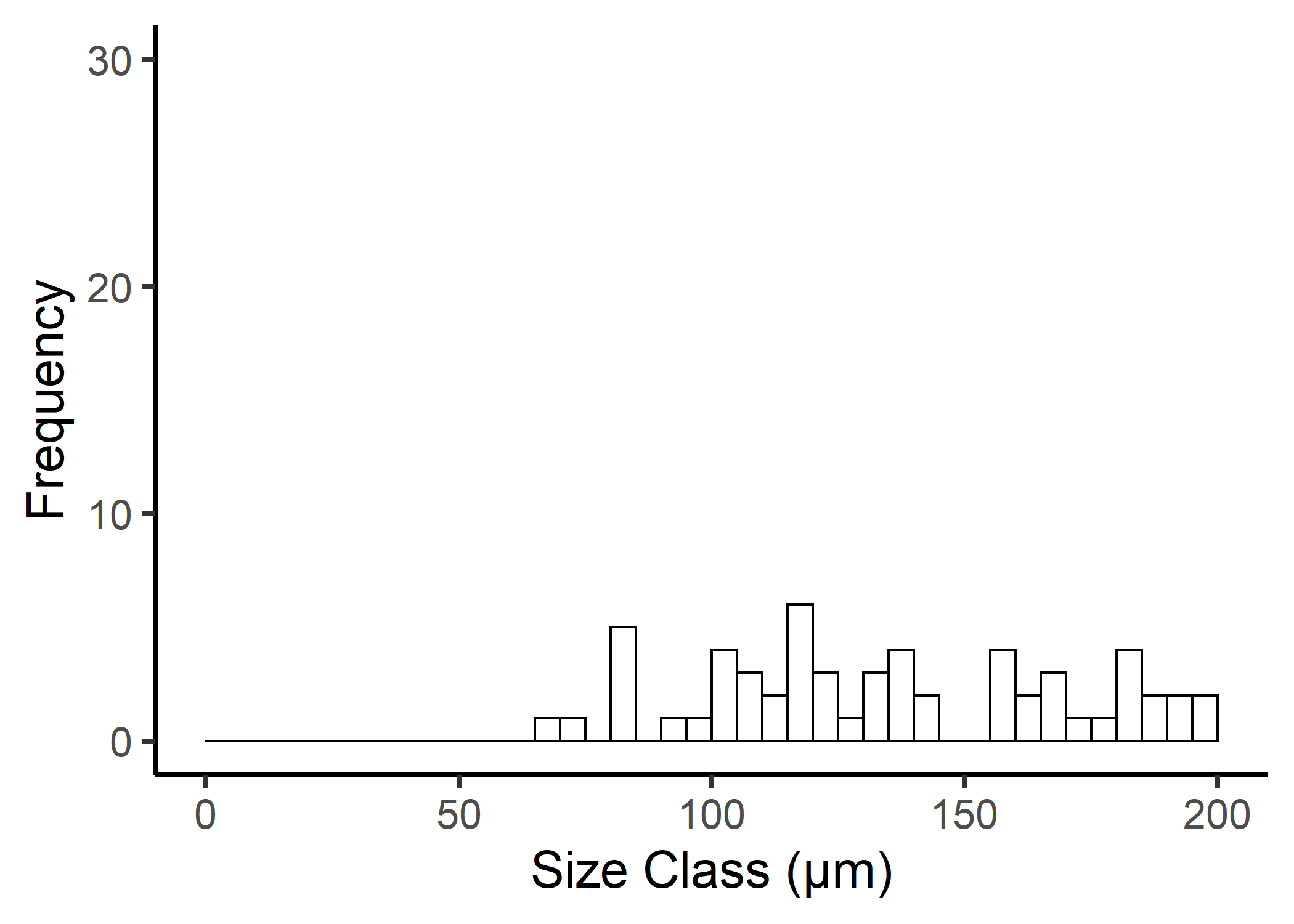

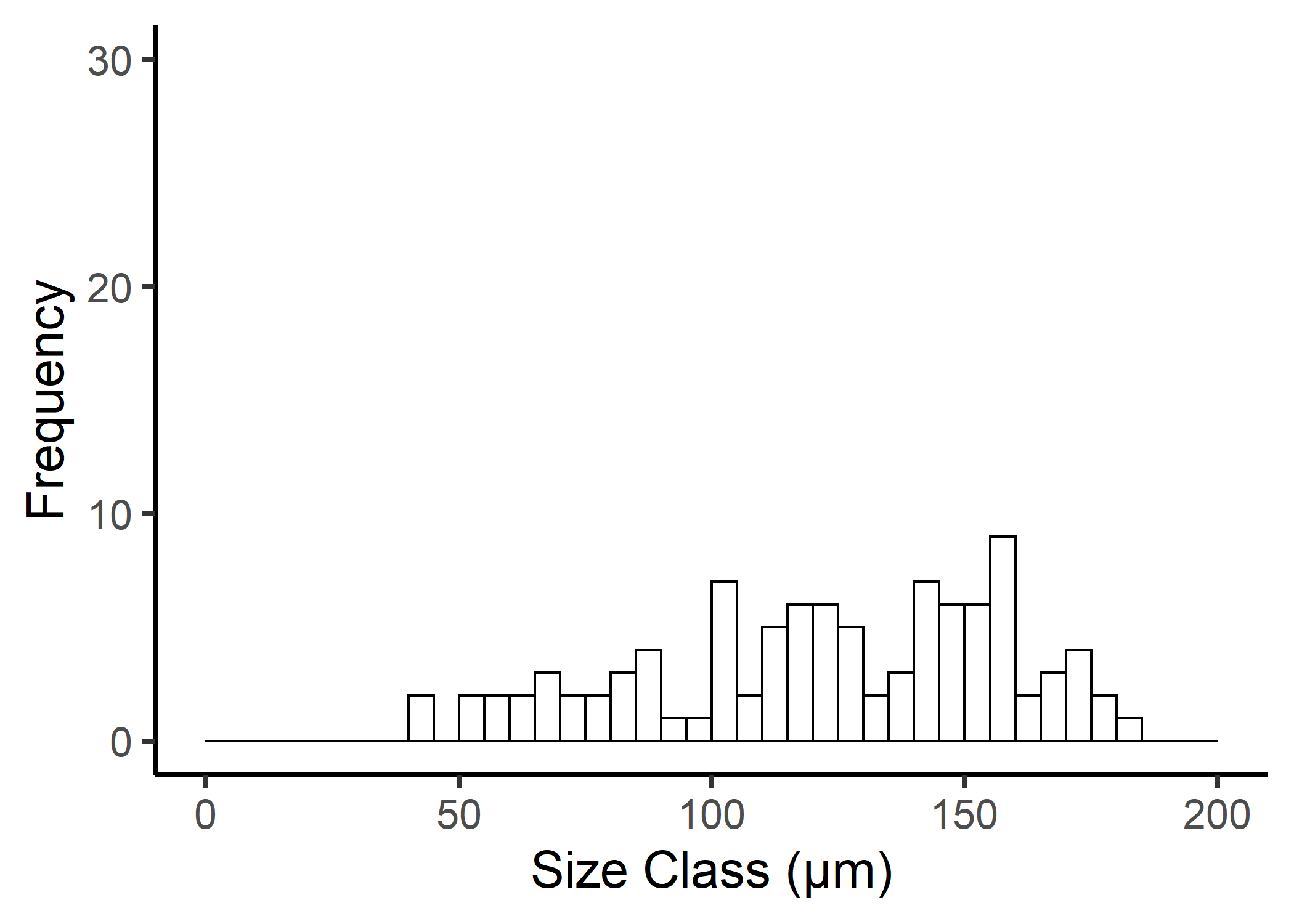


Figure S4. *Astarte crenata* individual oocyte size frequencies at station B16


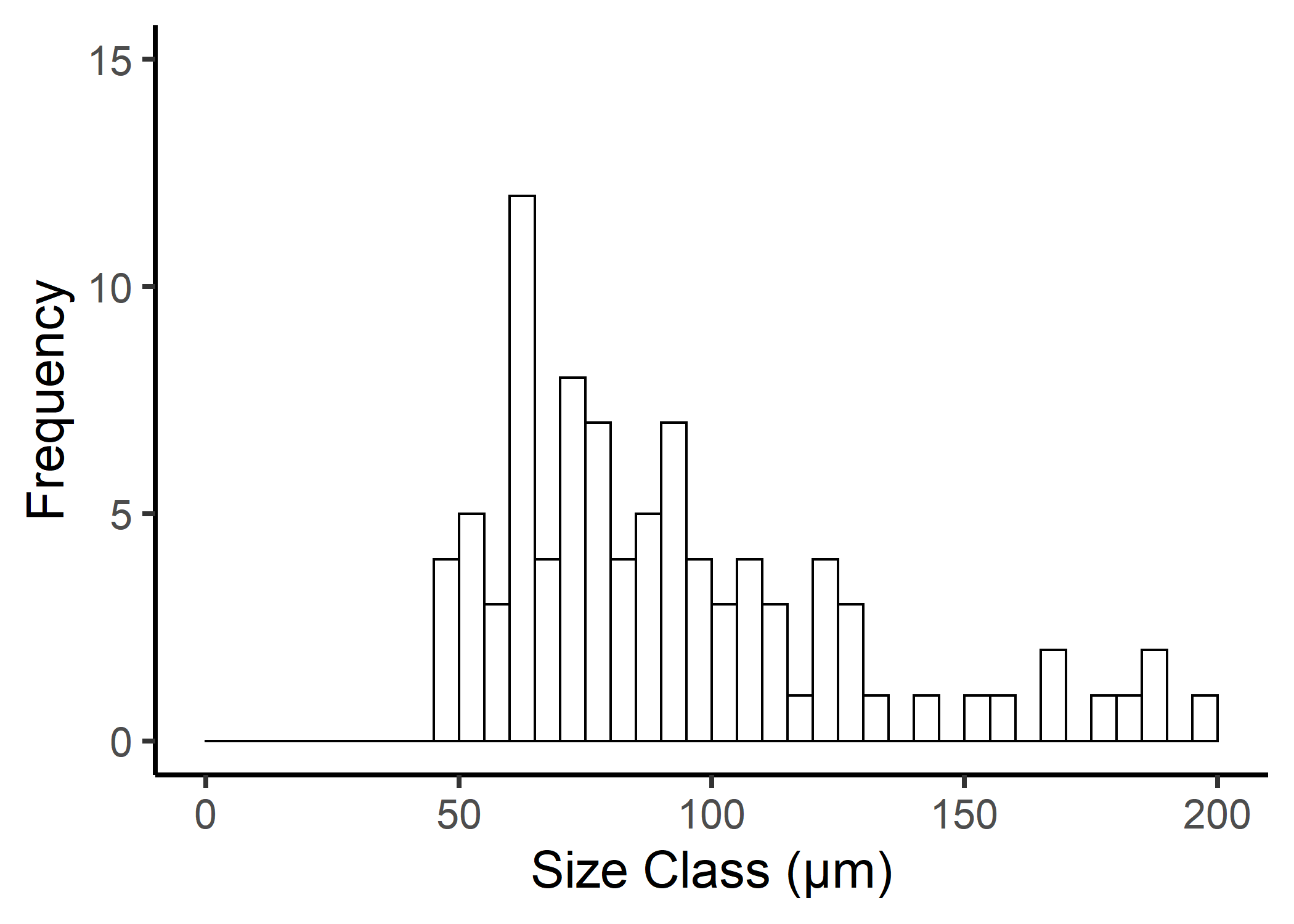

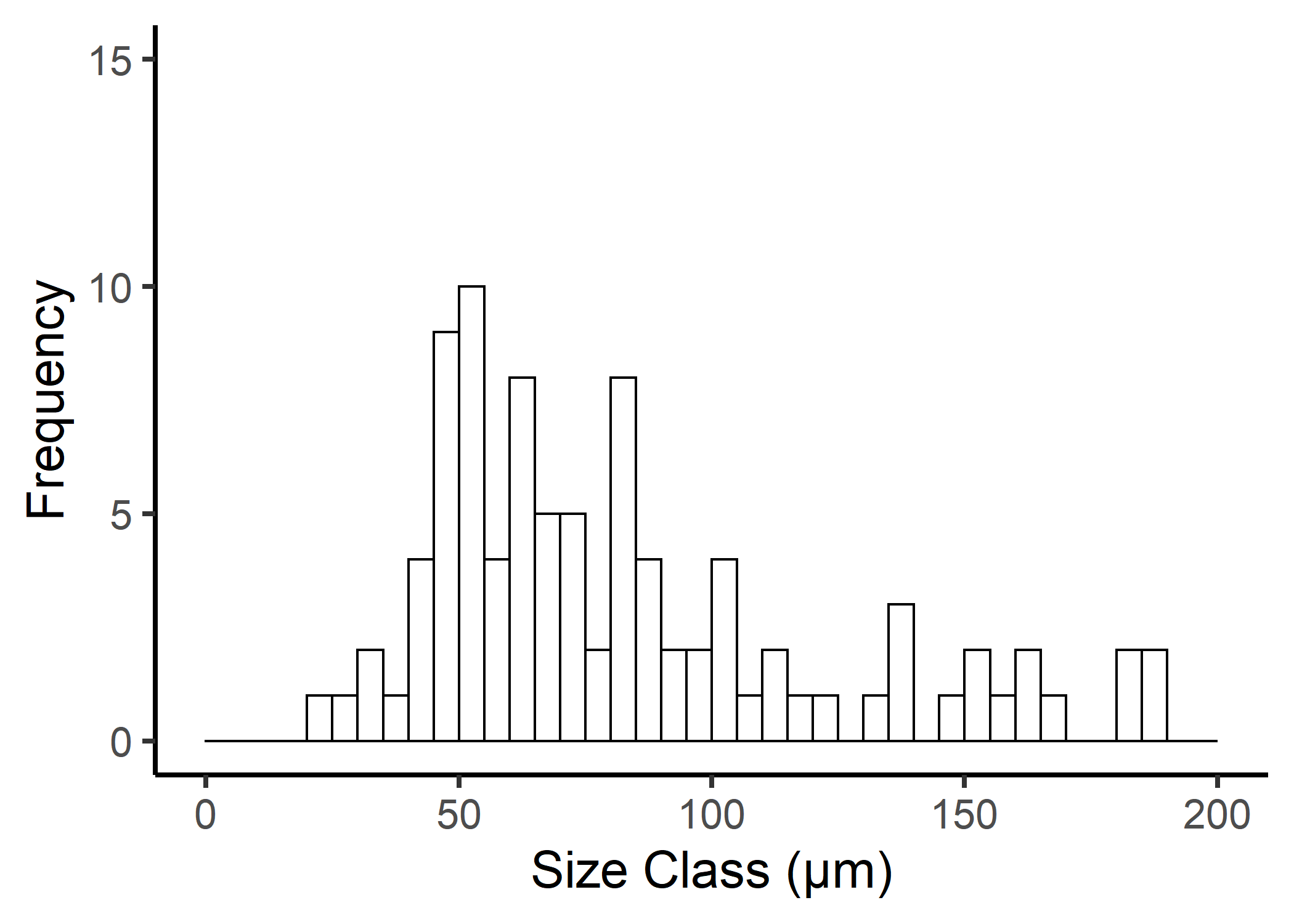

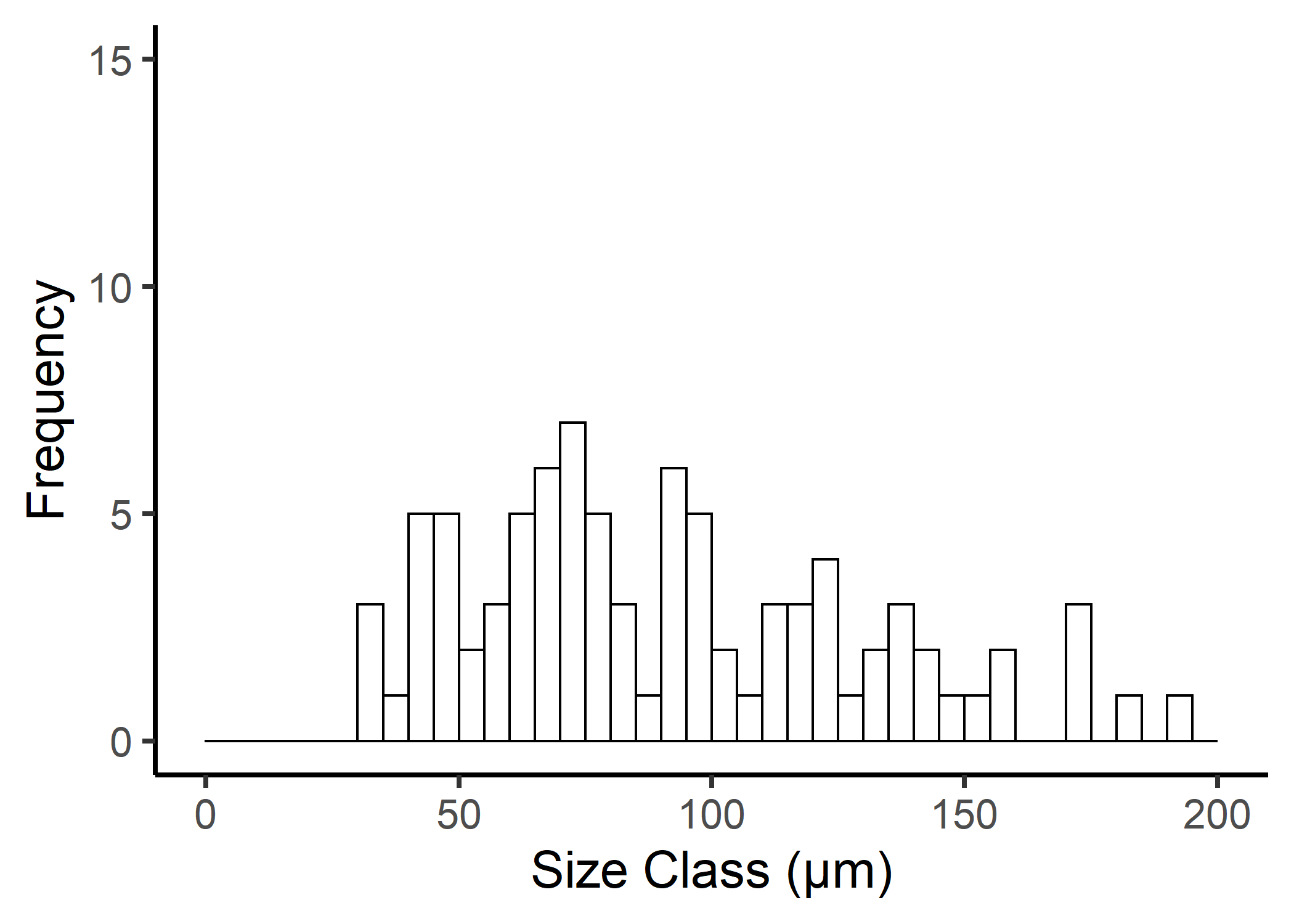

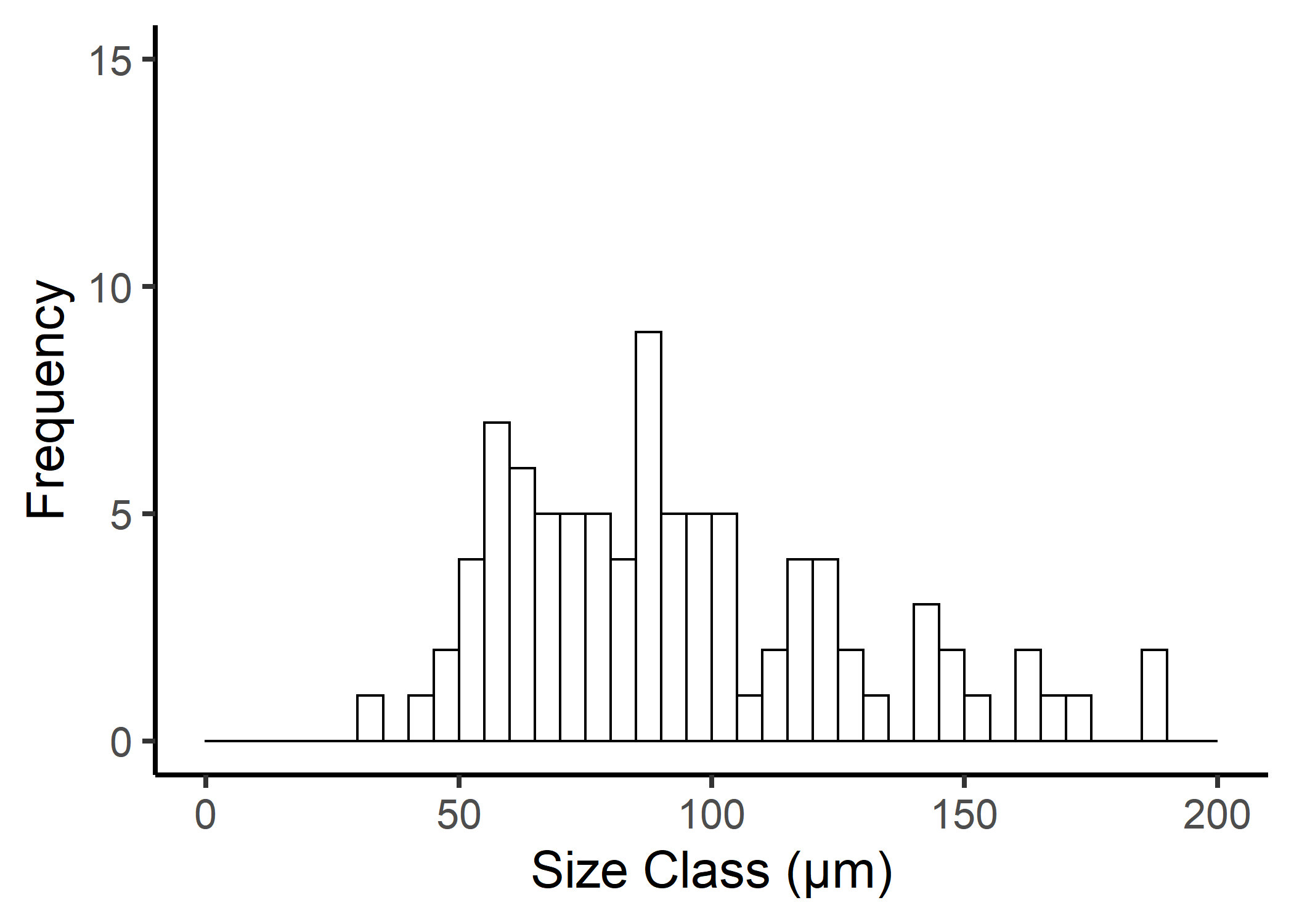

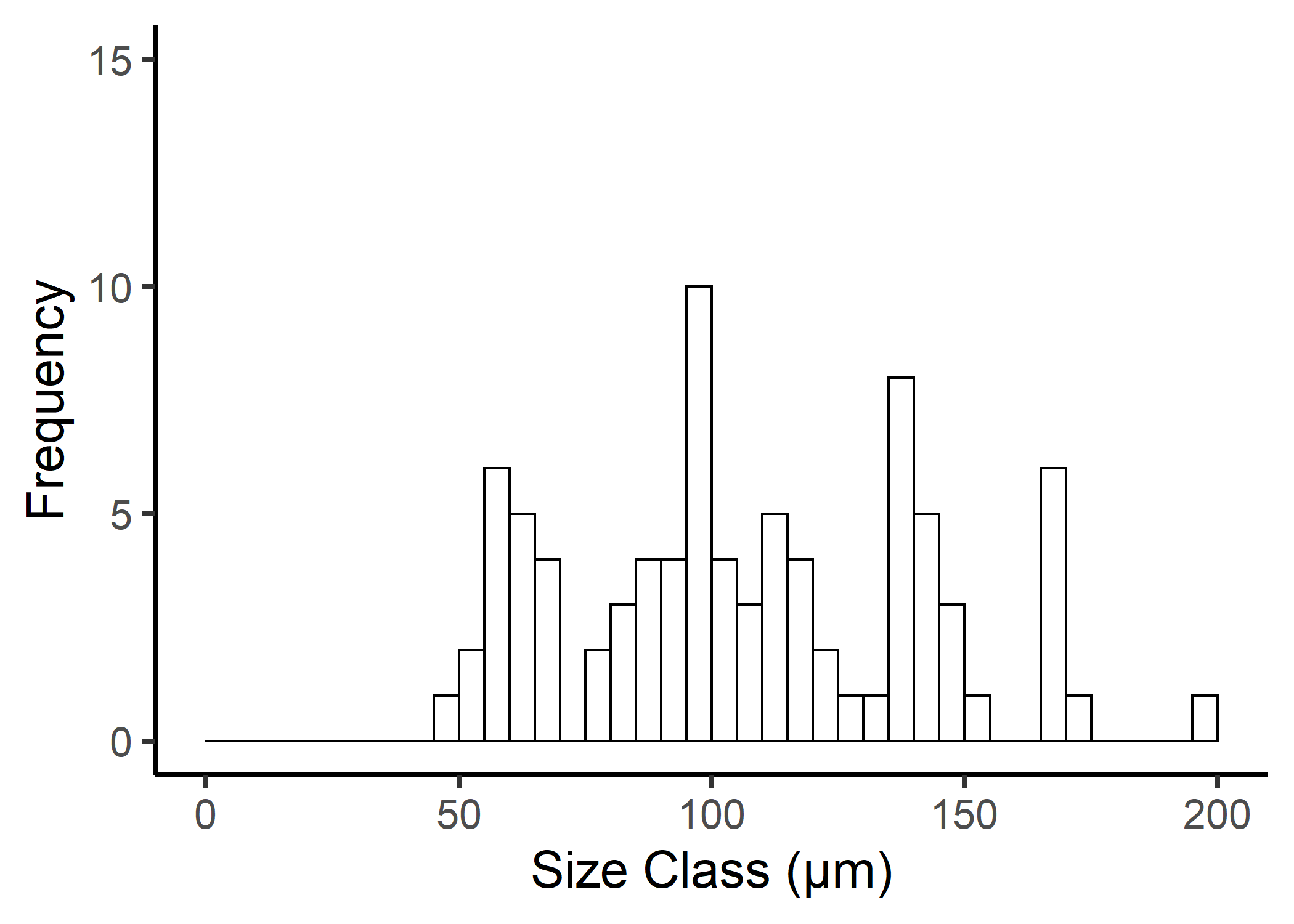

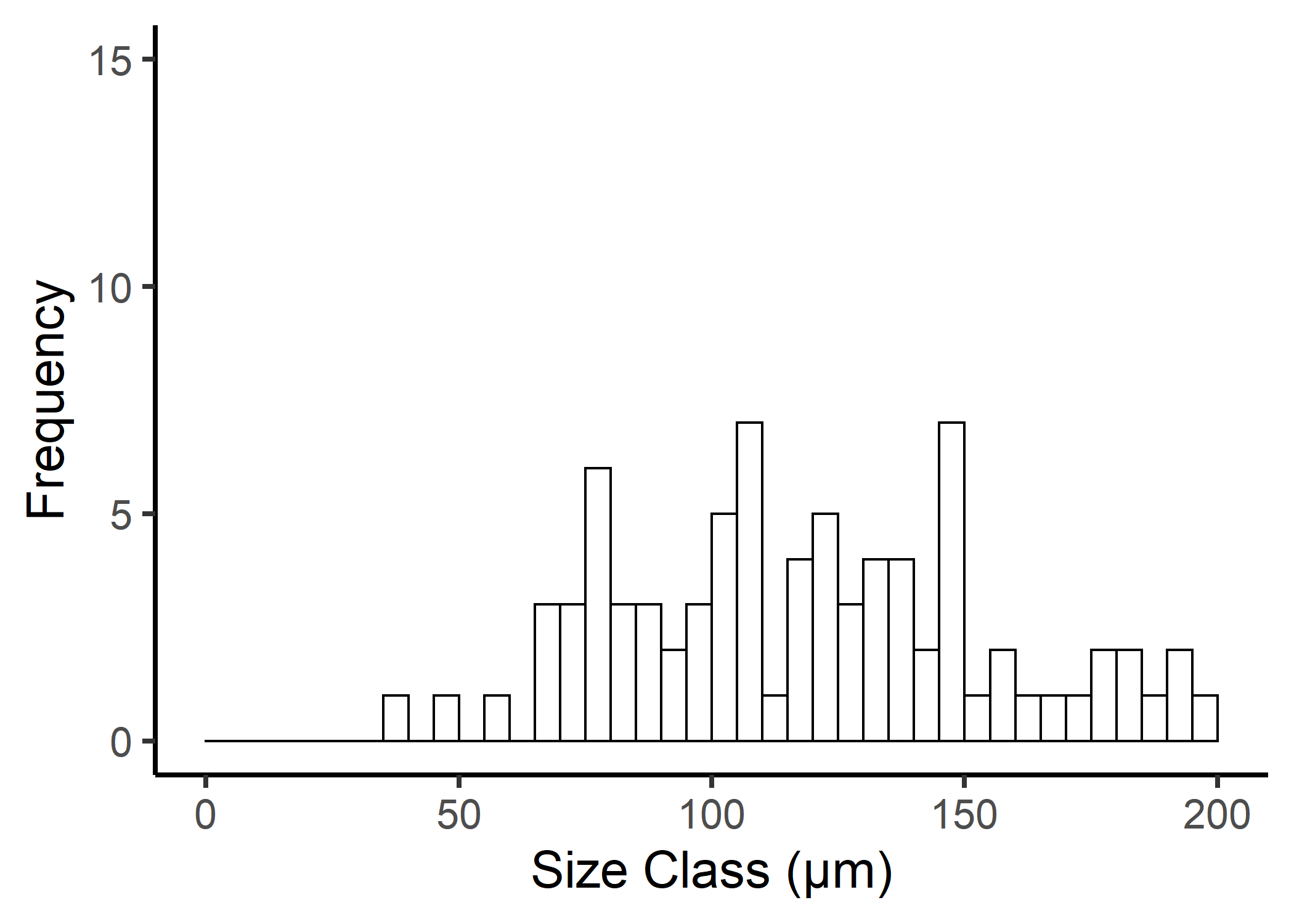

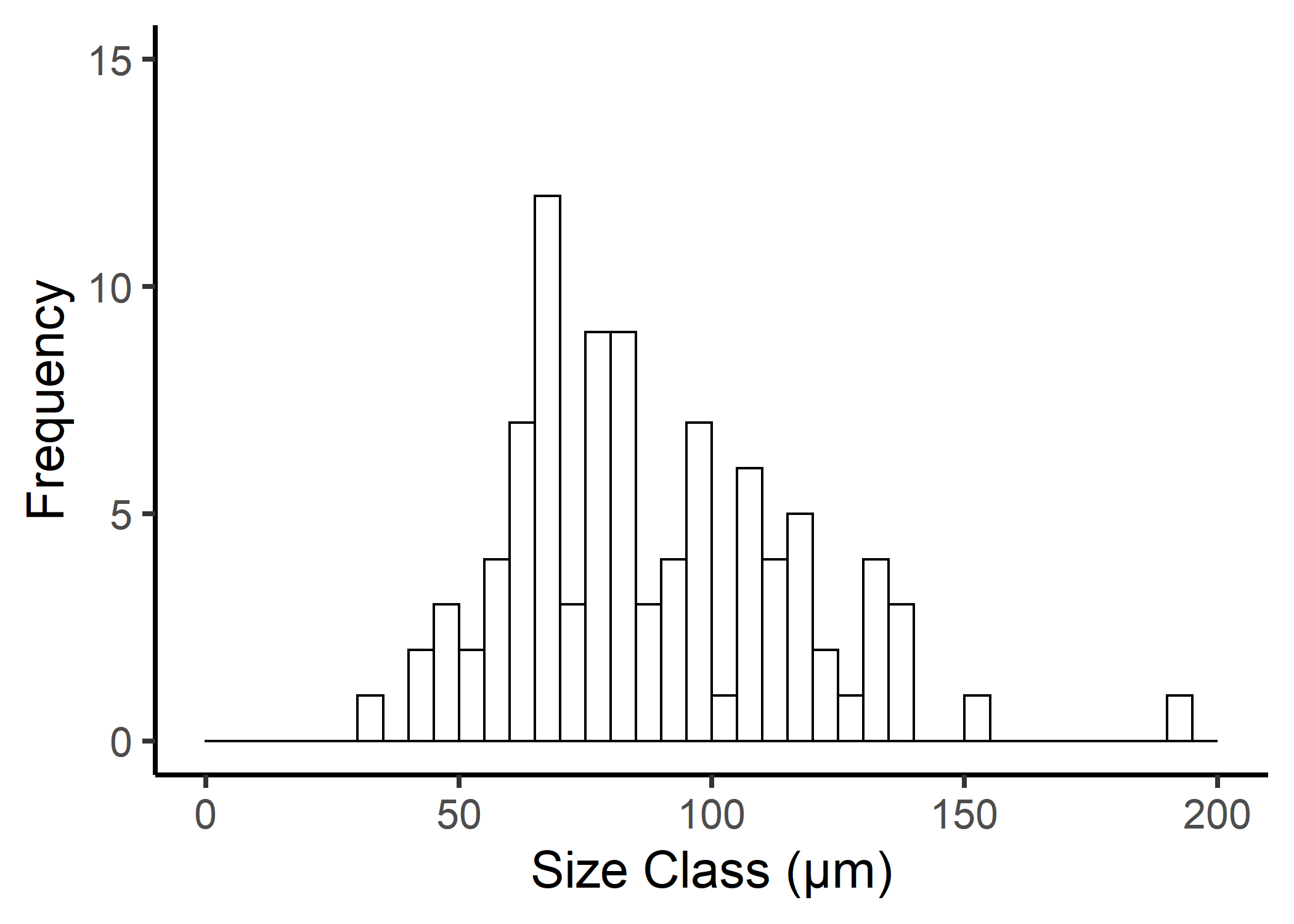

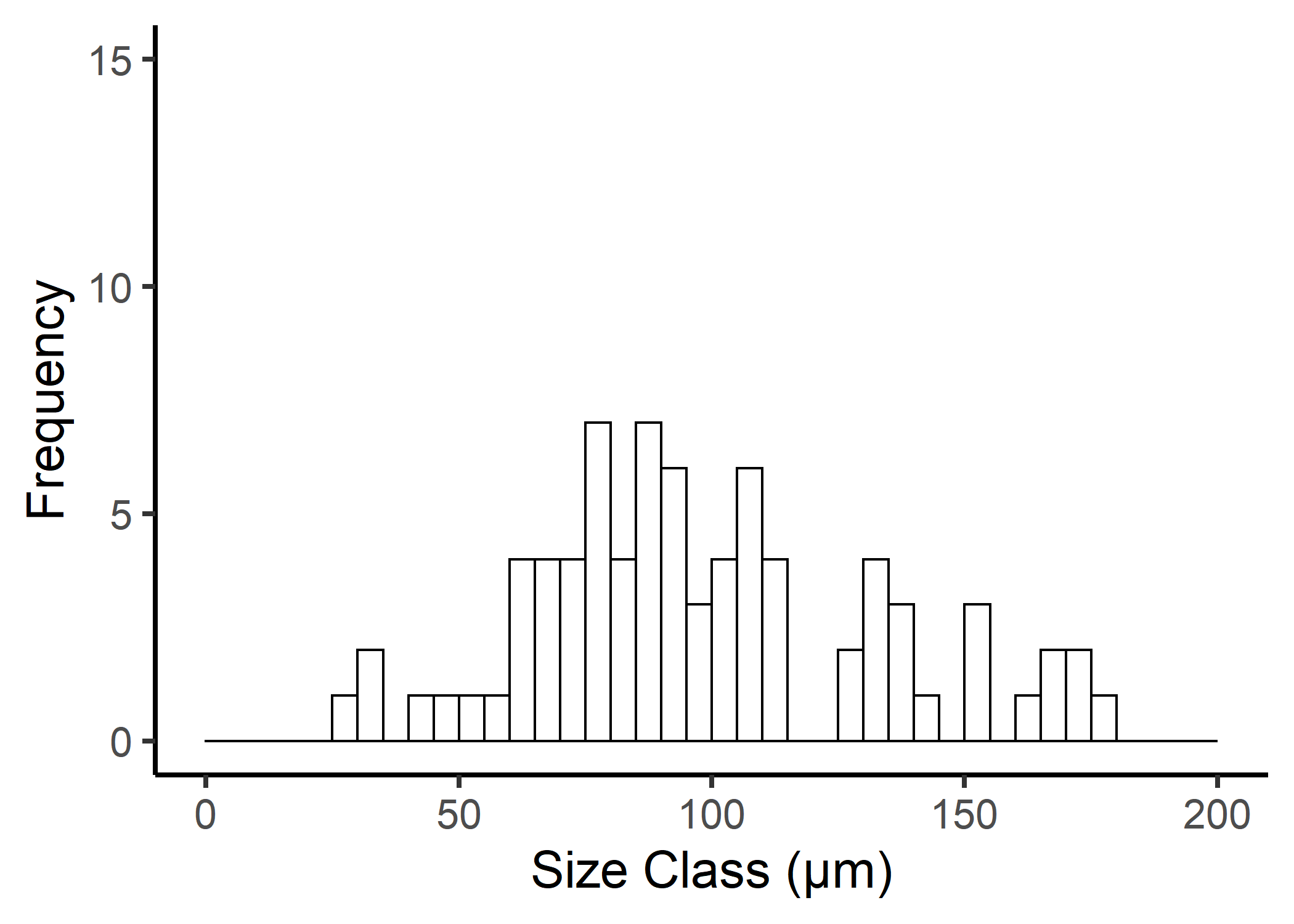


Figure S5. *Ctenodiscus crispatus* individual oocyte size frequencies at station B13


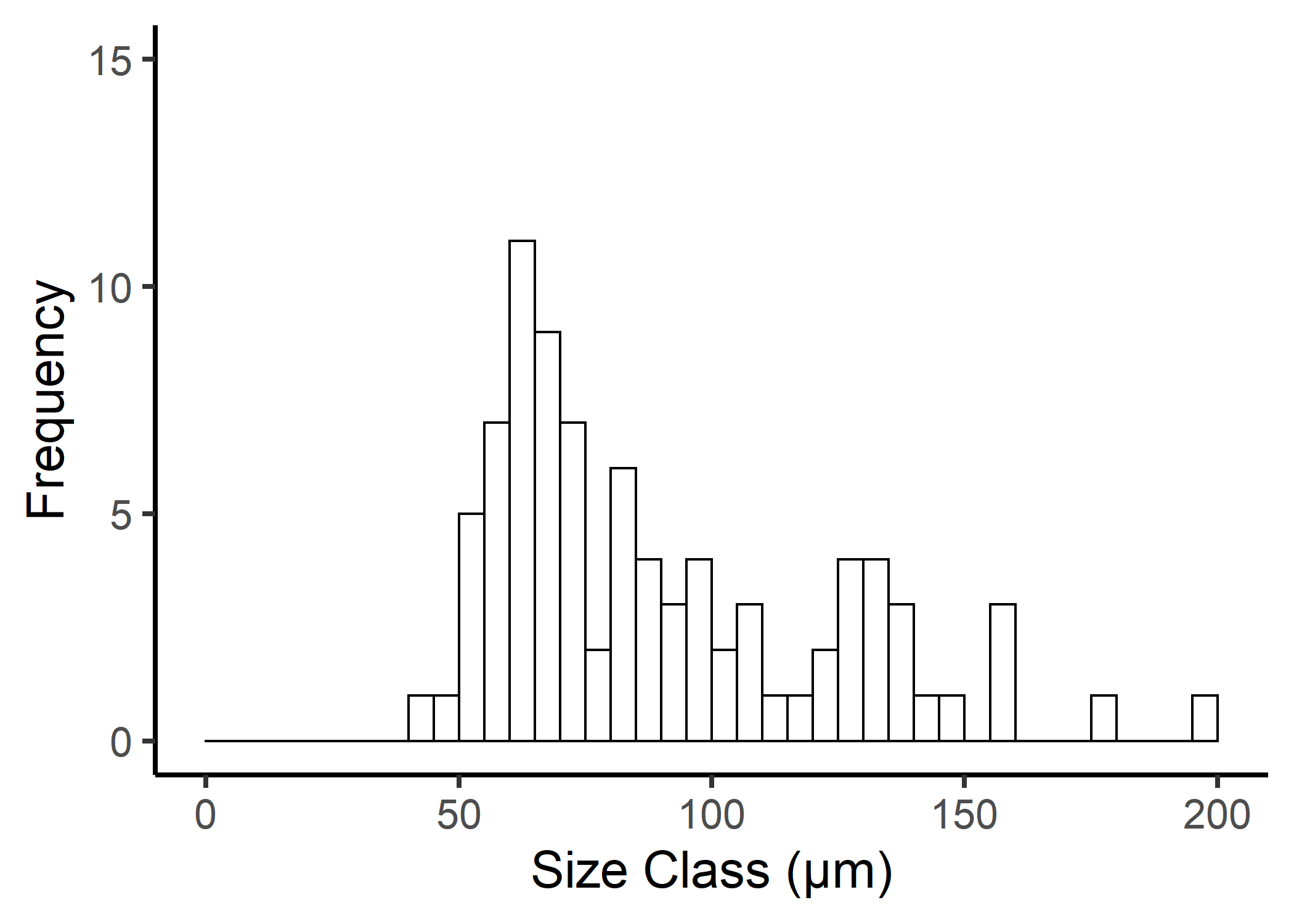

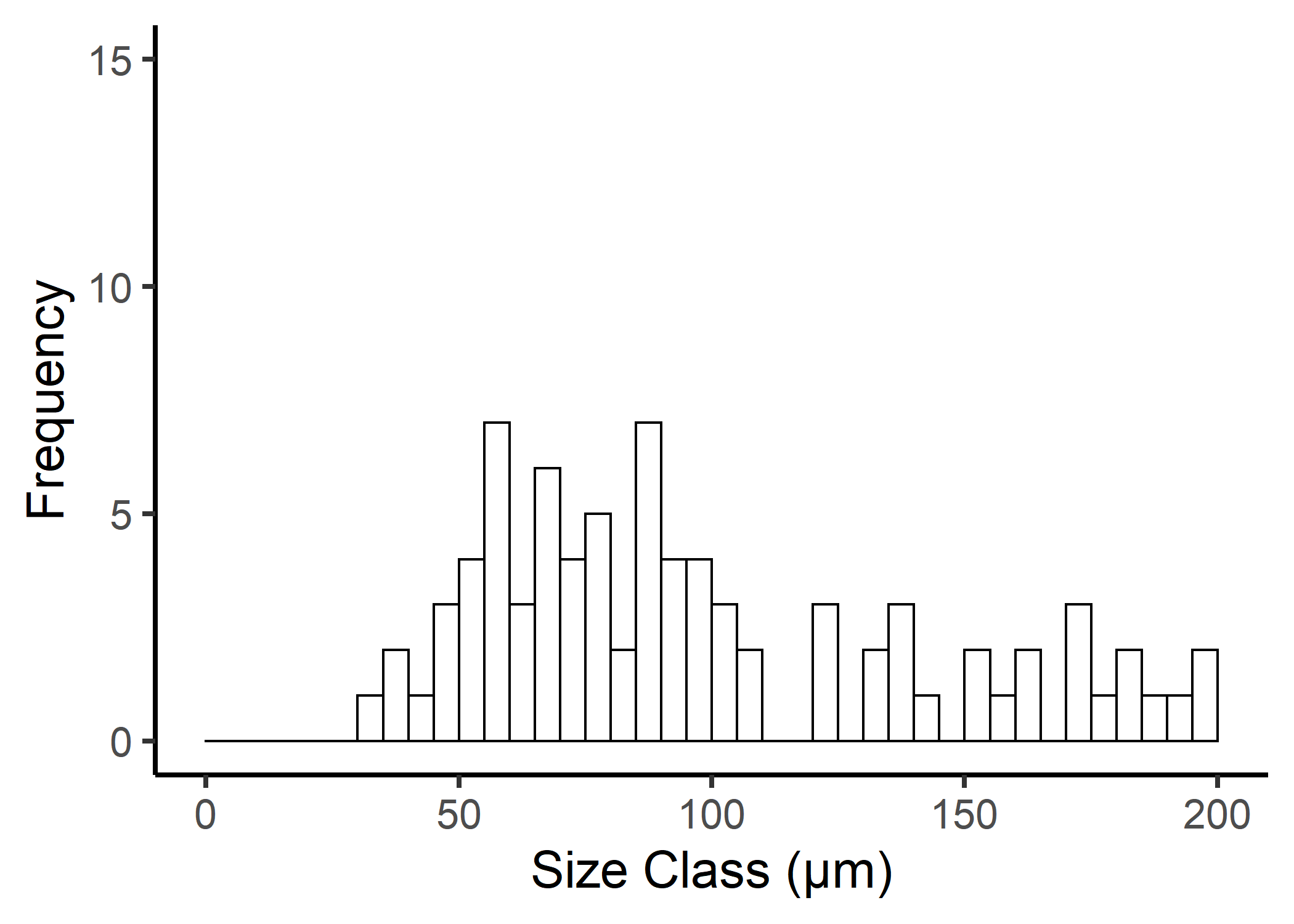

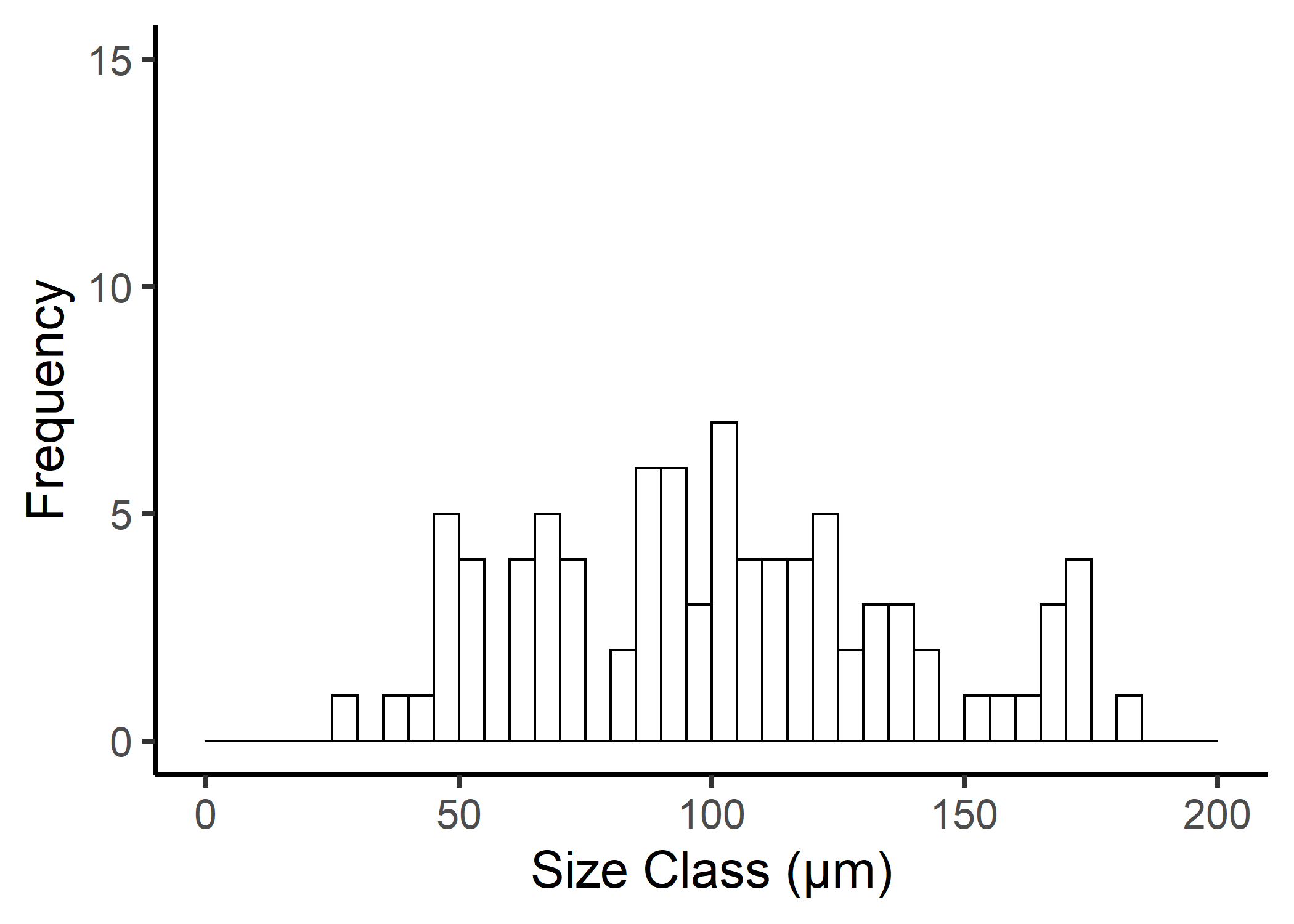

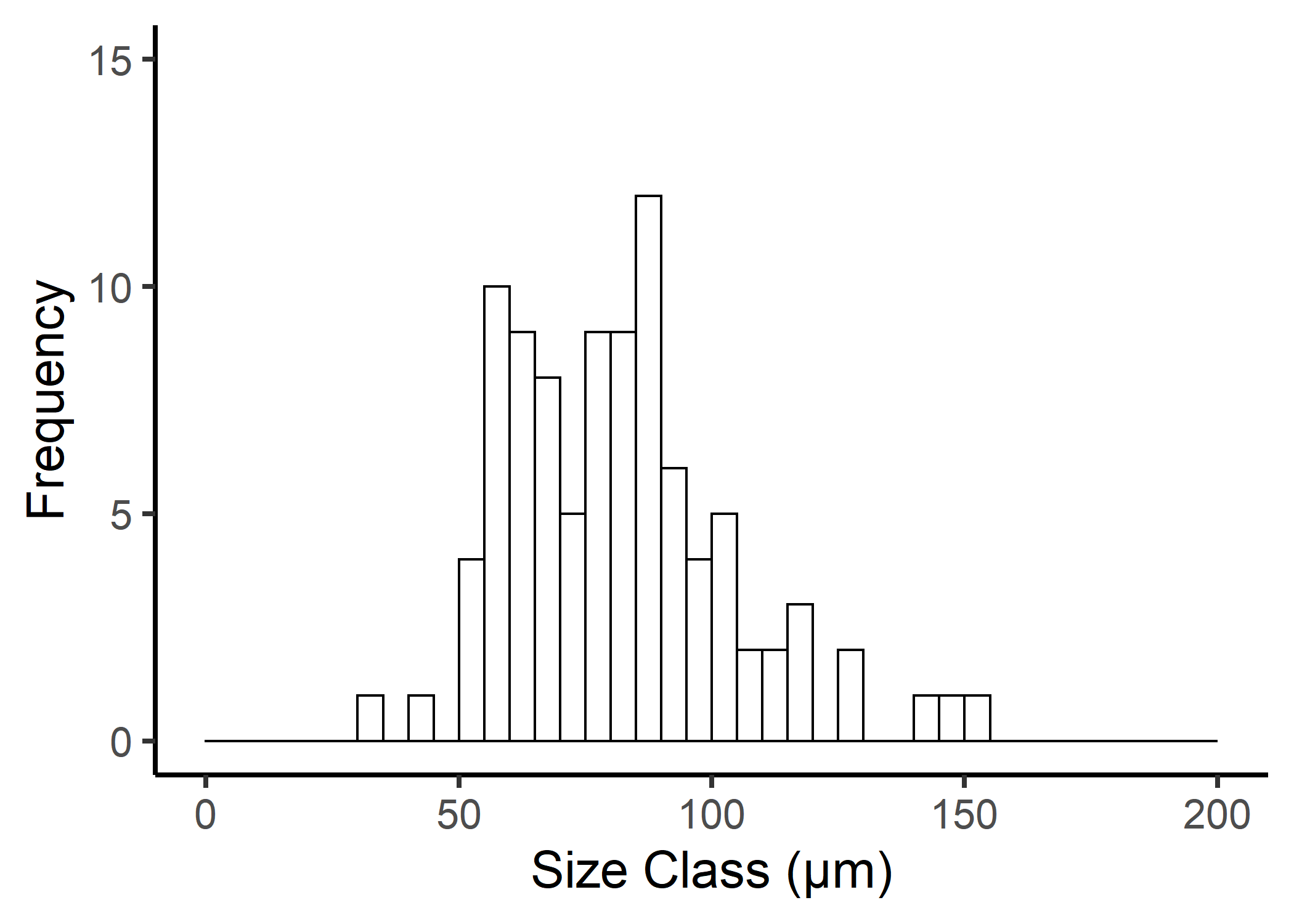

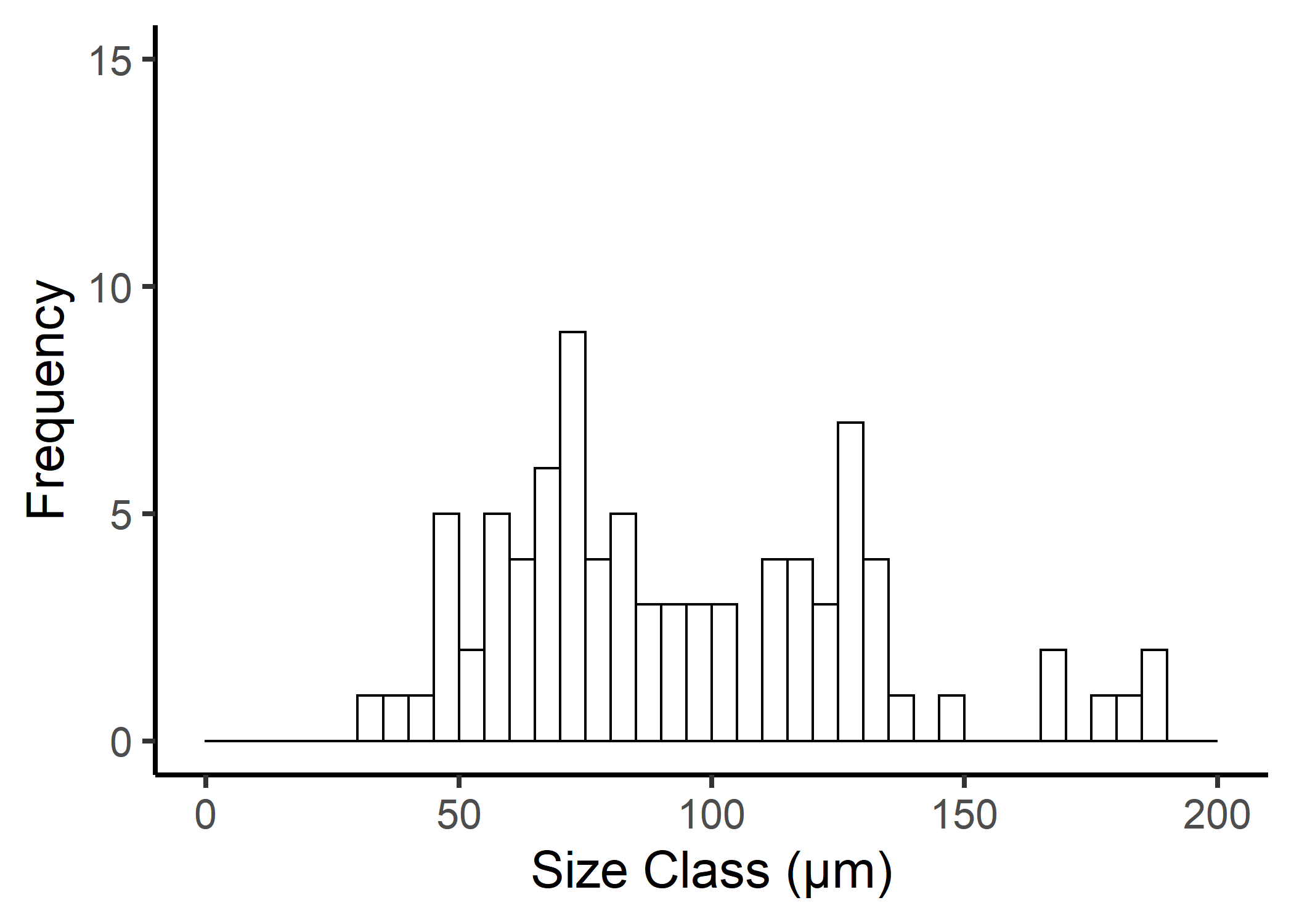

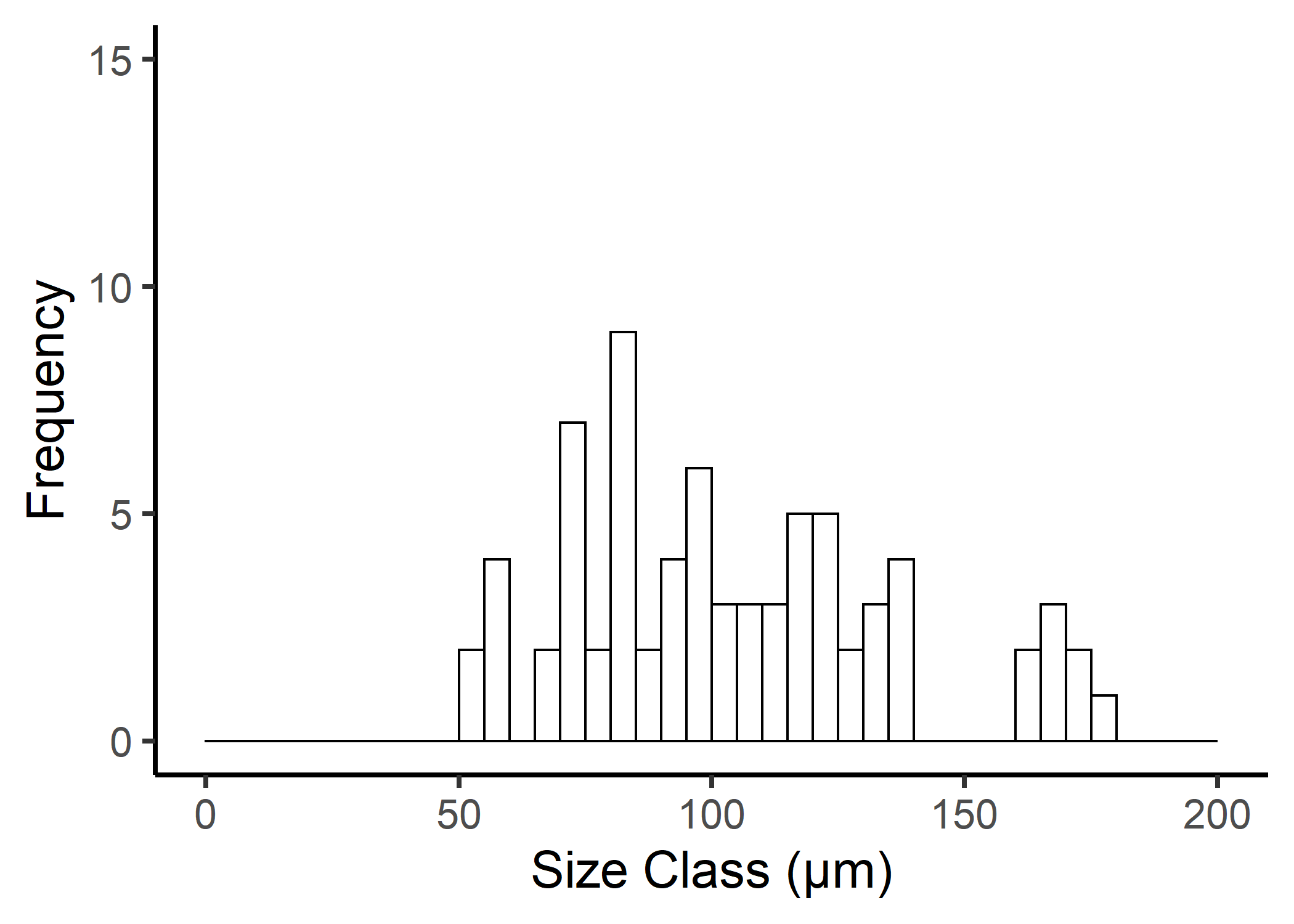

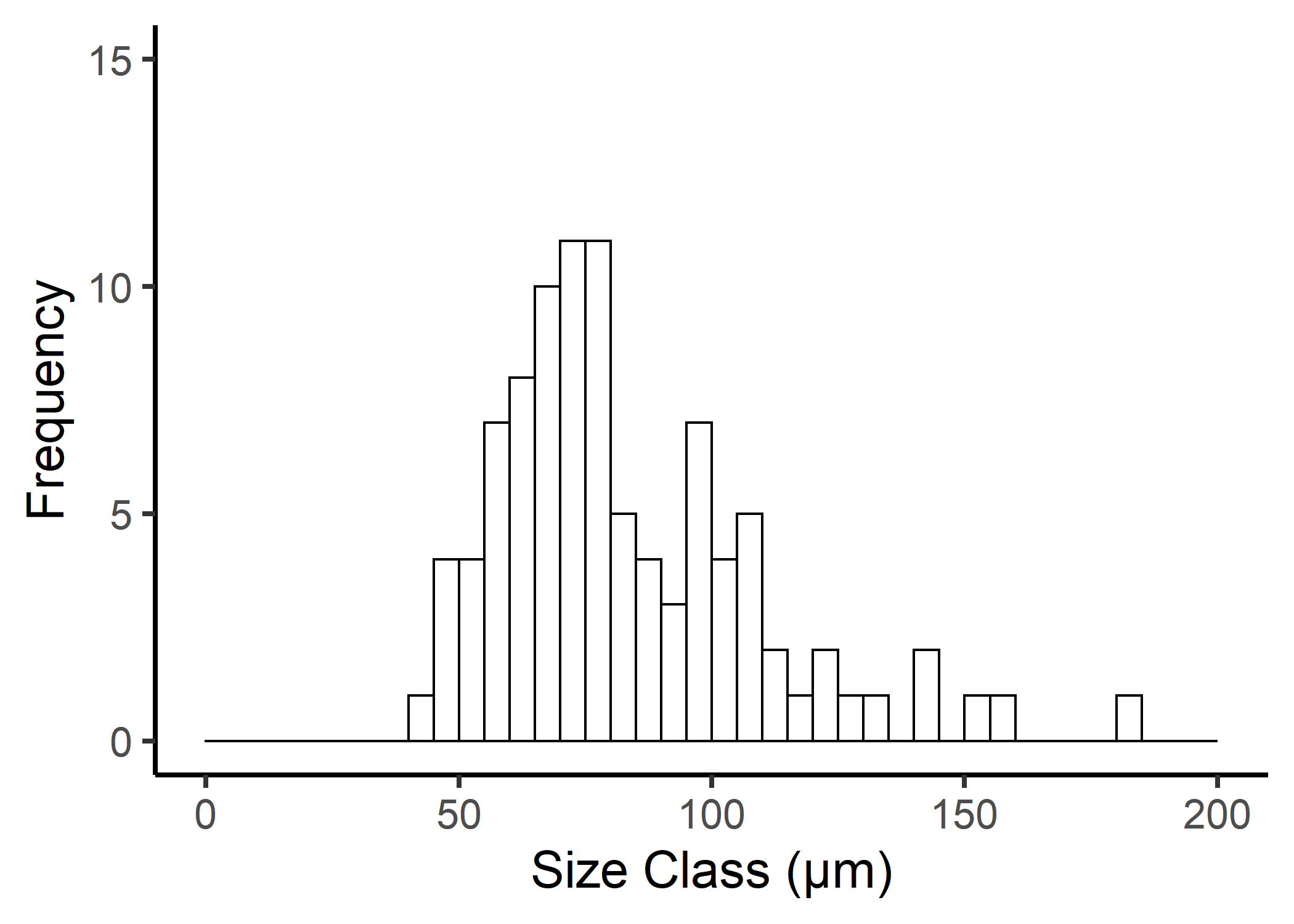

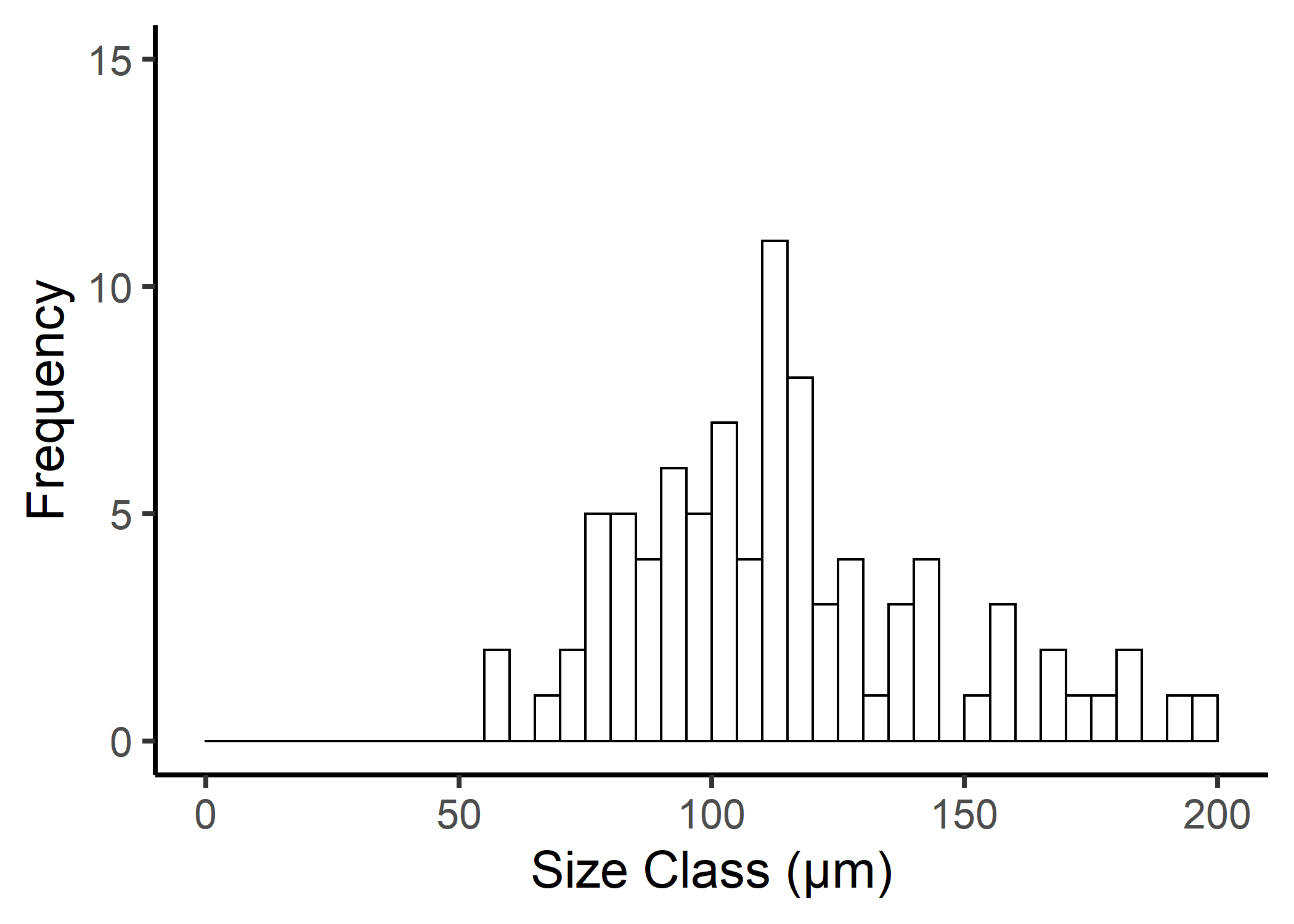


Figure S6. *Ctenodiscus crispatus* individual oocyte size frequencies at station B14


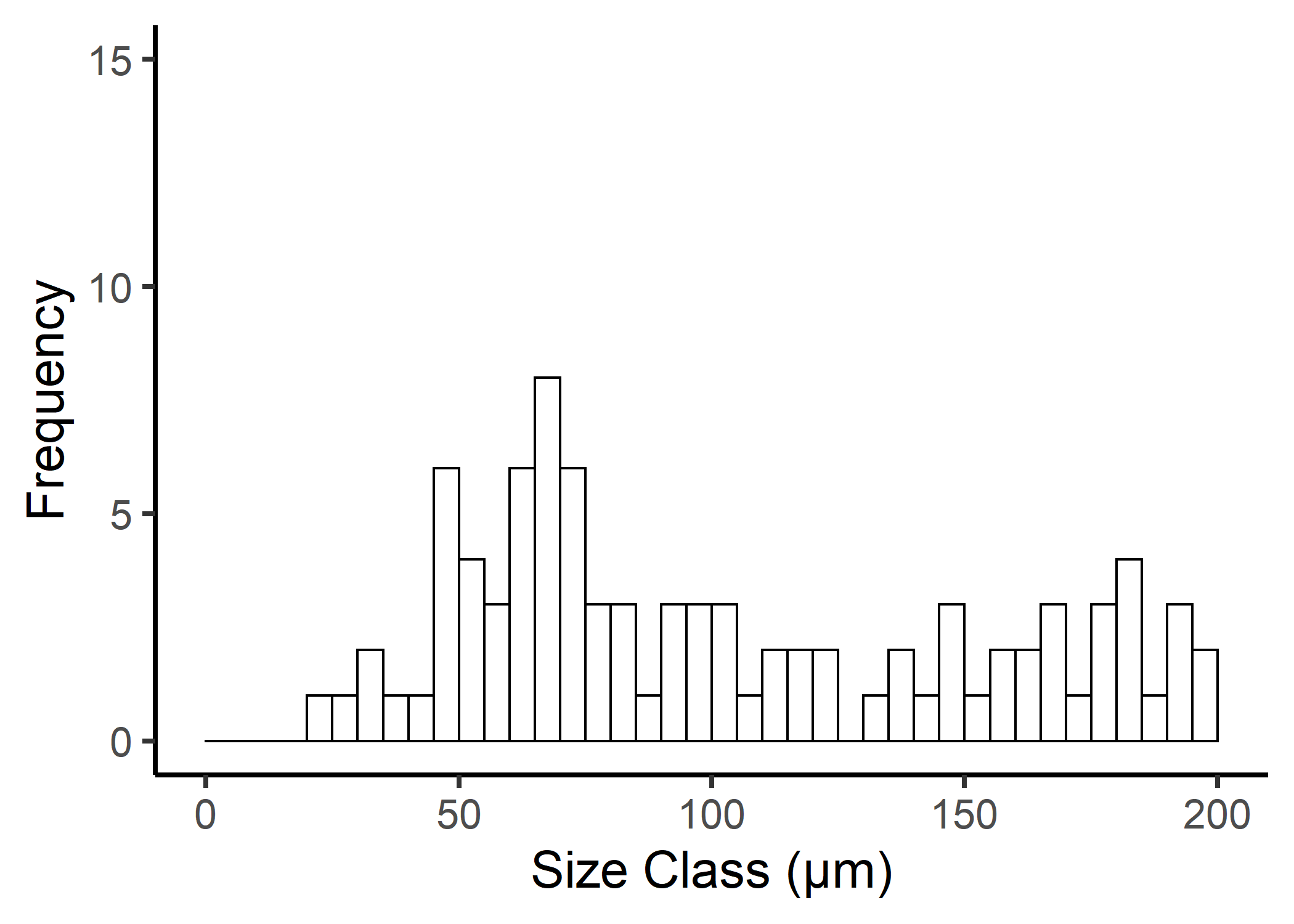

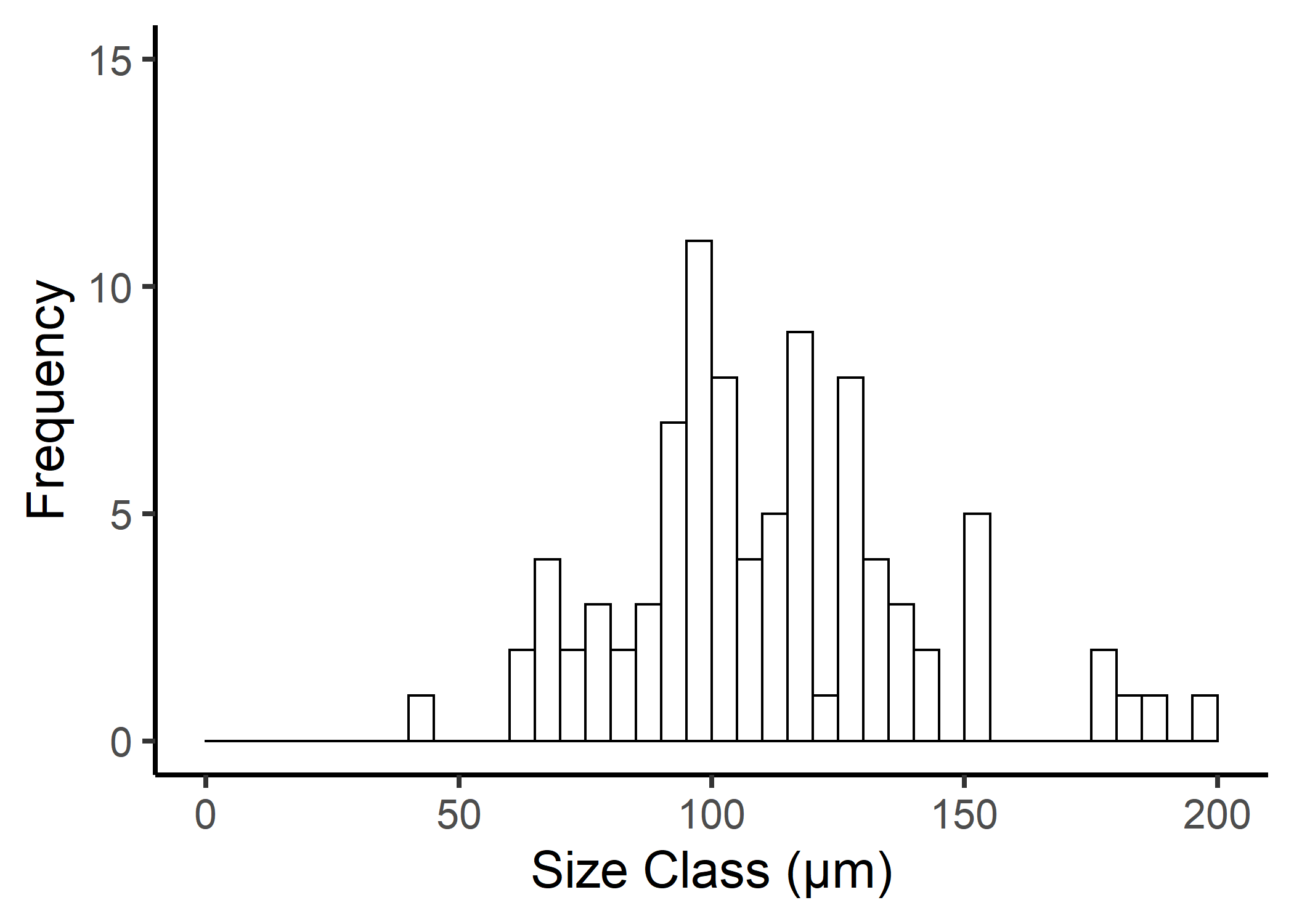

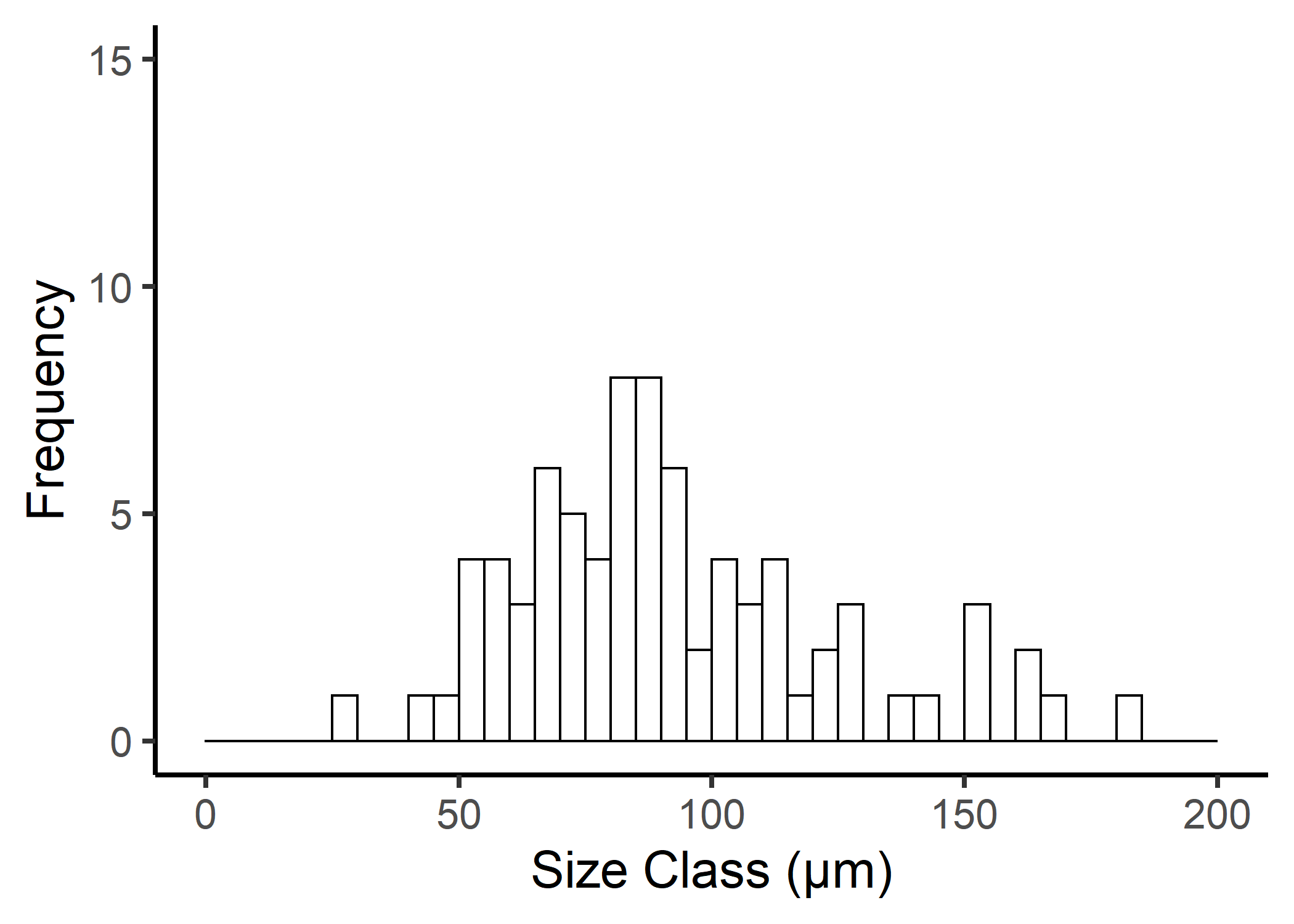

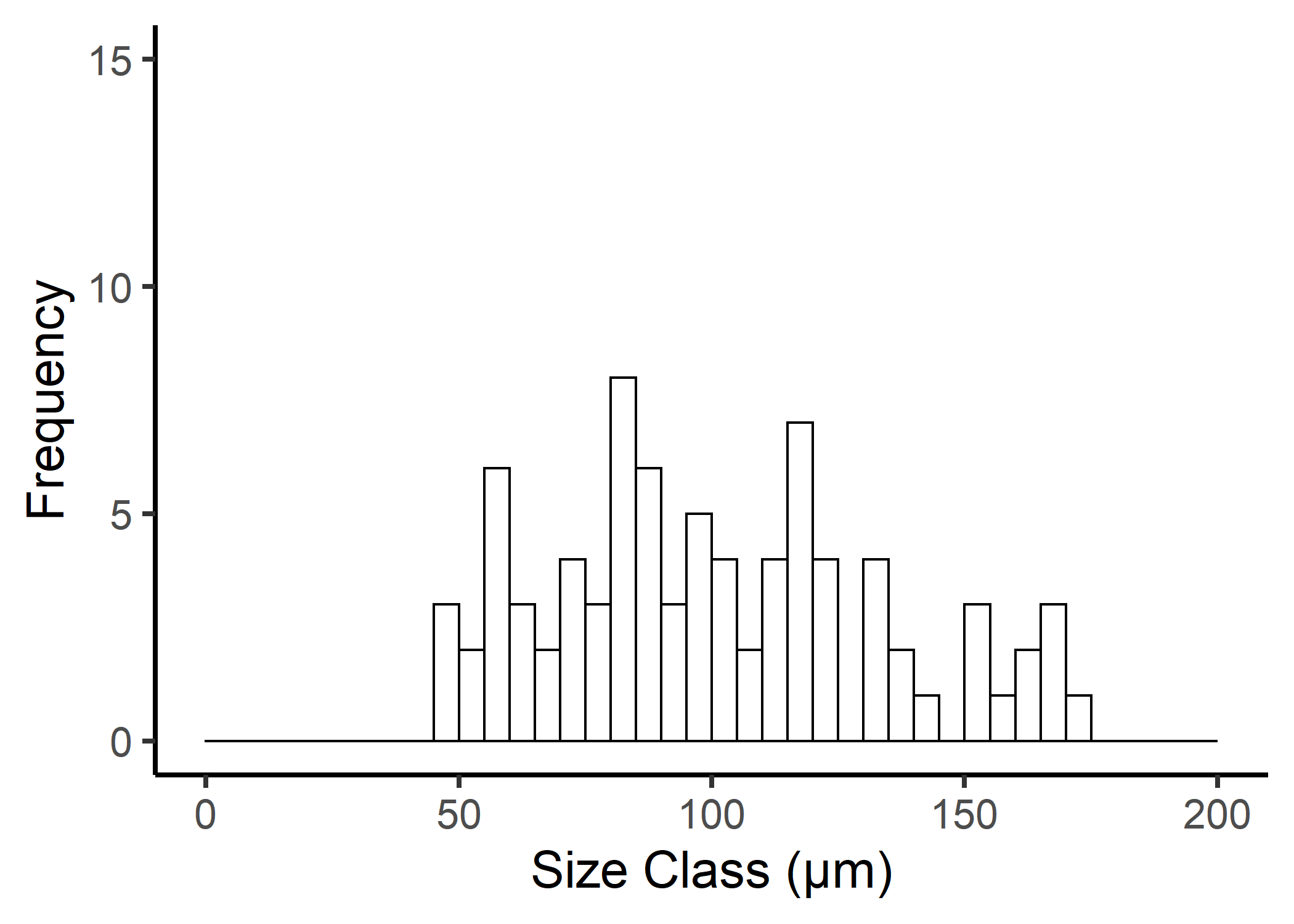

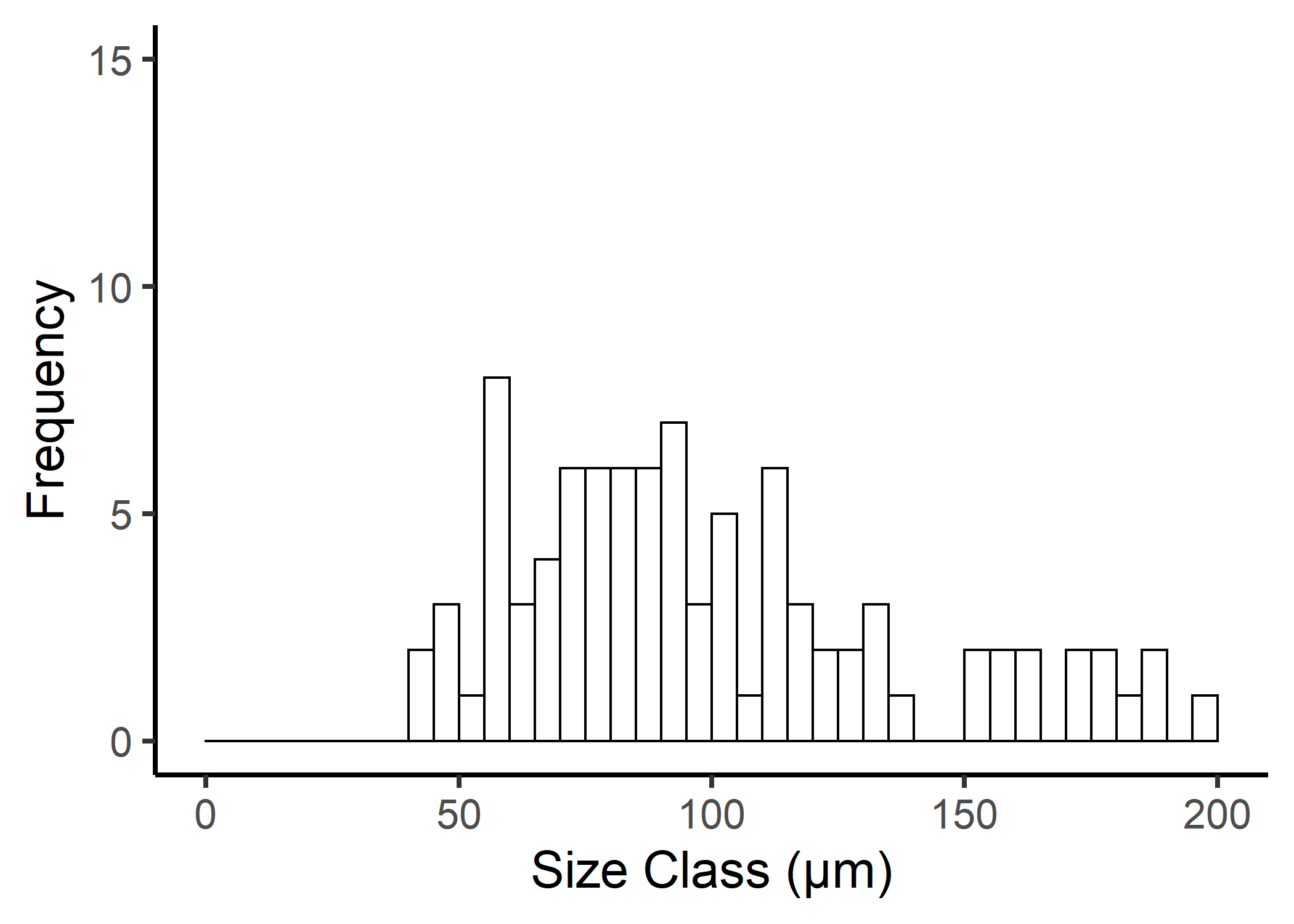

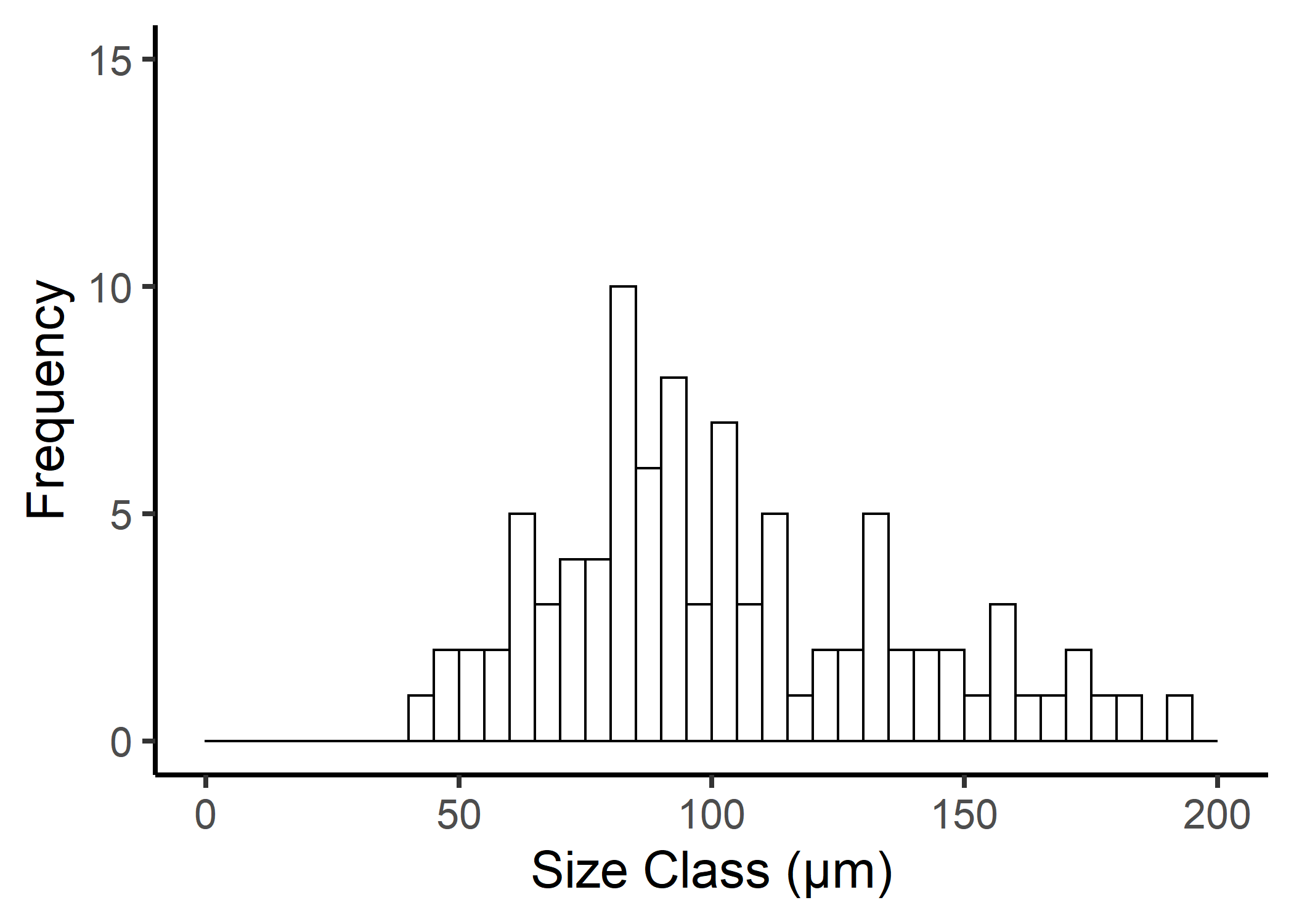

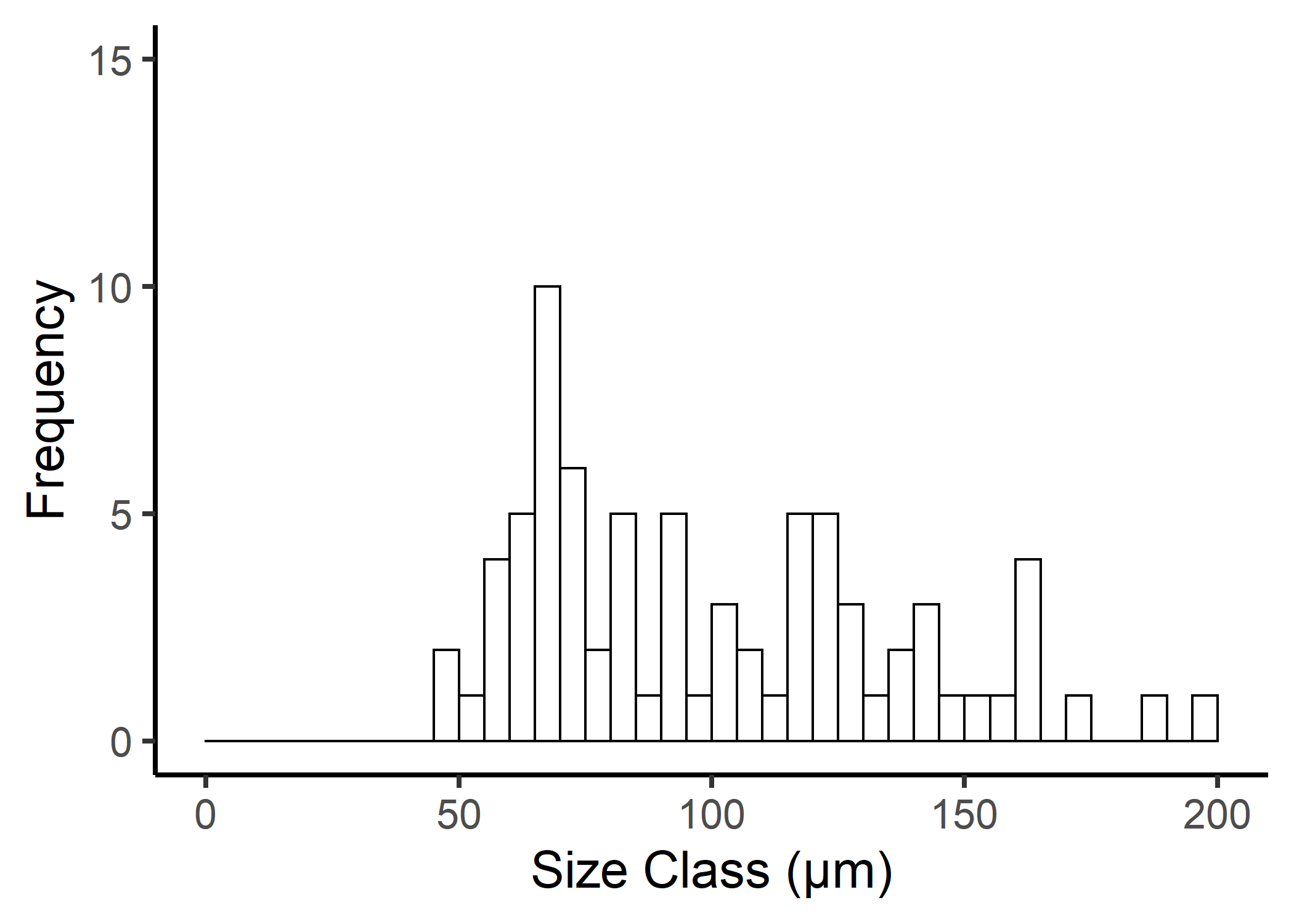

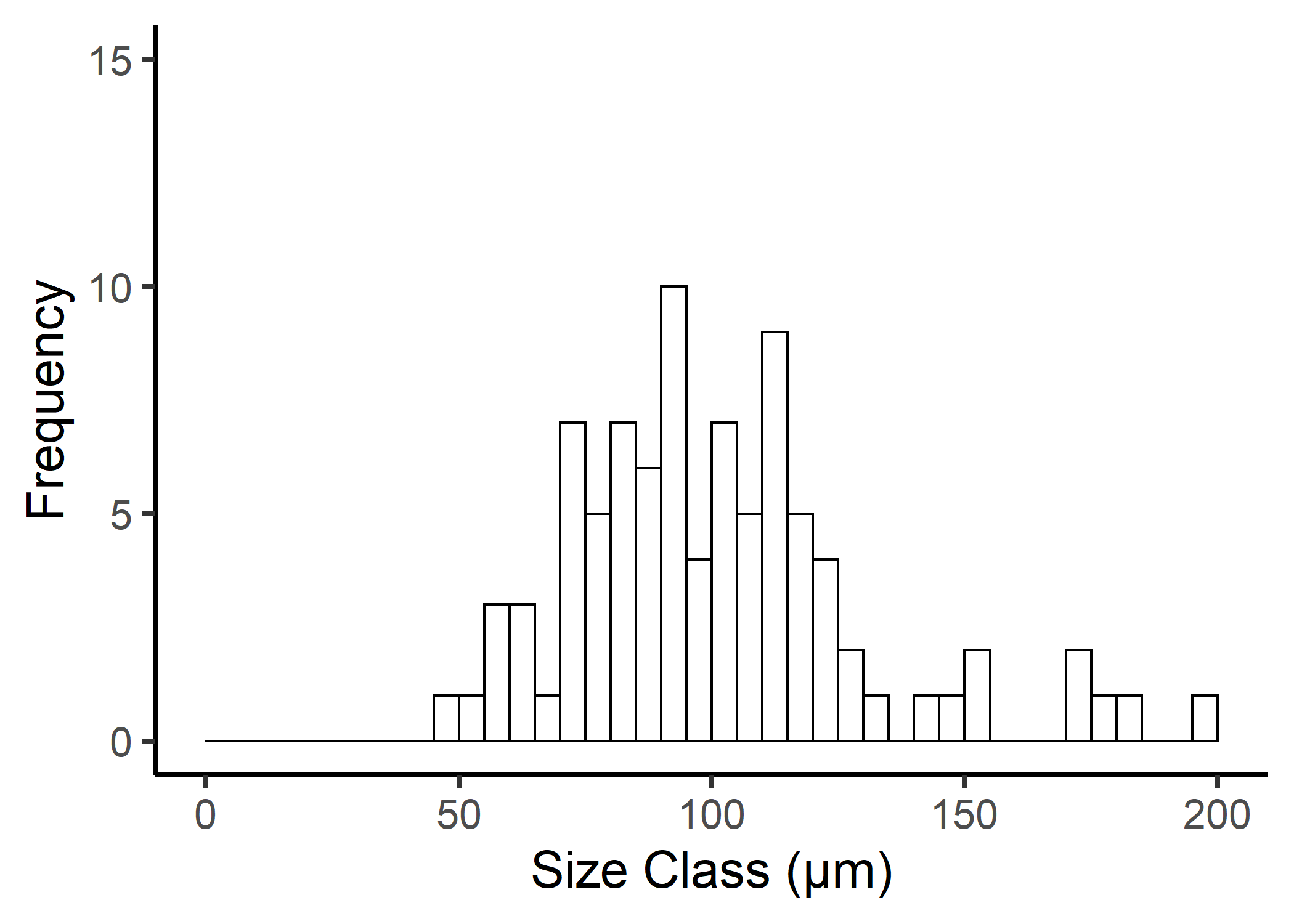


Figure S7. *Ctenodiscus crispatus* individual oocyte size frequencies at station B16

Figure S8. Morphological relationship between shell length and shell height for *Astarte crenata* along the 30ºE meridian (Stations: B13, orange; B16, blue) in the Barents Sea. Station positions are presented in Table S1 and Figure S1.


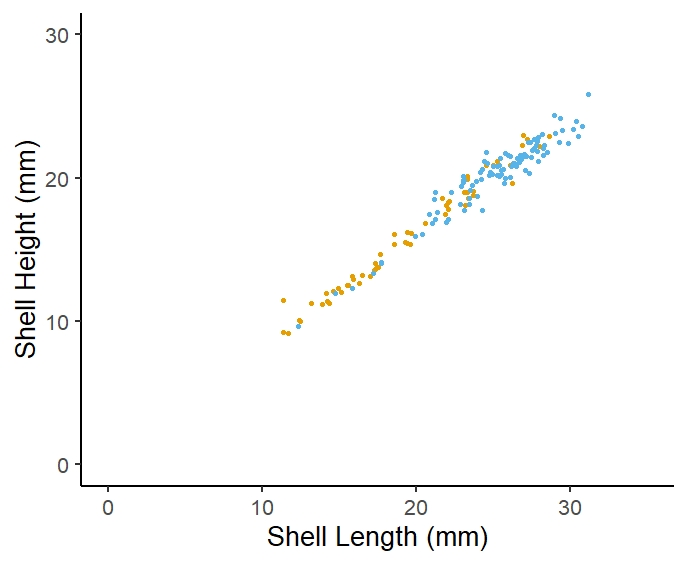


Figure S9. Morphological relationship between arm length and madreporite interradius for *Ctenodiscus crispatus* along the 30ºE meridian (Stations: B13, orange; B14, green; B16, blue) in the Barents Sea. Station positions are presented in Table S1 and Figure S1.


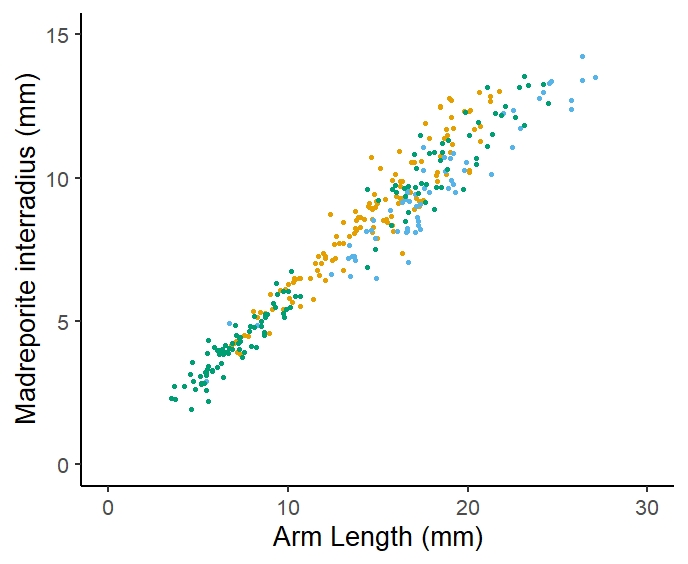


Figure S10. The daily sea ice extent values (km^2^) for 2^nd^ December 2014 to 28^th^ January 2020 at 1km grid cell size resolution for the Barents Sea region obtained from the Multisensor Analyzed Sea Ice Extent (MASIE) product (<https://nsidc.org/data/G02186/versions/1>, accessed 30/01/2020; Fetterer et al. 2010) at the National Snow and Ice Data Center (<http://nsidc.org/>). Years are indicated by colour (2014, black; 2015, red; 2016, green; 2017, blue; 2018, cyan; 2019, magenta; 2020, yellow). The time period of station occupancy for research cruise JCR16006 in 2017 is indicated by the grey line.


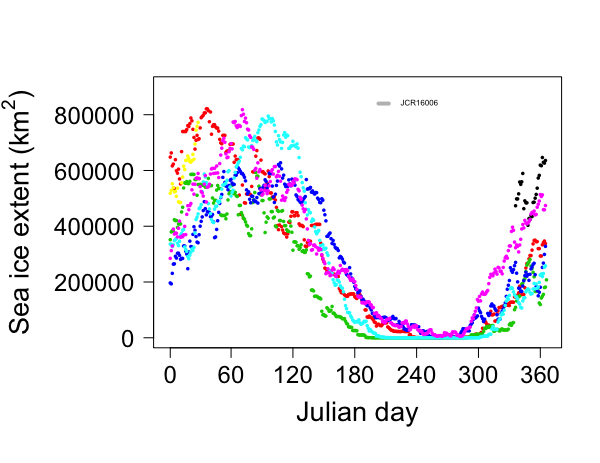


**References**

Fetterer F, Savoie M, Helfrich S, Clemente-Colón P. 2010, updated daily. Multisensor Analyzed Sea Ice Extent - Northern Hemisphere (MASIE-NH), Version 1. Boulder, Colorado USA. NSIDC: National Snow and Ice Data Center. doi: https://doi.org/10.7265/N5GT5K3K. [30/01/2020].

Grassle, J.F. 2000. The Ocean Biogeographic Information System (OBIS): an on‐line, worldwide atlas for accessing, modeling and mapping marine biological data in a multidimensional geographic context. Oceanogr. 13, 5–7.
